# Supplementary material for: Differential regulation of BIRC2 and BIRC3 expression by inflammatory cytokines and glucocorticoids in pulmonary epithelial cells
Source: PLoS One. 2023 Jun 8;18(6):e0286783. doi: 10.1371/journal.pone.0286783 (PMC10249814; doi:10.1371/journal.pone.0286783)
Supplement: S1 Table — Summary table highlighting the commonalities and differences between BIRC2 & BIRC3 expression and regulation found in this study. (PDF) [file pone.0286783.s001.pdf]

S1. Table. Summary of experimental findings

	BIRC3	BIRC2
Effect of proinflammatory cytokines (IL1B or TNF) on gene expression	• Highly upregulated mRNA and protein expression by both IL1B and TNF<br>• Peak expression seen at intermediate-late time points<br>• Consistent effects are shown in primary airway epithelial cells and A549 cells	• Lowly enhanced mRNA and minor increase in protein expression by both IL1B and TNF<br>• Expression is consistent and constitutive<br>• Consistent effects are shown in A549 cells and primary airway epithelial cells
Effect of Glucocorticoids (GC) on gene expression	• Modestly upregulated mRNA and protein expression<br>• Peak expression seen at intermediate-late time points<br>• Common expression patterns seen in primary cells, A549 and Calu-3 cells<br>• BEAS-2B cells show higher GC-inducibility	• No effect
Combinatorial effect of proinflammatory stimuli & Glucocorticoids	• No further enhanced effect with IL1B + GC<br>• Supra-additive mRNA and protein effect with TNF + GC<br>• Supra-additive effect shown in cell lines and primary cells<br>• Data reveals possible cooperative interaction between inflammatory stimulus and GC	• No effect
Is NF-κB required for gene induction by proinflammatory cytokines?	• IKK2 inhibition shows marked loss of expression<br>• Canonical NF-κB is otherwise required	• NF-κB is required but failure to abolish expression suggest there may include IKK2-independent mechanisms
Does glucocorticoid-induced gene expression require GR?	• GR is required for GC-induced gene expression<br>• GR is necessary for proinflammatory and GC cooperative BIRC3 expression	• N/A
Effect of cycloheximide (CHX) on protein stability	• IL1B-plus-CHX blocked inducible protein expression<br>• TNF-plus-CHX further reduced protein expression below resting conditions<br>• CHX had no effect once peak BIRC3 protein had been reached	• IL1B-plus-CHX blocked inducible protein expression<br>• TNF-plus-CHX further reduced protein expression below resting conditions
Contribution of the 26S proteasome	• Blockage of the 26S proteasome rescued loss of IL1B or TNF dependent loss of protein expression<br>• Data shows protein turnover includes the 26S proteasome	• Blockage of the 26S proteasome rescues loss of IL1B or TNF dependent loss of protein expression<br>• Data shows protein turnover includes the 26S proteasome
