# Supplementary material for: Differential regulation of BIRC2 and BIRC3 expression by inflammatory cytokines and glucocorticoids in pulmonary epithelial cells
Source: PLoS One. 2023 Jun 8;18(6):e0286783. doi: 10.1371/journal.pone.0286783 (PMC10249814; doi:10.1371/journal.pone.0286783)
Supplement: S1 File — A549, BEAS2-B, Calu-3, pHBEC-SC and pHBEC-ALI cells were treated with proinflammatory cytokines and glucocorticoids at specific times as per the in-text captions. Included are dose-response curves for IL1B and TNF, siRNA silencing for RELA and GR, proteasome inhibitors and the effect of cycloheximide on K48-Ub. Data for mRNA expression, protein expression and NF-κB- or GRE-dependent luciferase activity are shown. Each figure has a legend detailing the experimental parameters. (PDF) [file pone.0286783.s002.pdf]

Supporting Information S1-8 Fig

Differential regulation of BIRC2 and BIRC3 expression by inflammatory cytokines and glucocorticoids in pulmonary epithelial cells

Andrew Thorne[1], Akanksha Bansal[1], Amandah Necker-Brown[1], Mahmoud Mostafa[1], Alex Gao[1], Andrei Georgescu[1], Cora Kooi[2], Richard Leigh[1,2] and Robert Newton[1,*]

[1] Department of Physiology & Pharmacology and Lung Health Research Group, Snyder Institute for Chronic Diseases, Cumming School of Medicine, and [2] Department of Medicine and Airways Inflammation Research Group, Snyder Institute for Chronic Diseases, University of Calgary, Calgary, Alberta, Canada

*Corresponding author
E-mail: rnewton@ucalgary.ca

Supplemental Fig. S1

A549 6kB.tk: luciferase activity at 6 h

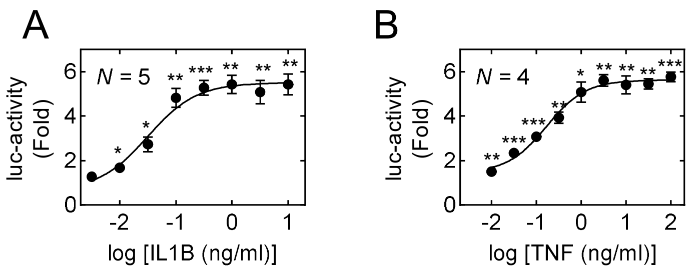

S1 Fig. Activation of NF-κB-dependent transcription by IL1B or TNF. A549 cells harbouring the NF-κB-dependent reporter, 6κBtkluc.neo, were either not stimulated or treated with increasing concentrations of: **(A)** IL1B or, **(B)** TNF. After 6 h, cells were harvested for luciferase activity determination. Data from $N = 4$-5 experiments were expressed as fold of untreated and plotted as means ±SE. Significance relative to untreated was tested by one-way ANOVA with a Dunnett's post-hoc test. * $p \leq 0.05$, ** $p \leq 0.01$, *** $p \leq 0.001$.

Supplemental Fig. S2

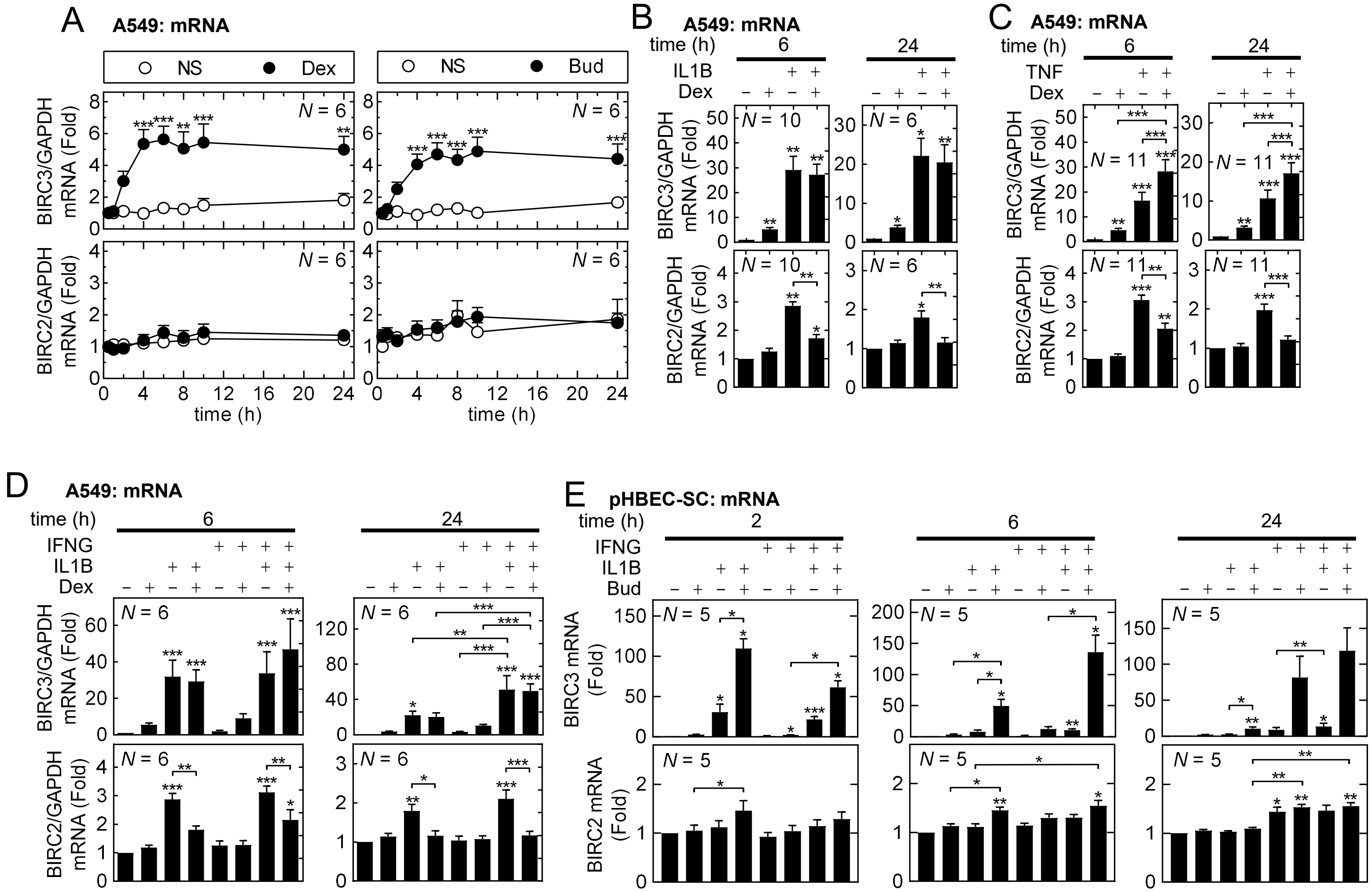

S2 Fig. Effects of inflammatory cytokines and glucocorticoids on BIRC2 and BIRC3 mRNA expression in A549 cells or pHBEC-SC. (A) A549 cells were not stimulated (NS) or treated with dexamethasone (*Dex*; 1 µM) or budesonide (*Bud*; 300 nM). Cells were harvested at the indicated times for qPCR analysis. Data for BIRC3 and BIRC2 were normalized to GAPDH, expressed as fold of NS at t = 1 h, and plotted as mean ±SE. **(B-D)** A549 cells were not stimulated (NS) or treated with IL1B (1 ng/ml), TNF (10 ng/ml), IFNG (10 ng/ml), and/or dexamethasone (*Dex*; 1 µM). Cells were harvested at 6 or 24 h for qPCR analysis. Data for BIRC3 and BIRC2 were normalized to GAPDH, expressed as fold of NS and plotted as mean ±SE. **(E)** pHBEC-SC were not stimulated (NS) or treated with IL1B (1 ng/ml), IFNG (10 ng/ml), and/or budesonide (*Bud*; 300 nM). Cells were harvested at 2, 6 or 24 h for qPCR analysis. Data for BIRC3 and BIRC2 were normalized to GAPDH, expressed as fold of NS, and plotted as mean ±SE. In all panels data from $N = 5$-11 experiments are shown. Significance was tested against NS at each time point using one-way ANOVA with a Dunnett's in *A,* or by Tukey's post-hoc test in *B-E*. * $p \leq 0.05$, ** $p \leq 0.01$ and *** $p \leq 0.001$ indicates significance relative to NS or as indicated.

Supplemental Fig. S3

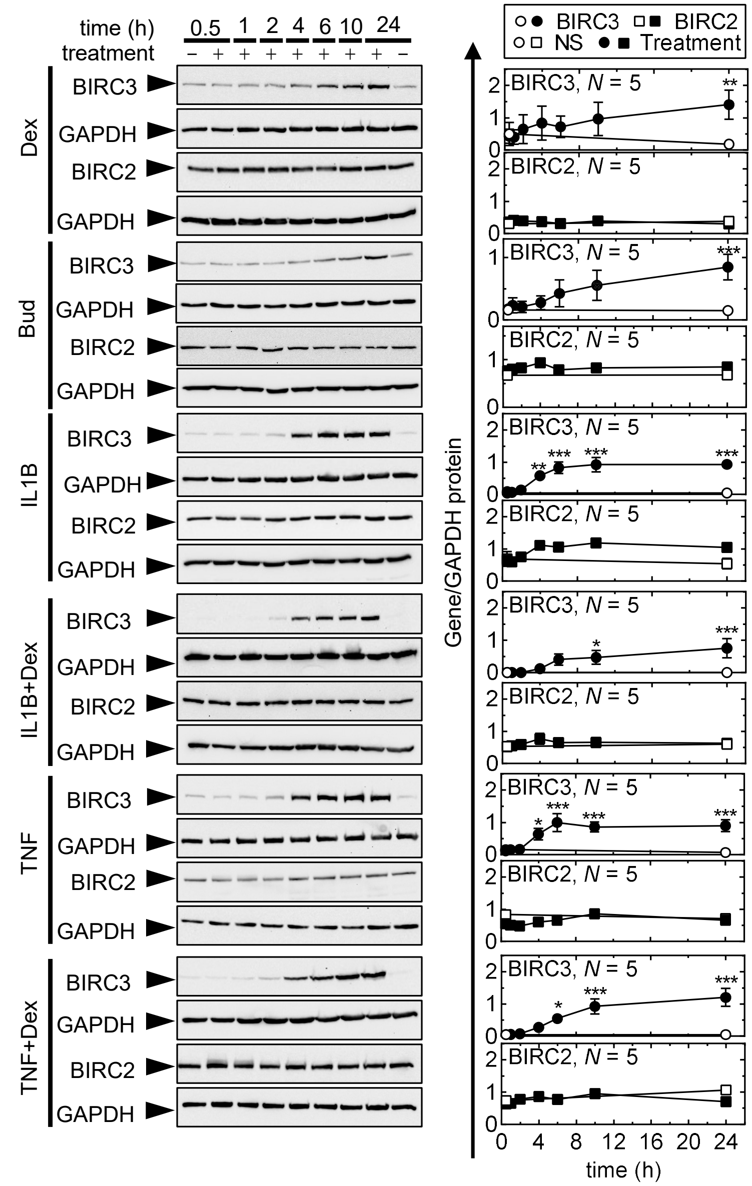

S3 Fig. Effects of inflammatory cytokines and glucocorticoids budesonide on BIRC2 and BIRC3 protein expression in A549 cells. A549 cells were not stimulated (NS) or treated with IL1B (1 ng/ml), TNF (10 ng/ml), and/or dexamethasone (*Dex*; 1 µM) or budesonide (*Bud*; 300 nM). Cells were harvested at the indicated times for western blot analysis and representative blots are shown. Data for BIRC3 and BIRC2 were normalized to GAPDH and plotted as mean ±SE. Significance relative to NS at ½ h was tested using one-way ANOVA with a Dunnett's post-hoc test. In each panel, data are from $N = 5$ experiments and * $p \leq 0.05$ and *** $p \leq 0.001$ indicates significance.

Supplemental Fig. S4

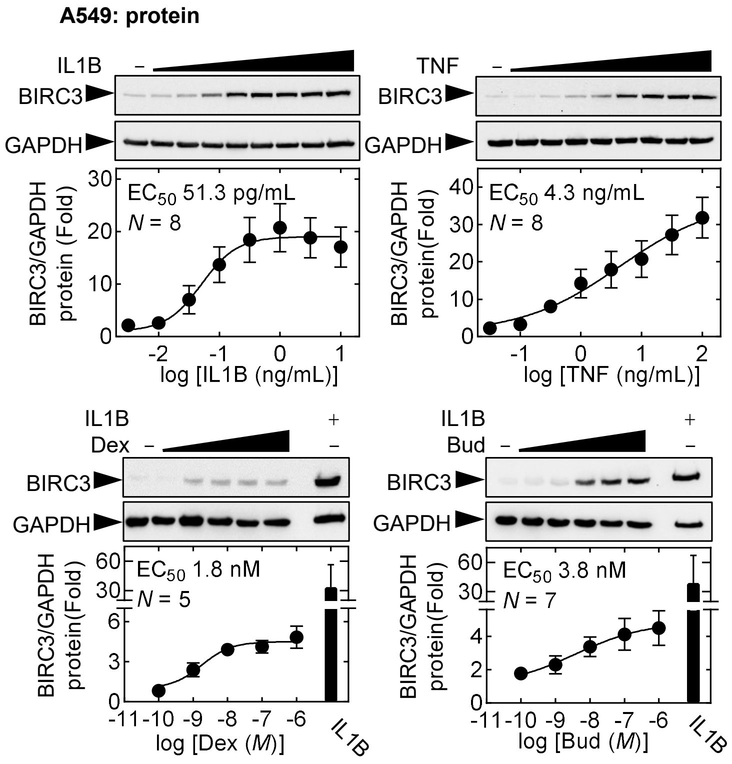

S4 Fig. Concentration-dependent effects of inflammatory cytokines and glucocorticoids on BIRC3 protein expression. A549 cells were either not stimulated or treated with the indicated concentrations of IL1B, TNF, dexamethasone (*Dex*), budesonide (*Bud*). (*lower panels*) IL1B at 1 ng/ml is shown as a comparator. Cells were harvested after 6 h for western blot analysis and representative blots are shown. Data for BIRC3 and BIRC2, from *N* = 5-8 experiments, were normalized to GAPDH and plotted as fold of untreated as mean ±SE.

Supplemental Fig. S5

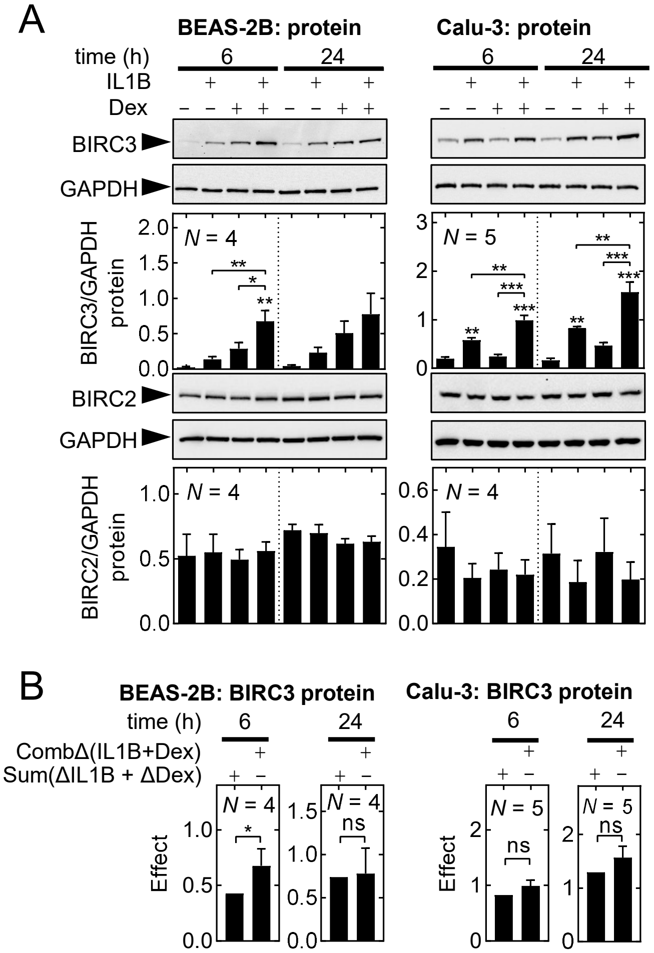

S5 Fig. Inflammatory cytokines and glucocorticoids differentially regulate BIRC2 and BIRC3 in pulmonary epithelial cells. (A) BEAS-2B cells or Calu-3 cells were not stimulated (NS) or treated with IL1B (1 ng/ml), and/or dexamethasone (*Dex*; 1 μM) or budesonide (*Bud*; 300 nM). Cells were harvested 6 or 24 h for western blot analysis and representative blots are shown. Data for BIRC3 and BIRC2 were normalized to GAPDH and plotted as mean ±SE. **(B)** The sum of the effects (*i.e.*, fold – 1) of IL1B and Bud (*sum(ΔIL1B + ΔBud)*) and the effect (fold – 1) of the combination treatment (*comboΔ(IL1B+Bud)*) from *A,* is plotted. All data are from $N = 4$-5 experiments and significance was tested: in *A,* against untreated, or as indicated, using one-way ANOVA with a Tukey's post-hoc test; and in *B,* by paired *t* test. * $p \leq 0.05$, ** $p \leq 0.01$, *** $p \leq 0.001$.

Supplemental Fig. S6

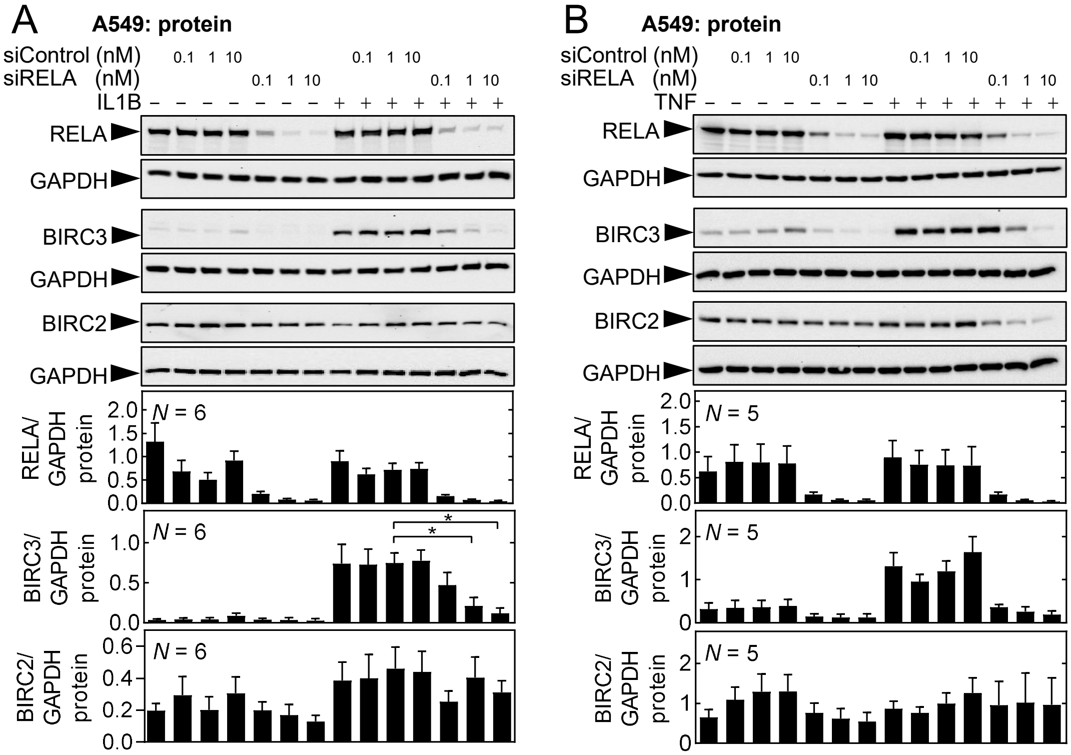

S6 Fig. RELA silencing reduces BIRC3 expression in a concentration-dependent manner. A549 cells were incubated with control siRNAs (siCTL) or RELA-targeting siRNAs (siRELA) at the indicated concentrations for 48 h. Following, cells were then not stimulated or treated with: (**A**) IL1B (1 ng/ml), or (**B**) TNF (10 ng/ml). After 6 h, cells were harvested for western blot analysis and representative blots are shown. Data from $N = 5$-6 experiments for RELA, BIRC3 and BIRC2 were normalized to GAPDH and plotted as mean ±SE. Significance was tested using one-way ANOVA with a Bonferroni's post-hoc test. * $p \leq 0.05$.

Supplemental Fig. S7

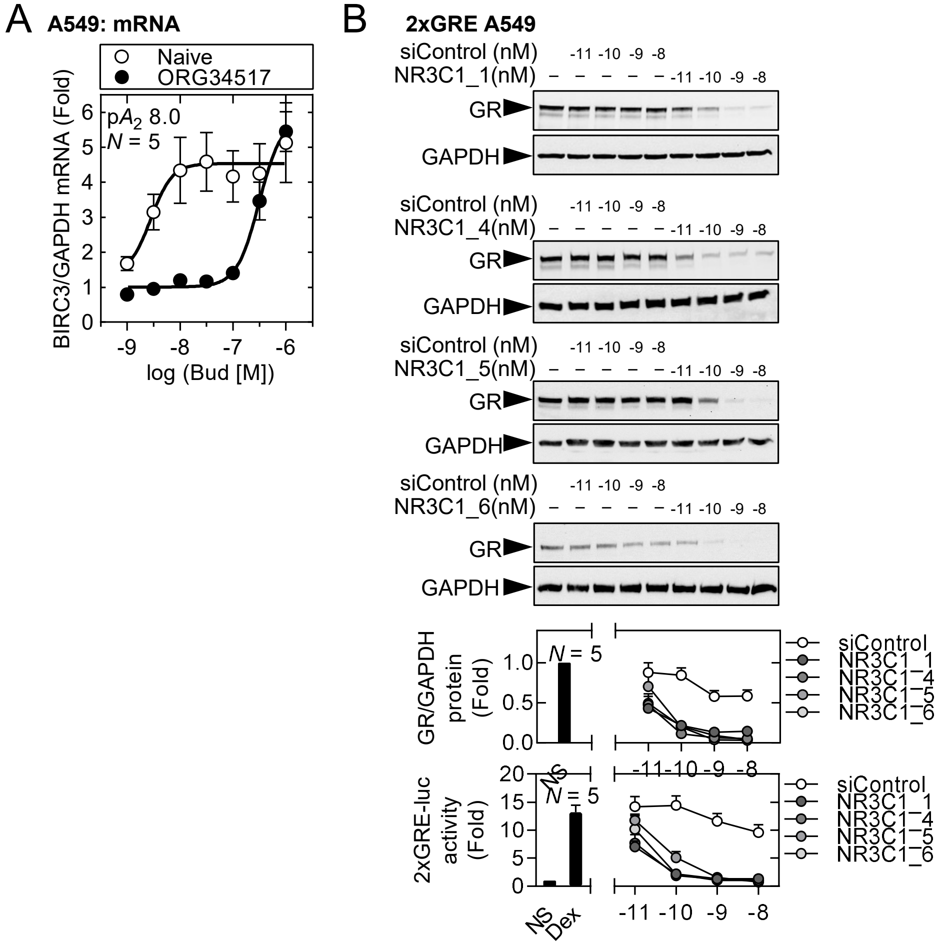

S7 Fig. Role of GR in glucocorticoid-induced transcription. (**A**) A549 cells were either not treated or pre-treated with ORG35417 (1 µM) for 1 h before addition of increasing concentrations of budesonide (*Bud*). Cells were harvested at 6 h for qPCR and Schild analysis. (**B**) 2×GRE A549 reporter cells were incubated with control siRNAs (siCTL) or individual GR-targeting siRNAs (NR3C1_1, NR3C1_4, NR3C1_5, and NR3C1_6) at the indicated concentrations for 48 h. After 6 h, cells were harvested for luciferase activity determination or western blot analysis and representative blots are shown. Data from N = 5 experiments were expressed as fold of NS and plotted as means ±SE.

Supplemental Fig. S8

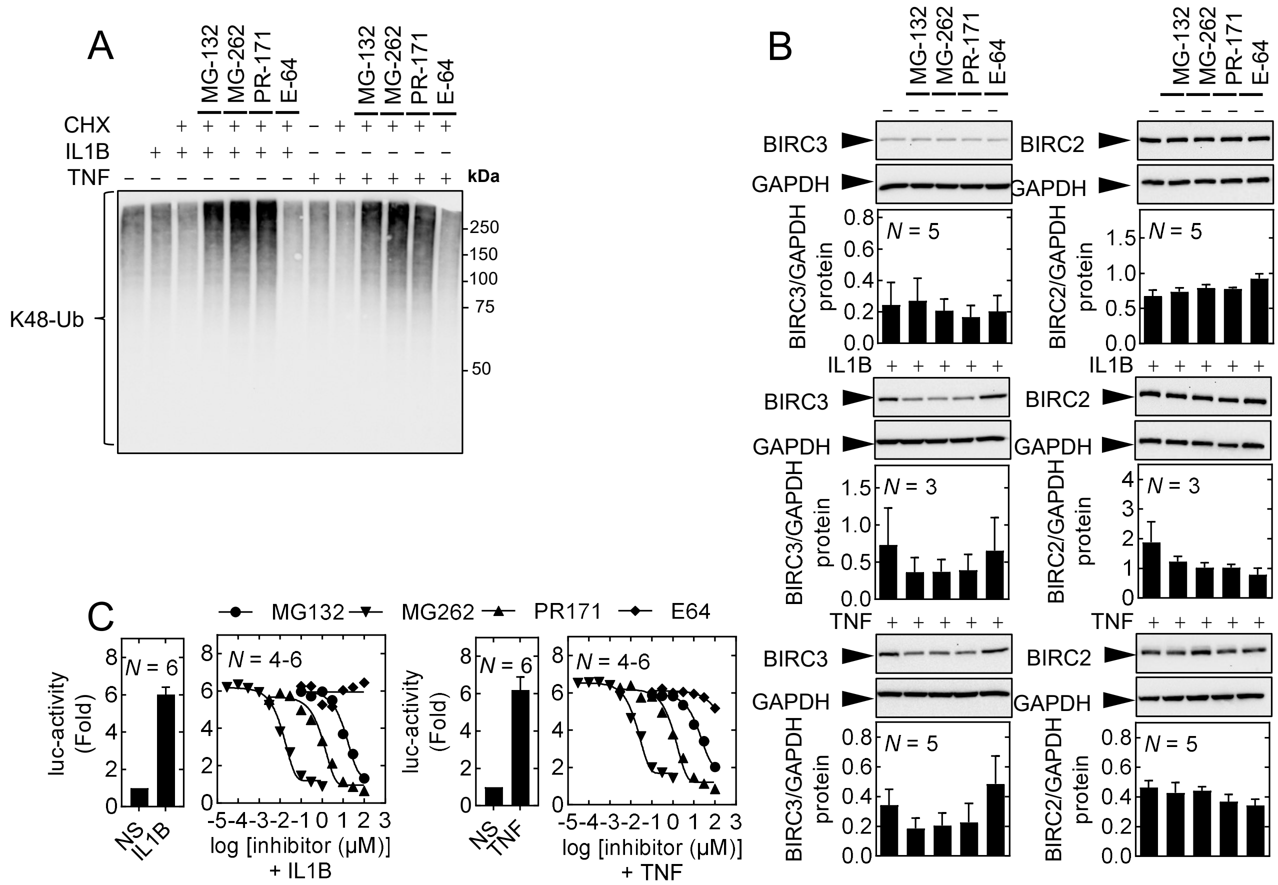

S8 Fig. Effect of proteasome inhibitors on protein expression and NF-κB activity. (A-B) A549 cells were either not stimulated (NS) or treated with IL1B (1 ng/ml), or TNF (10 ng/ml) for 1 h before the addition of MG-132 (10 μM), MG-262 (10 μM), PR-171 (10 μM) or E-64 (10 μM), either in the absence or presence of cycloheximide (CHX; 10 μg/ml). Cells were harvested after 6 h in *A,* and 2 h in *B,* for western blot analysis of K48-Ub, BIRC3, BIRC2 and GAPDH. Representative blots are shown. **(C)** A549 cells harbouring the NF-κB-dependent reporter, 6κBtkluc.neo, were not stimulated (NS) or treated with IL1B (1 ng/ml) or TNF (10 ng/ml) with or without the indicated concentrations of MG-132, MG-262, PR-171 or E-64. Cells were harvested after 6 h for luciferase activity determination. Data from *N* = 1-6 experiments for BIRC3 and BIRC2 were normalized to GAPDH in *A* and *B,* and expressed as fold of NS in *C,* plotted as means ±SE. Significance tested using one-way ANOVA with a Tukey's post-hoc test.
