# Supplementary material for: Differential regulation of BIRC2 and BIRC3 expression by inflammatory cytokines and glucocorticoids in pulmonary epithelial cells
Source: PLoS One. 2023 Jun 8;18(6):e0286783. doi: 10.1371/journal.pone.0286783 (PMC10249814; doi:10.1371/journal.pone.0286783)
Supplement: S2 File — Included are tabular data values used in each individual graph of (1) mRNA expression represented as GENE/GAPDH, (2) NF-κB-dependent luciferase activity shown as relative light units (RLU) of Treatment RLU / Non-stimulated RLU and (3) protein expression as raw densitometric values given as GENE/GAPDH. Additionally, the original, uncropped images for each representative blot are shown with molecular weight marker included. Any lanes not included in the final figure have been marked with an “X” above each lane. (PDF) [file pone.0286783.s003.pdf]

Supporting Information. S9 Fig.

Differential regulation of BIRC2 and BIRC3 expression by inflammatory cytokines and glucocorticoids in pulmonary epithelial cells

Andrew Thorne[1], Akanksha Bansal[1], Amandah Necker-Brown[1], Mahmoud Mostafa[1], Alex Gao[1], Andrei Georgescu[1], Cora Kooi[2], Richard Leigh[1,2] and Robert Newton[1,*]

[1] Department of Physiology & Pharmacology and Lung Health Research Group, Snyder Institute for Chronic Diseases, Cumming School of Medicine, and [2] Department of Medicine and Airways Inflammation Research Group, Snyder Institute for Chronic Diseases, University of Calgary, Calgary, Alberta, Canada

*Corresponding author

E-mail: rnewton@ucalgary.ca

Figure 1A - A549 cells: mRNA

		mRNA (Fold)			
		BIRC3/GAPDH			
Treatment	Time	N = 1	N = 2	N = 3	N = 4
NS	1	1	1	1	1
NS	2	1.381819	0.992206	1.062212	0.794003
NS	4	1.108128	1.033219	0.856097	0.855591
NS	6	1.339916	0.710345	0.894386	0.798502
NS	12	1.106194	0.697766	0.671605	0.660615
NS	18	0.909396	0.64237	0.761769	0.567704
IL1B	1	9.018907	6.580429	6.42688	6.640074
IL1B	2	62.08359	28.98488	21.3405	19.56895
IL1B	4	51.59331	56.25767	48.0072	43.50692
IL1B	6	28.58983	24.34689	18.88198	16.14614
IL1B	12	21.61518	15.79496	12.96813	8.839429
IL1B	18	27.9174	20.91433	11.78716	9.934658
Dex	1	3.309717	1.70397	1.641257	1.967526
Dex	2	3.525968	2.882783	4.435709	3.249061
Dex	4	5.71572	5.963708	4.155139	3.381145
Dex	6	3.716623	4.365228	4.200946	3.816477
Dex	12	5.154703	4.400218	3.245754	3.284766
Dex	18	5.012448	5.065374	1.934368	3.201144
IL+Dex	1	1.390673	7.002738	6.773177	9.077587
IL+Dex	2	45.53856	29.02472	17.29936	22.99595
IL+Dex	4	65.09337	28.79781	35.63577	37.2059
IL+Dex	6	23.26881	26.95309	15.98047	16.29147
IL+Dex	12	24.27942	17.03741	12.6717	11.34387
IL+Dex	18	27.24345	19.84722	10.90098	9.289716

		mRNA (Fold)			
		BIRC2/GAPDH			
Treatment	Time	N = 1	N = 2	N = 3	N = 4
NS	1	1	1	1	1
NS	2	1.212563	0.95694	0.936088	0.814531
NS	4	1.180779	0.989637	0.749171	1.08931
NS	6	1.627259	0.959381	0.924298	1.072957
NS	12	1.018453	0.858476	0.67938	0.714276
NS	18	1.08546	1.159184	0.938655	0.81568
IL1B	1	1.919557	1.530406	1.237521	1.469242
IL1B	2	5.11854	3.254244	2.911042	1.947257
IL1B	4	6.440597	5.567154	4.515834	4.265298
IL1B	6	4.754954	3.269135	2.315379	2.642418
IL1B	12	2.016214	1.950036	2.441762	1.446921
IL1B	18	2.370718	2.559848	1.411646	1.533779
Dex	1	1.226707	1.212906	1.005158	1.122241
Dex	2	0.875641	1.008768	1.172862	0.985884
Dex	4	1.406467	1.474795	1.016577	1.19739
Dex	6	1.24238	1.615693	1.740092	1.172492
Dex	12	0.987547	1.154582	0.877695	0.924177
Dex	18	1.138951	1.566978	0.732151	1.126683
IL+Dex	1	0.098653	1.419016	1.121786	1.420669
IL+Dex	2	3.189332	2.506974	1.824033	2.1537
IL+Dex	4	4.02563	2.627427	2.291466	2.861925
IL+Dex	6	2.40877	2.368222	1.00793	1.379643
IL+Dex	12	1.540485	1.274643	1.0531	1.037817
IL+Dex	18	1.492143	1.483647	1.262623	1.090453

Figure 1A - A549 cells: mRNA

		mRNA (Fold)				
		BIRC3/GAPDH				
Treatment	Time	N = 1	N = 2	N = 3	N = 4	N = 5
NS	0.5	1	1	1	1	1
NS	1	0.977937	1.146044	1.169402	1.259274	1.489264
NS	2	0.767674	1.279547	1.381448	1.42704	1.33102
NS	4	0.794431	1.627925	1.050918	1.264702	1.014262
NS	6	1.561025	1.438177	1.683899	1.824809	1.253706
NS	8	1.663976	1.344463	1.559524	1.976846	1.399393
NS	10	1.534131	2.023783	1.403193	1.284759	0.501209
NS	24	1.757881	3.407689	2.340004	2.156252	1.293705
TNF	0.5	0.937216	1.269717	1.130418	1.744135	1.858567
TNF	1	3.029102	1.8459	1.909591	1.594194	1.035037
TNF	2	20.985	15.098	10.49473	11.9807	16.00771
TNF	4	20.19791	18.12897	13.42754	19.19014	47.50956
TNF	6	25.23803	14.505	15.31099	14.90631	24.72998
TNF	8	23.59841	16.66322	12.89288	11.97793	15.58979
TNF	10	12.788	18.77857	12.16924	23.72415	23.52858
TNF	24	16.69462	11.27938	16.16743	14.46899	25.56734
Dex	0.5	1.037972	1.101265	1.360481	1.177385	1.221031
Dex	1	1.508116	1.127566	1.406404	1.426728	0.90892
Dex	2	2.936388	4.421182	2.341212	2.890576	4.078967
Dex	4	4.703712	7.560076	3.117709	3.567082	3.508549
Dex	6	8.14706	6.08684	4.243091	5.444904	4.700956
Dex	8	8.335202	7.923223	4.280949	4.71652	3.992817
Dex	10	6.116455	5.952757	3.678025	4.435911	3.953101
Dex	24	5.483819	3.511117	4.215365	5.556602	4.490736
TNF+Dex	0.5	1.084951	1.092177	1.421904	1.040205	1.035976
TNF+Dex	1	2.940056	1.712823	1.761468	2.105308	1.193028
TNF+Dex	2	13.93136	17.64801	9.446202	12.39364	18.35591
TNF+Dex	4	23.85249	26.37845	16.84877	23.58309	45.84354
TNF+Dex	6	35.37483	29.11027	44.73304	26.69705	42.73017
TNF+Dex	8	32.83378	29.93286	16.07186	27.09259	39.75407
TNF+Dex	10	30.93914	32.25507	20.5896	22.01274	38.0208
TNF+Dex	24	31.91882	21.84995	23.95059	24.7145	23.13406

		mRNA (Fold)				
		BIRC2/GAPDH				
Treatment	Time	N = 1	N = 2	N = 3	N = 4	N = 5
NS	0.5	1	1	1	1	1
NS	1	1.095487	1.352864	1.147255	1.035134	1.071041
NS	2	0.968063	1.229147	1.350593	0.960033	1.095555
NS	4	1.041688	1.545477	1.207142	1.097178	0.958864
NS	6	1.352594	1.454313	1.558645	1.338073	0.975409
NS	8	1.42547	1.395702	1.374816	1.186306	0.776313
NS	10	1.3057	2.301917	1.313413	1.120058	0.835328
NS	24	1.913161	2.134977	1.681551	1.515311	0.793912
TNF	0.5	1.109668	1.639331	1.28194	1.258892	1.183529
TNF	1	1.413161	1.565854	1.797921	1.358814	0.753038
TNF	2	3.219004	3.727482	2.92697	3.055937	2.162264
TNF	4	4.408026	3.949084	3.257108	4.736391	3.657846
TNF	6	4.618236	4.06893	4.776105	3.38866	2.500869
TNF	8	5.514631	4.019022	3.256107	3.013972	1.80546
TNF	10	3.931743	5.015584	3.331222	4.085239	2.063477
TNF	24	3.859336	3.031407	3.971474	3.043051	2.142059
Dex	0.5	1.164725	1.017479	1.229797	1.139995	0.707665
Dex	1	1.226994	1.083809	1.282879	1.270222	0.711198
Dex	2	1.222089	1.274134	0.913464	1.296047	0.785817
Dex	4	1.561075	1.353652	1.220905	1.184417	0.823922
Dex	6	1.978404	1.413606	1.397868	1.205994	0.762975
Dex	8	1.950397	2.268668	1.816177	1.307227	0.894277
Dex	10	1.948764	1.402885	1.574329	1.535907	0.835937
Dex	24	1.943398	1.520296	2.037918	1.64623	0.503971
TNF+Dex	0.5	1.148636	1.116773	1.228765	1.181209	0.670378
TNF+Dex	1	1.647365	1.32608	1.103291	1.428961	0.972905
TNF+Dex	2	2.324383	2.552845	1.445302	2.08336	1.555641
TNF+Dex	4	3.052892	3.271443	1.845916	2.629271	2.660204
TNF+Dex	6	3.389042	3.04101	2.334638	2.152157	1.948553
TNF+Dex	8	3.029519	3.068089	1.390191	1.789814	1.200473
TNF+Dex	10	2.796833	2.388053	1.77409	2.135072	1.39885
TNF+Dex	24	2.560584	1.985716	1.792479	1.92077	1.386869

Figure 1B - A549 cells: mRNA

	6h			24h	
	BIRC3/GAPDH			BIRC3/GAPDH	
	Sum (ΔTNF + ΔDex)	Comb Δ(TNF+Dex)		Sum (ΔTNF + ΔDex)	Comb Δ(TNF+Dex)
N = 1	49.5681	66.33466	N = 1	12.17288	15.02538
N = 2	14.32116	21.31984	N = 2	11.85505	16.60869
N = 3	8.875825	9.472929	N = 3	10.66991	15.83099
N = 4	22.91237	24.88675	N = 4	33.45711	40.21461
N = 5	35.64398	30.27363	N = 5	12.52542	23.73588
N = 6	19.72199	41.17368	N = 6	14.07641	12.34266
N = 7	21.38665	22.66128	N = 7	12.61658	18.15756
N = 8	14.31802	20.24109	N = 8	4.34033	6.411953
N = 9	11.61238	26.56516	N = 9	8.710575	10.23528
N = 10	11.15252	14.63005	N = 10	9.287221	11.46178
N = 11	23.47516	34.08309	N = 11	23.23409	17.88202

Figure 1C - pHBEC-SC: mRNA

	mRNA (Fold)				
	BIRC3/GAPDH				
	N = 1	N = 2	N = 3	N = 4	N = 5
NS	1	1	1	1	1
Bud	2.31778	3.00631	4.115142	1.221228	6.573967
IL1B	7.048317	1.708988	8.527812	5.474639	16.93013
IL1B+Bud	44.68838	25.16167	76.85478	28.65023	71.48207
	mRNA (Fold)				
	BIRC2/GAPDH				
	N = 1	N = 2	N = 3	N = 4	N = 5
NS	1	1	1	1	1
Bud	1.102613	1.180493	1.20202	0.959373	1.216996
IL1B	1.203332	0.877057	1.253399	1.18956	1.036763
IL1B+Bud	1.41566	1.362298	1.714353	1.356906	1.43359

	6h	
	BIRC3/GAPDH	
	Sum (ΔIL1B + ΔBud)	Comb Δ(IL1B +Bud)
N = 1	9.366097	44.68838
N = 2	4.715298	25.16167
N = 3	12.64295	76.85478
N = 4	6.695867	28.65023
N = 5	23.5041	71.48207

Figure 1D - pHBEC-ALI: mRNA

	mRNA (Fold)				
	BIRC3/GAPDH				
	N = 1	N = 2	N = 3	N = 4	N = 5
NS	1	1	1	1	1
Bud	2.359803	3.367404	5.221682	2.44513	3.20084
IL1B	5.295734	9.245905	12.71228	11.2355	10.67062
IL1B+Bud	17.23409	27.23187	50.56796	45.17422	33.60321
	mRNA (Fold)				
	BIRC2/GAPDH				
	N = 1	N = 2	N = 3	N = 4	N = 5
NS	1	1	1	1	1
Bud	1.225184	1.39912904	1.35362996	1.024246	1.231132
IL1B	1.12558	1.59000489	1.21715314	1.508221	1.170569
IL1B+Bud	1.172428	1.44479624	1.24872397	1.389719	1.227049

	BIRC3/GAPDH	
	Sum (ΔIL1B + ΔBud)	Comb Δ(IL1B +Bud)
N = 1	72.57773	144.2016
N = 2	21.73194	88.25255
N = 3	17.60058	92.80582
N = 4	33.82988	88.78642
N = 5	21.96527	133.7952

Figure 2A - A549 cells: protein

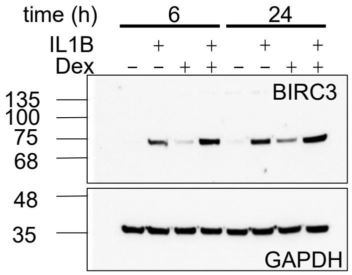

Chemiluminescence

			Adj. Total Band Vol.										
			BIRC3										
Time (h)	Stim	Lane	N = 1	N = 2	N = 3	N = 4	N = 5	N = 6	N = 7	N = 8	N = 9	N = 10	N = 11
6	NS	1	2548890	3291030	4620330	2867402	1741512	594236	13233780	5337766	5566768	882812	833612
	IL1B	2	22571900	21609369	34305699	10312252	3059268	6465788	109769270	49258469	49557404	3983068	4106560
	Dex	3	4512780	6631110	7453440	3252505	19013294	1478912	22825117	9062069	19465252	32915784	52341744
	IL1B + Dex	4	30934260	25541370	30544110	4154609	29116537	6563520	215488375	46567125	47646984	56931706	98064750
24	NS	5	2098350	3497580	1318044	488622	479333	1024172	7882432	3661800	5098308	934308	2968072
	IL1B	6	27990720	30719160	49120512	37707075	6919262	8570524	154418706	57634887	58959172	16643048	25523648
	Dex	7	8431921	10435282	7533656	4208292	29303085	3044108	69182477	18437320	18968972	59614820	95622332
	IL1B + Dex	8	46240380	39312331	65484158	60774629	55023912	9941608	328929472	113815410	116528171	136220812	215667090

			Adj. Total Band Vol.										
			GAPDH										
Time (h)	Stim	Lane	N = 1	N = 2	N = 3	N = 4	N = 5	N = 6	N = 7	N = 8	N = 9	N = 10	N = 11
6	NS	1	29526042	43413342	22244568	41531748	85107208	42817560	196204852	8227314	153124144	1.59E+08	1.24E+08
	IL1B	2	25529070	26604354	10987508	21151153	160997760	60051390	150033834	10258370	173995062	1.27E+08	1.02E+08
	Dex	3	32286086	35747918	15125916	34285936	135457200	60720276	143920440	10813531	178707942	99556080	66565850
	IL1B + Dex	4	31365762	24837021	10446177	14058720	130813200	50456526	129984396	10833295	166719007	1.01E+08	81041328
24	NS	5	28794303	26748576	10915560	10561536	105131183	72101826	134756380	14227152	176061328	79095666	71643528
	IL1B	6	20347038	16848650	23801456	23614864	124700688	63330684	134062530	11716087	144971868	70319730	60806673
	Dex	7	19881228	16453110	15403530	10143798	104584320	44709378	130649660	12686292	176773954	70520301	61453151
	IL1B + Dex	8	16929255	14914725	27463275	18627420	131750717	68238900	155174976	12402522	209602575	98670096	74317824

			Normalized Values										
			BIRC3										
Time (h)	Stim	Lane	N = 1	N = 2	N = 3	N = 4	N = 5	N = 6	N = 7	N = 8	N = 9	N = 10	N = 11
6	NS	1	0.086327	0.075807	0.207706	0.069041	0.020463	0.013878	0.067449	0.648786	0.036355	0.005541	0.006721
	IL1B	2	0.884165	0.812249	3.122246	0.48755	0.019002	0.107671	0.73163	4.801783	0.284821	0.031339	0.040453
	Dex	3	0.139775	0.185496	0.49276	0.094864	0.140364	0.024356	0.158595	0.838031	0.108922	0.330626	0.786315
	IL1B + Dex	4	0.986243	1.028359	2.923951	0.295518	0.222581	0.130083	1.657802	4.298519	0.285792	0.566136	1.210059
24	NS	5	0.072874	0.130758	0.120749	0.046264	0.004559	0.014205	0.058494	0.257381	0.028958	0.011812	0.041428
	IL1B	6	1.375666	1.823242	2.063761	1.596752	0.055487	0.13533	1.151841	4.919295	0.406694	0.236677	0.419751
	Dex	7	0.424115	0.634244	0.489086	0.414864	0.280186	0.068087	0.529527	1.453326	0.107306	0.845357	1.55602
	IL1B + Dex	8	2.731389	2.635807	2.384426	3.262643	0.417637	0.145688	2.119733	9.176796	0.555948	1.380568	2.901956

Figure 2A - A549 cells: protein

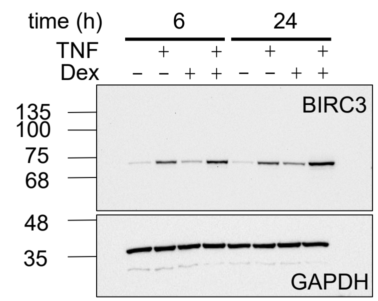

Chemiluminescence

			Adj. Total Band Vol.														
			BIRC3								GAPDH						
Time (h)	Stim	Lane	N = 1	N = 2	N = 3	N = 4	N = 5	N = 6	N = 7	Lane	N = 1	N = 2	N = 3	N = 4	N = 5	N = 6	N = 7
6	NS	1	2585412	1445828	4605390	2176110	1907288	882812	833612	1	258980172	157374778	192234510	271654736	238875608	159321408	124040000
	TNF	2	37050912	13058704	53432292	26169402	23674422	3983068	4106560	2	213972771	151866870	168052889	260329260	231562402	127095150	101514236
	Dex	3	5978016	7484952	13698288	4735980	3968562	15649864	30002132	3	174975164	168197764	170003736	260578236	220980308	68890120	76477282
	TNF + Dex	4	50154906	25437426	82553808	22152036	41023497	32077252	47876409	4	167837901	149107079	175219100	235828694	279893814	89509600	49968415
24	NS	5	3843840	3633806	4395378	1946449	1499229	934308	2968072	5	163267432	155200236	150323964	210133504	274230136	79095666	71643528
	TNF	6	50343606	34898400	56623986	29253267	33329646	16643048	25523648	6	204576330	155264490	157418380	179680752	273611596	70319730	60806673
	Dex	7	21000534	12365844	31972218	18999696	11930586	31362868	51108970	7	193718928	150082816	183580264	204305696	366689664	76217440	60600020
	TNF + Dex	8	157414320	86335100	187578612	156072125	141729068	218070288	250994664	8	238911039	165527226	174357490	183839920	296937963	126197600	87267680

			Normalized Values						
			BIRC3						
Time (h)	Stim	Lane	N = 1	N = 2	N = 3	N = 4	N = 5	N = 6	N = 7
6	NS	1	0.00998305	0.009187165	0.023957145	0.008010573	0.007984	0.005541	0.006721
	TNF	2	0.17315714	0.085987839	0.317949262	0.100524244	0.102238	0.031339	0.040453
	Dex	3	0.03416494	0.044500901	0.080576394	0.018174887	0.017959	0.227171	0.392301
	TNF + Dex	4	0.29882944	0.170598379	0.471146171	0.093932743	0.146568	0.358367	0.958133
24	NS	5	0.02354321	0.023413663	0.02923937	0.009262916	0.005467	0.011812	0.041428
	TNF	6	0.24608715	0.224767428	0.359703778	0.162806904	0.121814	0.236677	0.419751
	Dex	7	0.10840724	0.08239347	0.174159342	0.092996409	0.032536	0.411492	0.843382
	TNF + Dex	8	0.65888257	0.521576432	1.075827669	0.848956663	0.477302	1.728007	2.876147

Figure 2A - A549 cells: protein

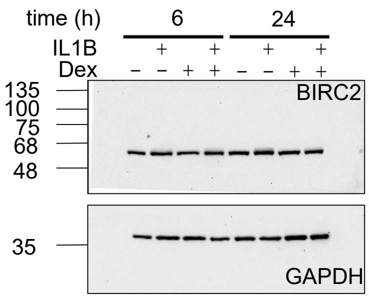

Chemiluminescence

			Adj. Total Band Vol.						Adj. Total Band Vol.				
			BIRC2						GAPDH				
Time (h)	Stim	Lane	N = 1	N = 2	N = 3	N = 4	N = 5	Lane	N = 1	N = 2	N = 3	N = 4	N = 5
6	NS	1	181069419	111282879	61347825	108523208	80555640	1	189162276	93019472	135862725	259754880	259465944
	IL1B	2	234086076	167189652	166415172	129672079	99124776	2	226707030	189366444	194376448	259935444	251472720
	Dex	3	162394243	121356186	115201317	98862828	81673446	3	227612892	177321390	135636752	306234308	300627936
	IL1B + Dex	4	194458305	146189093	103899642	70594386	77603096	4	213199029	96361616	132610968	230865584	323642156
24	NS	5	165990365	186408388	122036145	119989472	68216035	5	183933810	166652904	171080820	316914813	308986370
	IL1B	6	213971822	206275366	128891532	64793617	86746842	6	162787734	159236880	180536224	191840750	244779120
	Dex	7	142669162	215629151	160679211	90203380	41487684	7	205022367	271972200	279762424	244309380	214244744
	IL1B + Dex	8	161481621	157722267	100132725	105497946	52350416	8	182155950	221553280	127555334	297947848	248084670

			Normalized Values				
			BIRC2				
Time (h)	Stim	Lane	N = 1	N = 2	N = 3	N = 4	N = 5
6	NS	1	0.95721738	1.196339612	0.45154272	0.417790834	0.3104671
	IL1B	2	1.03254882	0.882889537	0.85614885	0.498862629	0.39417705
	Dex	3	0.71346681	0.684385488	0.84933704	0.322833939	0.27167617
	IL1B + Dex	4	0.91209752	1.517088433	0.78349207	0.305781333	0.23978056
24	NS	5	0.90244618	1.118542693	0.71332453	0.37861743	0.22077361
	IL1B	6	1.31442226	1.295399445	0.71393723	0.337746892	0.35438824
	Dex	7	0.69587121	0.792835264	0.57434165	0.369217834	0.19364622
	IL1B + Dex	8	0.88650204	0.711893171	0.78501402	0.35408192	0.21101834

Figure 2A - A549 cells: protein

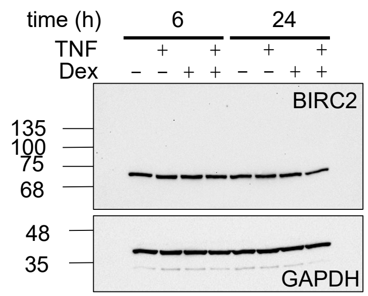

Chemiluminescence

			Adj. Total Band Vol.						Adj. Total Band Vol.				
			BIRC2						GAPDH				
Time (h)	Stim	Lane	N = 1	N = 2	N = 3	N = 4	N = 5	Lane	N = 1	N = 2	N = 3	N = 4	N = 5
6	NS	1	220517185	89877630	207707100	97140825	58349824	1	196229210	133084754	214583975	289313762	311825710
	TNF	2	171178392	76226704	196207001	64497150	53693409	2	164335296	135196212	209353344	264507882	259765546
	Dex	3	96621331	80045171	194754477	99344700	60165798	3	149247936	143218822	210816400	299820064	264663880
	TNF + Dex	4	111168494	68929902	171144421	54915525	38066259	4	172840420	145426798	180475155	257464672	263073676
24	NS	5	166355244	149338837	167787622	76624425	75926697	5	157991040	168926967	201463243	270743448	274778160
	TNF	6	193746710	130280051	147359868	78737400	71224923	6	176732755	156141756	214221008	266106284	268800648
	Dex	7	144879118	84775158	178013173	93935250	88342485	7	173896785	150802288	205056710	284165712	302143248
	TNF + Dex	8	111620385	64893605	105738250	57278250	35550200	8	192682572	156857346	202060896	325976420	370290696

			Normalized Values				
			BIRC2				
Time (h)	Stim	Lane	N = 1	N = 2	N = 3	N = 4	N = 5
6	NS	1	1.12377349	0.675341294	0.96795252	0.335762891	0.1871232
	IL1B	2	1.04164106	0.563822779	0.937205	0.243838291	0.2066995
	Dex	3	0.64738806	0.558901197	0.92381085	0.331347738	0.22732909
	IL1B + Dex	4	0.64318574	0.473983495	0.94829907	0.213293438	0.14469809
24	NS	5	1.052941	0.884043795	0.83284484	0.283014882	0.27631998
	IL1B	6	1.09626939	0.834370346	0.6878871	0.295887037	0.26497303
	Dex	7	0.83313281	0.562160953	0.86811679	0.33056504	0.2923861
	IL1B + Dex	8	0.57929674	0.413710971	0.52329893	0.175712863	0.09600619

Figure 2B - A549 cells: protein

	6h				24h	
	Sum (ΔTNF + ΔDex)	Comb Δ(TNF+Dex)			Sum (ΔTNF + ΔDex)	Comb Δ(TNF+Dex)
N = 1	0.207322	0.298829		N = 1	0.354494	0.658883
N = 2	0.130489	0.170598		N = 2	0.30716	0.521576
N = 3	0.398525	0.471146		N = 3	0.533863	1.075828
N = 4	0.118699	0.093933		N = 4	0.255803	0.848957
N = 5	0.120197	0.146568		N = 5	0.15435	0.477302
N = 6	0.25851	0.358367		N = 6	0.648169	1.728007
N = 7	0.432754	0.958133		N = 7	1.263133	2.876147

Figure 2C – pHBEC-SC: protein

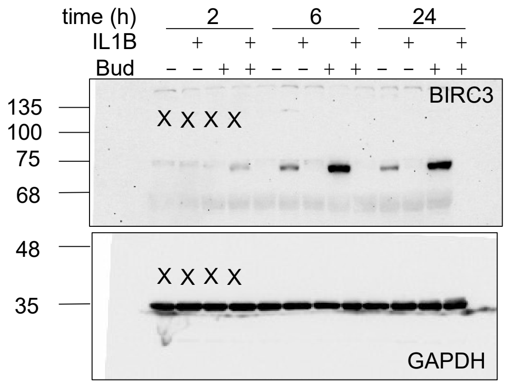

Chemiluminescence

			Adj. Total Band Vol.						Adj. Total Band Vol.				
			BIRC3						GAPDH				
Time (h)	Stim	Lane	N = 1	N = 2	N = 3	N = 4	N = 5	Lane	N = 1	N = 2	N = 3	N = 4	N = 5
2	NS	1	345243	118530	196695	3167010	1829520	1	11164800	90904576	1.17E+08	1.48E+08	1.16E+08
	IL1B	2	1209578	368685	252315	4907760	5684580	2	23058756	1.1E+08	1.21E+08	1.58E+08	1.2E+08
	Bud	3	818368	257445	114885	3273435	3515655	3	29476042	93073354	95614611	1.68E+08	1.17E+08
	IL1B + Bud	4	9781616	209115	4731480	15832245	29236350	4	34660322	96305952	1.27E+08	1.86E+08	1.42E+08
6	NS	5	529729	149850	84510	2640	3399825	5	32666946	1.06E+08	1.32E+08	1.42E+08	1.2E+08
	IL1B	6	2126978	107460	1252665	43093380	8684610	6	30781301	1.04E+08	1.42E+08	1.41E+08	1.28E+08
	Bud	7	659003	135675	147285	5101500	5517105	7	44641364	1.1E+08	1.47E+08	1.34E+08	1.14E+08
	IL1B + Bud	8	82641616	1527525	46221835	2.16E+08	1.18E+08	8	33949780	89478876	1.38E+08	1.42E+08	1.17E+08
24	NS	9	996908	224280	209790	325050	126555	9	21529800	94967840	1.2E+08	1.22E+08	1.29E+08
	IL1B	10	2154917	241920	294840	31422105	11654775	10	27890637	84777024	1.08E+08	1.11E+08	1.37E+08
	Bud	11	525976	65880	60480	1320018	5103582	11	38604704	91832136	1.29E+08	1.18E+08	1.1E+08
	IL1B + Bud	12	60006621	14867280	27679590	2.28E+08	1.85E+08	12	32818608	85304640	96381429	1.65E+08	1.12E+08

			Normalized Values				
			BIRC3				
Time (h)	Stim	Lane	N = 1	N = 2	N = 3	N = 4	N = 5
2	NS	1	0.0309225	0.001304	0.001682	0.021363722	0.015728
	IL1B	2	0.0524563	0.003337	0.002087	0.031105405	0.047187
	Bud	3	0.0277638	0.002766	0.001202	0.019488634	0.030013
	IL1B + Bud	4	0.2822137	0.002171	0.037157	8.521655E-02	0.205968
6	NS	5	0.0162161	0.001414	0.000642	1.85549E-05	0.028405
	IL1B	6	0.0690997	0.001032	0.00884	0.305231177	0.067699
	Bud	7	0.0147622	0.001229	0.001003	0.038151172	0.048608
	IL1B + Bud	8	2.4342313	0.017071	0.335795	1.519221963	1.008766
24	NS	9	0.0463036	0.002362	0.001742	0.002654729	0.000982
	IL1B	10	0.0772631	0.002854	0.002726	0.282360096	0.084967
	Bud	11	0.0136247	0.000717	0.000467	0.011213887	0.046305
	IL1B + Bud	12	1.8284329	0.174285	0.287188	1.380009343	1.650576

Figure 2C – pHBEC-SC: protein

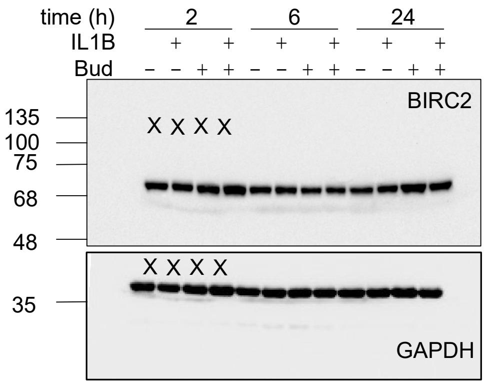

Chemiluminescence

			Adj. Total Band Vol.					Adj. Total Band Vol.			
			BIRC2					GAPDH			
Time (h)	Stim	Lane	N = 1	N = 2	N = 3	N = 4	Lane	N = 1	N = 2	N = 3	N = 4
2	NS	1	71612640	1.34E+08	2.78E+08	81702880	1	1.4E+08	1.26E+08	1.19E+08	1.26E+08
	IL1B	2	60386850	1.23E+08	2.34E+08	1.08E+08	2	1.12E+08	1.1E+08	94374261	1.5E+08
	Bud	3	80417070	1.32E+08	1.79E+08	84857490	3	1.2E+08	1.34E+08	96270262	1.31E+08
	IL1B + Bud	4	58556790	1.68E+08	1.22E+08	85186350	4	1.4E+08	1.65E+08	89980198	1.3E+08
6	NS	5	43106580	1.1E+08	1.21E+08	53373330	5	1.04E+08	1.28E+08	1.27E+08	1.18E+08
	IL1B	6	34258005	1.05E+08	1.2E+08	36393975	6	99438381	1.4E+08	1.28E+08	89326570
	Bud	7	52324785	79838794	1.14E+08	40660338	7	1.29E+08	1.43E+08	1.06E+08	78044439
	IL1B + Bud	8	57087450	85003930	1.05E+08	67258395	8	1.35E+08	1.33E+08	1.22E+08	1.3E+08
24	NS	9	76131450	90038016	1.34E+08	53286912	9	1.76E+08	1.64E+08	1.25E+08	1.18E+08
	IL1B	10	78577560	1.07E+08	1.89E+08	60816420	10	1.67E+08	1.59E+08	1.49E+08	1.29E+08
	Bud	11	58873500	1.87E+08	2E+08	87870825	11	1.56E+08	1.68E+08	1.34E+08	1.38E+08
	IL1B + Bud	12	79890534	1.34E+08	2.66E+08	95971266	12	1.8E+08	1.25E+08	1.48E+08	1.51E+08

			Normalized Values			
			BIRC2			
Time (h)	Stim	Lane	N = 1	N = 2	N = 3	N = 4
2	NS	1	1.0564106	0.572866	2.330481	0.646125401
	IL1B	2	1.1199387	0.43231	2.477943	0.723556017
	Bud	3	0.9879377	0.209509	1.862051	0.648874168
	IL1B + Bud	4	1.0190288	0.009635	1.352024	0.653842846
6	NS	5	0.8625572	0.505235	0.954745	0.452499887
	IL1B	6	0.7475071	0.011515	0.940863	0.407426088
	Bud	7	0.5594243	0.491554	1.07727	0.520989561
	IL1B + Bud	8	0.6377323	0.646647	0.861872	0.517895471
24	NS	9	0.5486105	0.784866	1.064474	0.451967023
	IL1B	10	0.6748595	0.003209	1.264479	0.470047261
	Bud	11	1.113804	0.000957	1.488422	0.635265741
	IL1B + Bud	12	1.0764704	0.436808	1.799426	0.634362313

Figure 2C – pHBEC-ALI: protein

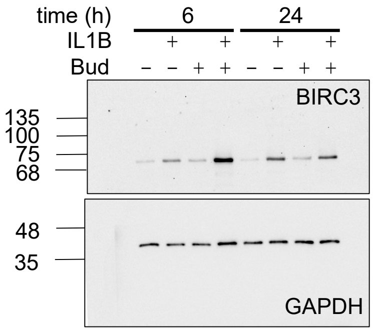

Chemiluminescence

			Adj. Total Band Vol.						Adj. Total Band Vol.				
			BIRC3						GAPDH				
Time (h)	Stim	Lane	N = 1	N = 2	N = 3	N = 4	N = 5	Lane	N = 1	N = 2	N = 3	N = 4	N = 5
6	NS	1	4541922	5847534	3885695	4360415	1914880	1	65546780	105500780	111832461	100836360	103059047
	IL1B	2	22700502	31975812	37062130	46413125	9390936	2	45928624	61240740	119426723	132562671	85580000
	Bud	3	9190764	12951576	5761355	9750465	2166752	3	51179056	60120060	104327860	95639709	43280166
	IL1B + Bud	4	191828538	68056758	53321935	81991540	19344368	4	114170002	31940700	108480424	116738388	32590885
24	NS	5	3091968	4176018	6875055	8250625	3533580	5	62416906	23041260	89624444	111187176	52844418
	IL1B	6	48519900	32477544	46535460	44142080	26771726	6	73776344	41411040	90486968	102127146	41543492
	Bud	7	10468260	7592706	18534935	35382765	10253546	7	105447758	125065820	119866136	133715706	37886180
	IL1B + Bud	8	48962430	77449878	110951395	208172890	83493792	8	94773778	152406100	119649764	143930020	70763411

			Adj. Total Band Vol.				
			Normalized Values				
Time (h)	Stim	Lane	N = 1	N = 2	N = 3	N = 4	N = 5
6	NS	1	0.069293	0.055426	0.034746	0.043242	0.01858
	IL1B	2	0.494256	0.522133	0.310334	0.350122	0.109733
	Bud	3	0.179581	0.215429	0.055224	0.10195	0.050063
	IL1B + Bud	4	1.680201	2.130722	0.491535	0.702353	0.593551
24	NS	5	0.049537	0.181241	0.07671	0.074205	0.066868
	IL1B	6	0.657662	0.784273	0.514278	0.432227	0.644426
	Bud	7	0.099274	0.06071	0.15463	0.264612	0.270641
	IL1B + Bud	8	0.516624	0.508181	0.927301	1.446348	1.179901

Figure 2C – pHBEC-ALI : protein

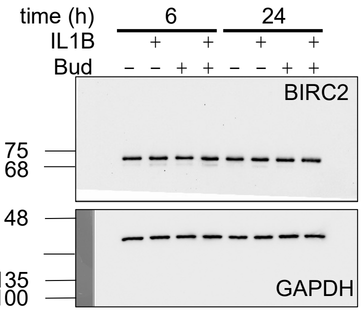

Chemiluminescence

			Adj. Total Band Vol.										
			BIRC2						GAPDH				
Time (h)	Stim	Lane	N = 1	N = 2	N = 3	N = 4	N = 5	Lane	N = 1	N = 2	N = 3	N = 4	N = 5
6	NS	1	74620000	108674994	115829376	59629758	153944980	1	65546780	105500780	111832461	100836360	135786768
	IL1B	2	59124416	120769332	128220976	80294817	157527230	2	45928624	61240740	119426723	132562671	149127238
	Bud	3	46474064	116992668	105591408	74616585	135811780	3	51179056	60120060	104327860	95639709	94252236
	IL1B + Bud	4	105189344	99344778	135310032	125435972	150475200	4	114170002	31940700	108480424	116738388	96772130
24	NS	5	32843408	58819122	113348560	186053246	90367000	5	62416906	23041260	89624444	111187176	111612631
	IL1B	6	128391120	80717202	130900224	123476142	108609220	6	73776344	41411040	90486968	102127146	104654394
	Bud	7	37629488	94644816	131762384	100008540	108351850	7	105447758	125065820	119866136	133715706	99539271
	IL1B + Bud	8	32496464	81889584	117031200	127705470	146577620	8	94773778	152406100	119649764	143930020	114621338

			Adj. Total Band Vol.				
			Normalized Values				
Time (h)	Stim	Lane	N = 1	N = 2	N = 3	N = 4	N = 5
6	NS	1	0.91535	1.395397	0.780516	0.613148	1.133726
	IL1B	2	0.652077	1.50021	0.810513	0.577786	1.056328
	Bud	3	0.413665	2.170757	0.741729	0.869203	1.44094
	IL1B + Bud	4	0.461849	1.055512	0.920844	1.014544	1.554944
24	NS	5	0.294994	0.60452	0.920907	1.379484	0.809649
	IL1B	6	0.780007	0.791978	0.942612	1.58925	1.037789
	Bud	7	0.306339	0.898268	0.79152	0.667545	1.088534
	IL1B + Bud	8	0.220427	0.546915	0.799663	0.754688	1.278799

Figure 2D - pHBEC-SC: protein

	6h				24h	
	Sum (ΔIL1B + ΔBud)	Comb Δ(IL1B +Bud)			Sum (ΔIL1B + ΔBud)	Comb Δ(IL1B +Bud)
N = 1	0.083862	2.434231		N = 1	0.108726	1.684328
N = 2	0.002261	0.017071		N = 2	0.090888	1.828433
N = 3	0.009843	0.335795		N = 3	0.003571	0.174285
N = 4	0.343382	1.519222		N = 4	0.003194	0.287188
N = 5	0.116307	1.008766		N = 5	0.293574	1.380009
				N = 6	0.131272	1.650576

Figure 2D - pHBEC-ALI cells: protein

	6h				24h	
	Sum (ΔIL1B + ΔBud)	Comb Δ(IL1B +Bud)			Sum (ΔIL1B + ΔBud)	Comb Δ(IL1B +Bud)
N = 1	0.159796	0.593551		N = 1	0.915067	1.179901
N = 2	0.673837	1.680201		N = 2	0.756936	0.516624
N = 3	0.737562	2.130722		N = 3	0.844983	0.508181
N = 4	0.365558	0.491535		N = 4	0.668908	0.927301
N = 5	0.452072	0.702353		N = 5	0.696839	1.446348

Figure 3A - A549 cells: NF-κB activity

			Fold / NS				
Treatment	siRNA	Concentration (nM)	N = 1	N = 2	N = 3	N = 4	N = 5
NS	-	-	1	1	1	1	1
IL1B	-	-	8.446834	5.930734	7.670668	8.156828	7.670668
IL1B	CTL	0.01	9.077589	6.278972	8.487441	7.6554	8.487441
IL1B	CTL	0.1	8.405603	5.576927	8.199057	7.964657	8.199057
IL1B	CTL	1	8.2871	5.314719	7.924501	7.579246	7.924501
IL1B	CTL	10	9.007249	6.250748	7.332627	7.660201	7.332627
IL1B	RELA	0.01	8.76228	6.33759	8.458401	8.229388	8.458401
IL1B	RELA	0.1	7.603877	5.576927	6.788605	7.322187	6.788605
IL1B	RELA	1	3.631615	3.197044	3.130222	3.285243	3.130222
IL1B	RELA	10	2.529518	2.034036	2.288794	2.164958	2.288794

Figure 3B - A549 cells: NF-κB activity

		Fold / NS			
Treatment	siRNA	N = 1	N = 2	N = 3	N = 4
NS	-	1.490574	1.623173	3.208311	1.643298
IL1B	-	6.28154	17.709	29.07408	12.11653
TNF	-	7.981885	15.66103	26.30586	7.8676
NS	CTL	1.036964	1.605157	2.303709	1.695467
IL1B	CTL	5.045008	17.62437	27.30032	16.46785
TNF	CTL	6.566144	16.01604	25.42992	10.90752
NS	RELA	0.638321	1.302166	2.441707	1.144594
IL1B	RELA	2.171879	7.026136	12.00507	3.950721
TNF	RELA	2.004077	7.64761	11.5442	5.511644

Figure 3C - A549 cells: protein

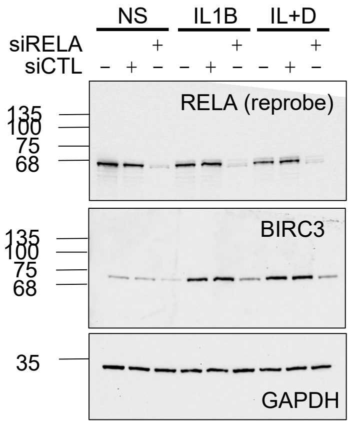

Chemiluminescence

			Adj. Total Band Vol.						Adj. Total Band Vol.						Adj. Total Band Vol.				
			RELA						BIRC3						GAPDH				
siRNA	Treatment	Lane	N = 1	N = 2	N = 3	N = 4	N = 5	Lane	N = 1	N = 2	N = 3	N = 4	N = 5	Lane	N = 1	N = 2	N = 3	N = 4	N = 5
-	NS	1	48238632	1.93E+08	98359370	1.2E+08	47674920	1	1705630	16891464	11797860	4434645	4630625	1	78693076	2.18E+08	1.39E+08	1.7E+08	85328772
CTL	NS	2	78938730	1.29E+08	1.07E+08	1.07E+08	50079440	2	4850400	13189998	14477619	5483855	7359766	2	1.41E+08	1.7E+08	1.8E+08	1.35E+08	83156160
RELA	NS	3	1243944	10784556	7928536	7796880	1825010	3	1106850	4282827	2855376	1150546	2895963	3	95060746	1.51E+08	1.52E+08	90605376	82969200
-	IL1B	4	56434716	1.19E+08	66369464	1.77E+08	71627060	4	28016935	91080522	74900295	1.21E+08	70919426	4	34659724	1.38E+08	1.07E+08	2.02E+08	95581020
CTL	IL1B	5	1.13E+08	1.33E+08	91231600	1.28E+08	53907825	5	79825740	1.17E+08	85035600	95295036	58615945	5	1.02E+08	1.27E+08	1.34E+08	1.84E+08	80591844
RELA	IL1B	6	2450916	9069724	5332864	11949266	3810055	6	8200325	27593199	16954032	38380054	23227693	6	82177392	1.84E+08	1.38E+08	2.04E+08	1.19E+08
-	IL + Dex	7	1.93E+08	77951168	60022780	1.35E+08	56723595	7	1.35E+08	1.29E+08	1.11E+08	1.76E+08	96637021	7	1.98E+08	1.16E+08	1.14E+08	1.96E+08	97444464
CTL	IL + Dex	8	55917342	1.13E+08	66904800	1.03E+08	71628940	8	33906270	1.48E+08	1.22E+08	1.16E+08	1.14E+08	8	69864674	1.48E+08	1.15E+08	2.07E+08	95367840
RELA	IL + Dex	9	3398148	7659648	3931328	9141342	6242305	9	9779525	23502579	19849698	44599312	36656147	9	78729618	1.3E+08	78333178	1.44E+08	1.31E+08

			Normalized Values						Normalized Values				
			RELA						BIRC3				
siRNA	Treatment	Lane	N = 1	N = 2	N = 3	N = 4	N = 5	Lane	N = 1	N = 2	N = 3	N = 4	N = 5
-	NS	1	0.612997	0.883514	0.706874	0.707906	0.55872	1	0.021674	0.077463	0.084787	0.026095	0.054268
CTL	NS	2	0.558078	0.758134	0.595072	0.792639	0.602234	2	0.034291	0.077736	0.080542	0.040583	0.088505
RELA	NS	3	0.013086	0.07155	0.05233	0.086053	0.021996	3	0.011644	0.028414	0.018846	0.012698	0.034904
-	IL1B	4	1.628251	0.865129	0.61785	0.874469	0.749386	4	0.808343	0.662133	0.697265	0.597976	0.741982
CTL	IL1B	5	1.106991	1.048157	0.680771	0.692795	0.668899	5	0.784921	0.921915	0.634537	0.516699	0.727319
RELA	IL1B	6	0.029825	0.049277	0.038653	0.058714	0.032003	6	0.099788	0.149918	0.122884	0.188585	0.195103
-	IL + Dex	7	0.974213	0.670001	0.526943	0.68863	0.582112	7	0.679774	1.105043	0.972918	0.89722	0.991714
CTL	IL + Dex	8	0.800366	0.761369	0.582674	0.497064	0.751081	8	0.485314	0.995694	1.066165	0.56131	1.193328
RELA	IL + Dex	9	0.043162	0.058777	0.050187	0.063295	0.047718	9	0.124217	0.18035	0.253401	0.308809	0.280212

Figure 3C - A549 cells: protein

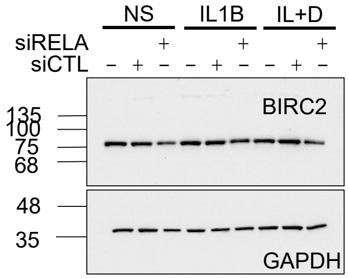

Chemiluminescence

			Adj. Total Band Vol.						Adj. Total Band Vol.				
			BIRC2						GAPDH				
siRNA	Treatment	Lane	N = 1	N = 2	N = 3	N = 4	N = 5	Lane	N = 1	N = 2	N = 3	N = 4	N = 5
-	NS	1	64505560	31949960	58631144	33254550	2.27E+08	1	1.59E+08	90135360	2.14E+08	1.65E+08	1.42E+08
CTL	NS	2	28640196	35020206	50204780	31552172	1.66E+08	2	79301224	81338792	1.72E+08	1.26E+08	1.49E+08
RELA	NS	3	17925336	29226600	34946292	30252612	76645919	3	89254456	93993736	1.73E+08	1.31E+08	83566812
-	IL1B	4	73143954	45205290	43686840	41279156	2.13E+08	4	1.49E+08	98601576	1.24E+08	96042115	1.08E+08
CTL	IL1B	5	25060698	44857332	52225352	60547412	1.7E+08	5	75220880	1.27E+08	84476400	66154650	1.06E+08
RELA	IL1B	6	16829982	26660556	33458052	34425832	1.15E+08	6	88522856	1.11E+08	97167495	65611755	1.18E+08
-	IL + Dex	7	35465274	20785284	40945256	51791388	1.66E+08	7	1.45E+08	74305512	1.41E+08	1.07E+08	1.51E+08
CTL	IL + Dex	8	8751600	24119316	59805836	43615396	2.59E+08	8	67780136	82988488	1.3E+08	1.16E+08	1.85E+08
RELA	IL + Dex	9	10719280	17818060	38402104	29040184	92220443	9	74726895	1.24E+08	1.98E+08	1.21E+08	1.6E+08

			Normalized Values				
			BIRC2				
siRNA	Treatment	Lane	N = 1	N = 2	N = 3	N = 4	N = 5
-	NS	1	0.406023	0.354466	0.27355	0.202013	1.590457
CTL	NS	2	0.361157	0.430547	0.291422	0.249699	1.112999
RELA	NS	3	0.200834	0.310942	0.202126	0.231308	0.917181
-	IL1B	4	0.492188	0.458464	0.351457	0.429803	1.974923
CTL	IL1B	5	0.333161	0.35323	0.618224	0.91524	1.608789
RELA	IL1B	6	0.19012	0.2403	0.344334	0.52469	0.977538
-	IL + Dex	7	0.244345	0.279727	0.290008	0.485275	1.103432
CTL	IL + Dex	8	0.129117	0.290634	0.460099	0.377264	1.400534
RELA	IL + Dex	9	0.143446	0.143165	0.19358	0.239113	0.576971

Figure 3D - A549 cells: mRNA

		BIRC3				
Treatment	Virus	N = 1	N = 2	N = 3	N = 4	N = 5
NS	-	0.03352	0.058268	0.03354	0.656422	0.033438
NS	Ad.GFP	0.07666	0.056903	0.235741	0.097377	0.06314
NS	Ad.IkBaΔN	0.049783	0.032779	0.065122	0.042547	0.042457
IL1B	-	1.106601	1.4583	1.28574	1.168685	1.375132
IL1B	Ad.GFP	1.264056	1.123791	1.33042	0.946548	0.583368
IL1B	Ad.IkBaΔN	0.107659	0.069028	0.08483	0.19324	0.060512
Dex	-	0.315922	0.32628	0.245728	0.292056	0.438052
Dex	Ad.GFP	0.283253	0.227759	0.314362	0.317811	1.018896
Dex	Ad.IkBaΔN	0.40403	0.266744	0.245316	0.390927	0.283427
IL1B+Dex	-	1.259947	2.036252	1.571298	1.64545	1.783097
IL1B+Dex	Ad.GFP	1.483558	1.256049	1.439789	0.84955	1.620671
IL1B+Dex	Ad.IkBaΔN	0.320032	0.535548	0.336012	0.348162	0.300882

		BIRC2				
Treatment	Virus	N = 1	N = 2	N = 3	N = 4	N = 5
NS	-	0.81	0.99	0.77	2.07	1.36
NS	Ad.GFP	0.68	0.71	0.71	1.55	0.67
NS	Ad.IkBaΔN	0.82	0.57	0.69	1.12	0.91
IL1B	-	2.23	2.58	2.4	2.38	3.86
IL1B	Ad.GFP	2.14	1.74	1.94	2.08	1.72
IL1B	Ad.IkBaΔN	0.91	0.86	0.96	1.52	0.8
Dex	-	0.91	1.37	1.15	2.09	1.81
Dex	Ad.GFP	0.73	0.79	0.95	1.88	3.11
Dex	Ad.IkBaΔN	1.03	0.83	0.87	1.41	1.2
IL1B+Dex	-	1.28	2.34	1.68	2.62	2.3
IL1B+Dex	Ad.GFP	1.31	1.46	1.54	1.62	1.4
IL1B+Dex	Ad.IkBaΔN	0.79	0.81	0.68	1.28	0.85

Figure 4A - A549 cells: mRNA

		BIRC3									
Treatment	Inhibitor	N = 1	N = 2	N = 3	N = 4	N = 5	N = 6	N = 7	N = 8	N = 9	N = 10
NS	-	0.043351	0.154605	0.020706	0.07788	0.076723	0.018711	0.030974	0.049707	0.029748	0.032598
NS	PS-1145	1.265924	1.813195	0.68184	2.408957	0.804079	0.489132	0.584093	0.751935	0.638192	1.841399
IL1B	-	0.244218	0.442614	0.095893	0.400666	0.096181	0.081473	0.107074	0.161601	0.362404	0.567662
IL1B	PS-1145	1.447089	1.807303	0.673338	1.906142	0.737704	0.510481	0.331234	0.473475	0.486433	0.921824
Dex	-	0.015738	0.039921	0.019544	0.038558	0.023419	0.015573	0.01834	0.02591	0.017109	0.024914
Dex	PS-1145	0.299295	0.906503	0.378764	0.802526	0.469907	0.344945	0.296418	0.501671	0.219734	0.605774
IL1B+Dex	-	0.094752	0.260175	0.101946	0.213081	0.107498	0.063617	0.091957	0.103832	0.062787	0.101018
IL1B+Dex	PS-1145	0.576176	0.98321	0.406143	0.806112	0.329883	0.513725	0.18445	0.212949	0.16196	0.696476

		BIRC2									
Treatment	Inhibitor	N = 1	N = 2	N = 3	N = 4	N = 5	N = 6	N = 7	N = 8	N = 9	N = 10
NS	-	0.393031	0.368773	1.06636	0.594265	0.168766	0.353767	0.116369	0.564813	0.698706	0.180165
NS	PS-1145	1.608718	1.449545	1.732921	0.801284	0.597922	0.873953	1.086191	2.076901	2.620562	2.075869
IL1B	-	0.551733	0.458229	1.049089	0.313035	0.234169	0.475436	0.194695	0.118178	1.53585	1.27133
IL1B	PS-1145	1.101362	1.094781	1.117238	0.468132	0.318044	0.551773	0.716688	5.163978	2.767267	0.598385
Dex	-	0.387305	0.559796	0.962454	0.477001	0.266585	0.411143	0.133436	0.556223	0.631897	0.327548
Dex	PS-1145	1.366248	1.435643	1.667191	1.081625	0.7052	0.993688	1.182034	3.408342	3.364279	2.113481
IL1B+Dex	-	0.551266	0.690625	1.334531	0.585797	0.345589	0.475436	0.467954	0.963338	0.705448	0.318857
IL1B+Dex	PS-1145	1.211047	1.377174	1.545802	0.590276	0.541372	0.546866	1.215847	4.289018	4.402267	1.054228

Figure 4A - A549 cells: mRNA

		BIRC3				
Treatment	Inhibitor	N = 1	N = 2	N = 3	N = 4	N = 5
NS	-	0.030974	0.049707	0.029748	0.032598	0.04848
NS	PS-1445	0.01834	0.02591	0.017109	0.024914	0.039255
TNF	-	0.725337	0.978988	0.904802	0.511305	0.71388
TNF	PS-1445	0.216183	0.326562	0.281364	0.09756	0.311537
Dex	-	0.107074	0.161601	0.362404	0.567662	0.121519
Dex	PS-1445	0.091957	0.103832	0.062787	0.101018	0.074799
TNF+Dex	-	0.419036	1.153777	0.381943	0.86121	0.840253
TNF+Dex	PS-1445	0.173274	0.345203	0.294559	0.470747	0.185425
		BIRC2				
Treatment	Inhibitor	N = 1	N = 2	N = 3	N = 4	N = 5
NS	-	0.116369	0.564813	0.698706	0.180165	0.290518
NS	PS-1445	0.133436	0.556223	0.631897	0.327548	0.339939
TNF	-	1.138778	1.62993	1.430883	1.843276	0.679748
TNF	PS-1445	0.751885	1.311073	2.449548	0.587413	0.990101
Dex	-	0.194695	0.118178	1.53585	1.27133	0.252094
Dex	PS-1445	0.467954	0.963338	0.705448	0.318857	0.541463
TNF+Dex	-	0.45677	1.328135	1.301375	0.743918	0.696112
TNF+Dex	PS-1445	0.78679	3.303668	2.178562	1.171525	1.282315

Figure 4B - A549 cells: protein

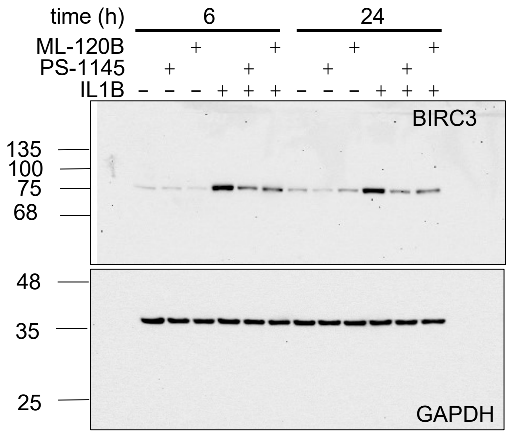

Chemiluminescence

				Adj. Total Band Vol.										Adj. Total Band Vol.								
				BIRC3										GAPDH								
Time (h)	Stim	Inhibitor	Lane	N = 1	N = 2	N = 3	N = 4	N = 5	N = 6	N = 7	N = 8	N = 9	Lane	N = 1	N = 2	N = 3	N = 4	N = 5	N = 6	N = 7	N = 8	N = 9
6	NS	-	1	4116728	6886688	15146838	14366225	1490681	8934760	2359441	230996	12278649	1	1.05E+08	1.41E+08	193018020	5.69E+08	30247668	1.97E+08	1.31E+08	1.89E+08	194497847
	NS	PS1145	2	4227592	4629064	7142451	10106910	1846750	331004	1812123	543840	8970637	2	91467531	1.02E+08	122833784	4.5E+08	87741312	1.26E+08	1.24E+08	1.84E+08	160850596
	NS	ML120B	3	2983816	2985456	9527798	9563426	1890906	1480056	871412	151300	10620994	3	99274203	93496356	84316386	4.37E+08	82017408	1.08E+08	1.19E+08	1.68E+08	148840977
	IL1B	-	4	1.26E+08	84739948	75048464	90472324	1.23E+08	1.31E+08	39168476	60681477	138992441	4	1.04E+08	1.09E+08	159245484	4.35E+08	76163456	87125376	1.25E+08	1.87E+08	160238828
	IL1B	PS1145	5	30102692	19720016	21590131	47629550	74866332	49665208	9620442	6151250	61693366	5	1.01E+08	1.03E+08	112400539	4.04E+08	82550528	1.08E+08	1.04E+08	1.7E+08	148047616
	IL1B	ML120B	6	40261672	46498100	52356252	59798684	1.39E+08	88887190	2892349	8937544	81570617	6	97488540	98580368	115278870	3.89E+08	78046720	1.06E+08	1.04E+08	1.93E+08	147704358
24	NS	-	7	181548	4084748	8065440	8897640	1595758	2824988	228726	109395	15925554	7	83685251	1.13E+08	73033008	3.2E+08	53952512	1.13E+08	89537574	1.73E+08	127332109
	NS	PS1145	8	81782	1358740	4056360	5609620	1699342	1743830	450116	262405	9666921	8	44330080	1.07E+08	116938232	2.41E+08	80232255	86449140	1E+08	1.56E+08	116390402
	NS	ML120B	9	113300	3386804	8973525	7339984	1641076	1361034	1362456	85371	10094465	9	62899840	1.08E+08	99544884	2.85E+08	89533312	83485056	1.04E+08	1.58E+08	123790121
	IL1B	-	10	20269724	81368600	69890535	1.04E+08	1.75E+08	2.37E+08	67782282	1.47E+08	100894912	10	53236480	1.04E+08	69723944	2.73E+08	87224704	79730944	1.09E+08	1.93E+08	121189995
	IL1B	PS1145	11	1624413	21652920	28420755	50093144	72622344	45402992	12212344	10272640	53735378	11	71724000	96600608	131901189	3.11E+08	1.18E+08	78334620	1.08E+08	1.47E+08	132916572
	IL1B	ML120B	12	4994779	35466920	39443910	57990072	1.55E+08	1.34E+08	36622092	10906962	82309139	12	82210880	87115683	85253104	2.87E+08	1.83E+08	1.91E+08	1.26E+08	1.74E+08	152169871

				Normalized Values								
				BIRC3								
Time (h)	Stim	Inhibitor	Lane	N = 1	N = 2	N = 3	N = 4	N = 5	N = 6	N = 7	N = 8	N = 9
6	NS	-	1	0.03925784	0.0487519	0.078473699	0.025236486	0.049283	0.045465	0.017958	0.001219	0.06313
	NS	PS1145	2	0.04621959	0.04560461	0.058147285	0.02247191	0.021048	0.002636	0.01464	0.002953	0.05577
	NS	ML120B	3	0.03005631	0.03193126	0.11300055	0.021890025	0.023055	0.013738	0.007293	0.000903	0.071358
	IL1B	-	4	1.21293686	0.78046061	0.471275305	0.208103992	1.61388	1.504966	0.314457	0.324112	0.867408
	IL1B	PS1145	5	0.29804497	0.19105686	0.192082095	0.118004193	0.906915	0.46193	0.092631	0.036138	0.416713
	IL1B	ML120B	6	0.41298877	0.47167708	0.454170413	0.15361375	1.779212	0.841508	0.027693	0.046335	0.552256
24	NS	-	7	0.00216941	0.03622007	0.110435544	0.027784478	0.029577	0.025103	0.002555	0.000633	0.125071
	NS	PS1145	8	0.00184484	0.01275023	0.034688057	0.023324099	0.02118	0.020172	0.004487	0.001684	0.083056
	NS	ML120B	9	0.00180128	0.0314137	0.090145517	0.025728721	0.018329	0.016303	0.013097	0.000541	0.081545
	IL1B	-	10	0.38074876	0.77980569	1.002389294	0.38052973	2.00608	2.971583	0.624002	0.761208	0.832535
	IL1B	PS1145	11	0.02264811	0.2241489	0.215470044	0.160993698	0.61412	0.579603	0.113052	0.069719	0.404279
	IL1B	ML120B	12	0.06075569	0.4071244	0.462668315	0.201738709	0.8422	0.700229	0.290429	0.062524	0.540903

Figure 4B - A549 cells: protein

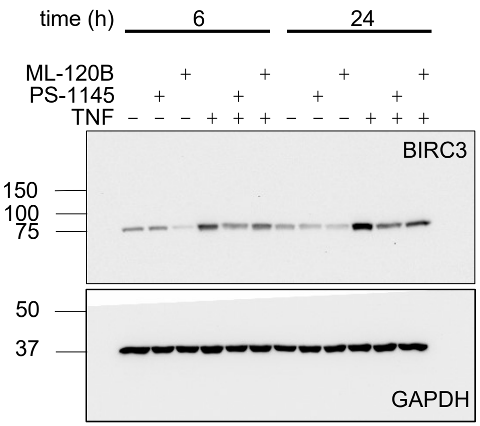

Chemiluminescence

				Adj. Total Band Vol.									Adj. Total Band Vol.							
				BIRC3									GAPDH							
Time (h)	Stim	Inhibitor	Lane	N = 1	N = 2	N = 3	N = 4	N = 5	N = 6	N = 7	N = 8	Lane	N = 1	N = 2	N = 3	N = 4	N = 5	N = 6	N = 7	N = 8
6	NS	-	1	7394842	358448	441012	11798720	36070749	42250825	28910366	7043211	1	44113470	1.07E+08	1.05E+08	272745204	2.35E+08	92400840	1.32E+08	1.98E+08
	NS	PS1145	2	1314600	702620	54483	8325744	20360340	20103570	26082700	8019925	2	39159359	84861983	94288880	199591096	2.36E+08	64306614	1.14E+08	2.08E+08
	NS	ML120B	3	474264	247078	71929	8129500	26437268	14968076	7697760	5087115	3	42418670	82295045	87037720	199513119	2.83E+08	71469120	1.03E+08	1.6E+08
	TNF	-	4	42725088	15965986	10054187	50818960	1.07E+08	1.18E+08	89032656	28622391	4	51354036	92805601	78021363	213860170	2.45E+08	70281060	1.17E+08	1.69E+08
	TNF	PS1145	5	1734264	4679002	1343056	8117288	24019281	76526867	51274431	6252372	5	52950480	83311140	75055176	173503176	2.51E+08	75100346	1.75E+08	1.63E+08
	TNF	ML120B	6	7240800	6910616	3169166	15019056	29916887	97803875	58553040	11765703	6	51289728	85526728	93842268	179672795	1.89E+08	65240890	1.26E+08	1.79E+08
24	NS	-	7	451752	559258	402831	4180338	21392085	42147441	29248820	3452556	7	44421504	97480040	1.07E+08	156931992	2.62E+08	84668980	1.35E+08	1.7E+08
	NS	PS1145	8	241416	585660	212069	4096984	13948792	29482734	21106503	3974232	8	48891626	87578407	98513220	173259645	2.31E+08	76691148	1.45E+08	2.09E+08
	NS	ML120B	9	71162	579812	48334	5216370	17027153	31410386	17509014	5073441	9	53783597	93652762	1E+08	164173296	2.62E+08	90826801	1.23E+08	1.97E+08
	TNF	-	10	19929168	10186098	81525920	38432868	1.47E+08	2.06E+08	1.74E+08	27622254	10	48541570	93310637	1.24E+08	197316525	2.43E+08	89884340	1.26E+08	2.13E+08
	TNF	PS1145	11	1956864	2644328	4314453	9894134	56593251	83988664	67493293	8204271	11	41774714	86069844	1.1E+08	206210758	1.89E+08	94972800	1.4E+08	2.34E+08
	TNF	ML120B	12	16736316	14525744	10470402	18180450	70012228	1.97E+08	96304316	12612341	12	43327284	1.07E+08	1.33E+08	269336616	2.32E+08	1.18E+08	1.25E+08	2.52E+08

				Normalized Values							
				BIRC3							
Time (h)	Stim	Inhibitor	Lane	N = 1	N = 2	N = 3	N = 4	N = 5	N = 6	N = 7	N = 8
6	NS	-	1	0.167632	0.003357	0.0042091	0.04325913	0.153264231	0.457255854	0.218966	0.035623
	NS	PS1145	2	0.033571	0.00828	0.00057783	0.04171401	0.086136649	0.312620565	0.229472	0.038599
	NS	ML120B	3	0.011181	0.003002	0.00082641	0.04074669	0.093437501	0.209434172	0.074959	0.031708
	TNF	-	4	0.831971	0.172037	0.12886454	0.23762704	0.434040842	1.685859846	0.761071	0.169301
	TNF	PS1145	5	0.032753	0.056163	0.01789425	0.04678467	0.095577824	1.018994866	0.293578	0.038262
	TNF	ML120B	6	0.141174	0.080801	0.0337712	0.08359115	0.158170729	1.499119264	0.463243	0.065697
24	NS	-	7	0.01017	0.005737	0.00375183	0.0266379	0.081628094	0.49779082	0.215947	0.020369
	NS	PS1145	8	0.004938	0.006687	0.0021527	0.0236465	0.060269227	0.384434642	0.145863	0.019012
	NS	ML120B	9	0.001323	0.006191	0.00048172	0.03177356	0.064907325	0.345827285	0.142279	0.025748
	TNF	-	10	0.410559	0.109163	0.6590356	0.19477775	0.60539375	2.294947129	1.385599	0.129626
	TNF	PS1145	11	0.046843	0.030723	0.03935143	0.04798069	0.299650482	0.884344402	0.483097	0.034993
	TNF	ML120B	12	0.386277	0.135572	0.07867971	0.06750085	0.301315681	1.663610795	0.76937	0.05007

Figure 4B - A549 cells: protein

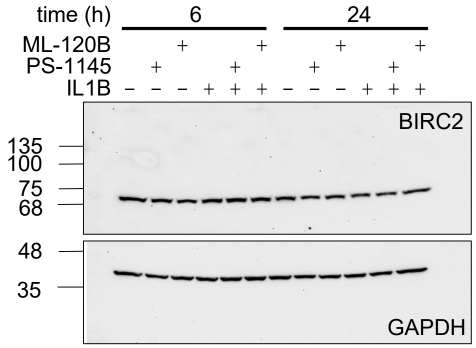

Chemiluminescence

				Adj. Total Band Vol.					Adj. Total Band Vol.			
				BIRC2					GAPDH			
Time (h)	Stim	Inhibitor	Lane	N = 1	N = 2	N = 3	N = 4	Lane	N = 1	N = 2	N = 3	N = 4
6	NS	-	1	1.55E+08	97207835	67424328	1.55E+08	1	2.88E+08	2.45E+08	1.24E+08	154595088
	NS	PS1145	2	80820728	72887928	42007580	92136808	2	2.11E+08	1.89E+08	73072259	97025666
	NS	ML120B	3	35716988	71266588	56675712	73668480	3	1.44E+08	1.7E+08	59444100	115657560
	IL1B	-	4	80123204	92665612	62333752	91657732	4	1.57E+08	1.5E+08	55364634	115554114
	IL1B	PS1145	5	73150184	71240836	54067804	1.16E+08	5	1.74E+08	1.59E+08	57515094	124992231
	IL1B	ML120B	6	66046924	71306548	39569428	97006600	6	1.74E+08	1.77E+08	50516928	127983744
24	NS	-	7	54505292	43211560	27876096	76525916	7	1.63E+08	1.2E+08	55199655	132287139
	NS	PS1145	8	57252320	28229816	8557212	54412644	8	1.47E+08	1.44E+08	23670612	125296769
	NS	ML120B	9	36878640	37908276	27145124	59280068	9	1.51E+08	1.58E+08	38779893	121675905
	IL1B	-	10	55413716	86036988	45462492	50105252	10	1.54E+08	1.58E+08	39565306	137715214
	IL1B	PS1145	11	88433848	444	45660664	47981304	11	1.5E+08	48717375	60885734	124748078
	IL1B	ML120B	12	89532275	1.67E+08	69735400	69818507	12	2.47E+08	2.28E+08	2.43E+08	235047418

				Normalized Values			
				BIRC2			
Time (h)	Stim	Inhibitor	Lane	N = 1	N = 2	N = 3	N = 4
6	NS	-	1	0.536247	0.397486	0.544396	1.002505655
	NS	PS1145	2	0.382815	0.385694	0.574877	0.949612734
	NS	ML120B	3	0.247493	0.419125	0.953429	0.636953434
	IL1B	-	4	0.510189	0.617263	1.125877	0.793201807
	IL1B	PS1145	5	0.419885	0.447161	0.940063	0.927848356
	IL1B	ML120B	6	0.379729	0.402018	0.78329	0.757960323
24	NS	-	7	0.335399	0.358655	0.505005	0.578483416
	NS	PS1145	8	0.390467	0.196208	0.361512	0.434270129
	NS	ML120B	9	0.243878	0.240501	0.699979	0.487196442
	IL1B	-	10	0.360711	0.544618	1.149049	0.363832365
	IL1B	PS1145	11	0.591068	9.11E-06	0.74994	0.384625597
	IL1B	ML120B	12	0.362861	0.733086	0.287115	0.297040093

Figure 4B - A549 cells: protein

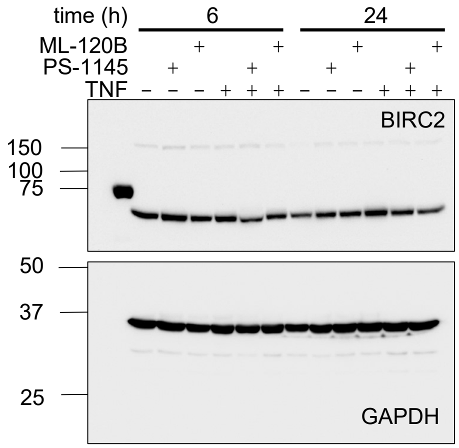

Chemiluminescence

				Adj. Total Band Vol.							
				BIRC2							
Time (h)	Stim	Inhibitor	Lane	N = 1	N = 2	N = 3	N = 4	N = 5	N = 6	N = 7	N = 8
6	NS	-	1	7394842	358448	441012	11798720	36070749	42250825	28910366	7043211
	NS	PS1145	2	1314600	702620	54483	8325744	20360340	20103570	26082700	8019925
	NS	ML120B	3	474264	247078	71929	8129500	26437268	14968076	7697760	5087115
	TNF	-	4	42725088	15965986	10054187	50818960	1.07E+08	1.18E+08	89032656	28622391
	TNF	PS1145	5	1734264	4679002	1343056	8117288	24019281	76526867	51274431	6252372
	TNF	ML120B	6	7240800	6910616	3169166	15019056	29916887	97803875	58553040	11765703
24	NS	-	7	451752	559258	402831	4180338	21392085	42147441	29248820	3452556
	NS	PS1145	8	241416	585660	212069	4096984	13948792	29482734	21106503	3974232
	NS	ML120B	9	71162	579812	48334	5216370	17027153	31410386	17509014	5073441
	TNF	-	10	19929168	10186098	81525920	38432868	1.47E+08	2.06E+08	1.74E+08	27622254
	TNF	PS1145	11	1956864	2644328	4314453	9894134	56593251	83988664	67493293	8204271
	TNF	ML120B	12	16736316	14525744	10470402	18180450	70012228	1.97E+08	96304316	12612341

	Adj. Total Band Vol.							
	GAPDH							
Time (h)	N = 1	N = 2	N = 3	N = 4	N = 5	N = 6	N = 7	N = 8
6	44113470	1.07E+08	1.05E+08	272745204	2.35E+08	92400840	1.32E+08	1.98E+08
	39159359	84861983	94288880	199591096	2.36E+08	64306614	1.14E+08	2.08E+08
	42418670	82295045	87037720	199513119	2.83E+08	71469120	1.03E+08	1.6E+08
	51354036	92805601	78021363	213860170	2.45E+08	70281060	1.17E+08	1.69E+08
	52950480	83311140	75055176	173503176	2.51E+08	75100346	1.75E+08	1.63E+08
	51289728	85526728	93842268	179672795	1.89E+08	65240890	1.26E+08	1.79E+08
24	44421504	97480040	1.07E+08	156931992	2.62E+08	84668980	1.35E+08	1.7E+08
	48891626	87578407	98513220	173259645	2.31E+08	76691148	1.45E+08	2.09E+08
	53783597	93652762	1E+08	164173296	2.62E+08	90826801	1.23E+08	1.97E+08
	48541570	93310637	1.24E+08	197316525	2.43E+08	89884340	1.26E+08	2.13E+08
	41774714	86069844	1.1E+08	206210758	1.89E+08	94972800	1.4E+08	2.34E+08
	43327284	1.07E+08	1.33E+08	269336616	2.32E+08	1.18E+08	1.25E+08	2.52E+08

				Normalized Values							
				BIRC2							
Time (h)	Stim	Inhibitor	Lane	N = 1	N = 2	N = 3	N = 4	N = 5	N = 6	N = 7	N = 8
6	NS	-	1	0.167632	0.003357	0.0042091	0.04325913	0.153264231	0.457255854	0.218966	0.035623
	NS	PS1145	2	0.033571	0.00828	0.00057783	0.04171401	0.086136649	0.312620565	0.229472	0.038599
	NS	ML120B	3	0.011181	0.003002	0.00082641	0.04074669	0.093437501	0.209434172	0.074959	0.031708
	TNF	-	4	0.831971	0.172037	0.12886454	0.23762704	0.434040842	1.685859846	0.761071	0.169301
	TNF	PS1145	5	0.032753	0.056163	0.01789425	0.04678467	0.095577824	1.018994866	0.293578	0.038262
	TNF	ML120B	6	0.141174	0.080801	0.0337712	0.08359115	0.158170729	1.499119264	0.463243	0.065697
24	NS	-	7	0.01017	0.005737	0.00375183	0.0266379	0.081628094	0.49779082	0.215947	0.020369
	NS	PS1145	8	0.004938	0.006687	0.0021527	0.0236465	0.060269227	0.384434642	0.145863	0.019012
	NS	ML120B	9	0.001323	0.006191	0.00048172	0.03177356	0.064907325	0.345827285	0.142279	0.025748
	TNF	-	10	0.410559	0.109163	0.6590356	0.19477775	0.60539375	2.294947129	1.385599	0.129626
	TNF	PS1145	11	0.046843	0.030723	0.03935143	0.04798069	0.299650482	0.884344402	0.483097	0.034993
	TNF	ML120B	12	0.386277	0.135572	0.07867971	0.06750085	0.301315681	1.663610795	0.76937	0.05007

Figure 5A- A549 cells: mRNA

		BIRC3					
Organon34517	Log [Dex]	N = 1	N = 2	N = 3	N = 4	N = 5	N = 6
-	-9	1.280101	1.011738	1.155542	1.066559	3.284264	1.184981
-	-8.5	3.380185	2.390472	1.424297	1.631947	3.204748	1.915415
-	-8	3.67451	2.974497	3.585009	3.093544	6.546978	2.921155
-	-7.5	3.487602	4.350597	4.648211	2.367499		3.799678
-	-7	2.352447	4.162297	5.950159	2.81225		3.317611
-	-6.5	3.139345	3.513252	7.578913	2.955248	6.185971	3.168424
-	-6	3.688092	3.125959	4.114685	2.970361	5.006248	4.195223
+	-9	1.346043	1.023169	1.236361	0.822474	1.407923	1.088602
+	-8.5	1.393432	0.952224	1.201878	1.298403	0.785587	1.165704
+	-8	1.53897	0.881275	1.191945	0.877856	1.131349	1.388167
+	-7.5	1.76606	1.040084	1.532347	0.863056	2.140604	1.277418
+	-7	2.414039	1.504536	1.49866	1.054101		1.172672
+	-6.5	2.72641	1.989286	2.486156	1.674859		2.049229
+	-6	4.070362	3.226751	5.419695	2.655978	2.817612	4.098327

Figure 5B - A549 cells: protein

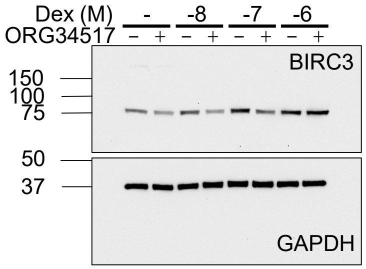

Chemiluminescence

				Adj. Total Band Vol.									Adj. Total Band Vol.							
				BIRC3									GAPDH							
Treatment	Concentration (M)	ORG34517	Lane	N = 1	N = 2	N = 3	N = 4	N = 5	N = 6	N = 7	N = 8	Lane	N = 1	N = 2	N = 3	N = 4	N = 5	N = 6	N = 7	N = 8
NS	-	-	1	18522877	54146171	11885837	22905012	10976868	64486278	41299104	79395475	1	2.95E+08	133005490	93772932	1.73E+08	1.88E+08	2.65E+08	2.56E+08	2.92E+08
NS	-	+	2	7656987	71728351	20394638	25037328	5859000	55392036	39655405	51126795	2	3.17E+08	202990180	93033160	2.05E+08	1.13E+08	2.14E+08	2.66E+08	2.52E+08
Dex	-8	-	3	59836352	122852745	38941089	43182632	9877896	75237530	81247650	94771910	3	3.45E+08	246447510	74229947	1.87E+08	79786176	2.04E+08	2.96E+08	2.6E+08
Dex		+	4	19198725	69393924	38433219	21592048	5704776	45199290	44036050	44868965	4	3.93E+08	261545480	1.44E+08	2.14E+08	58382400	1.95E+08	3.24E+08	2.8E+08
Dex	-7	-	5	98069891	116341793	44985369	48716552	8907696	113072126	1.6E+08	1.53E+08	5	3.62E+08	261282130	1.11E+08	2.42E+08	73713408	2.98E+08	3.64E+08	3.4E+08
Dex		+	6	29592275	91308972	49010291	27473912	9591120	132895598	99679405	86655140	6	4.05E+08	355421740	1.85E+08	1.35E+08	62921088	4.03E+08	3.41E+08	3.72E+08
Dex	-6	-	7	51258225	104062693	46365187	43990760	15259608	120333420	1.74E+08	1.69E+08	7	2.76E+08	291191820	88429138	1.32E+08	1.12E+08	2.73E+08	3.24E+08	3.75E+08
Dex		+	8	27077003	81622929	39411339	32812388	13061664	91880481	1.93E+08	2.22E+08	8	2.41E+08	291402500	80536480	1.21E+08	92754240	2.97E+08	3.35E+08	3.53E+08

				Normalized Values							
				BIRC3							
Stim	Concentration (M)	ORG34517	Lane	N = 1	N = 2	N = 3	N = 4	N = 5	N = 6	N = 7	N = 8
NS	-	-	1	0.06269	0.407097	0.126751	0.132501	0.0585	0.243639	0.161354	0.271715
NS	-	+	2	0.024168	0.353359	0.219219	0.12243	0.051685	0.259149	0.148816	0.202822
Dex	-8	-	3	0.173384	0.498495	0.524601	0.231013	0.123805	0.368602	0.274842	0.364845
Dex		+	4	0.04879	0.265323	0.267265	0.101074	0.097714	0.231743	0.135954	0.160041
Dex	-7	-	5	0.270733	0.445273	0.406617	0.200906	0.120842	0.379323	0.441085	0.449367
Dex		+	6	0.073006	0.256903	0.264929	0.203	0.152431	0.330161	0.292309	0.233134
Dex	-6	-	7	0.185612	0.357368	0.52432	0.333718	0.136745	0.441409	0.536779	0.449961
Dex		+	8	0.112453	0.280104	0.48936	0.271246	0.14082	0.309037	0.577349	0.627158

Figure 5C - A549 cells: mRNA

		BIRC3					
Treatment	siRNA	N = 1	N = 2	N = 3	N = 4	N = 5	N = 6
NS	-	0.035097	0.013312	0.015823	0.028804	0.013736	0.019403
NS	CTL	0.064729	0.02344	0.023912	0.050889	0.029122	0.05747
NS	GR	0.032896	0.012297	0.009645	0.017562	0.008874	0.026432
Dex	-	0.132357	0.065232	0.067433	0.117927	0.090877	0.088201
Dex	CTL	0.185358	0.056125	0.104173	0.113427	0.04891	0.130434
Dex	GR	0.031944	0.014953	0.031698	0.024563	0.011402	0.005559

Figure 5D - A549 cells: protein

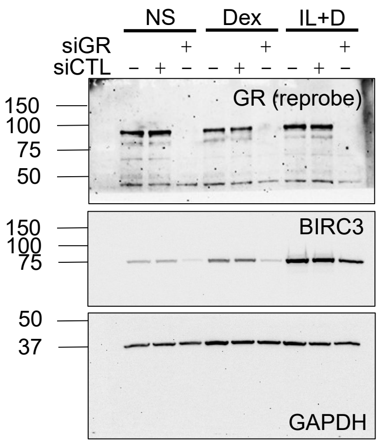

Chemiluminescence

			Adj. Total Band Vol.									Adj. Total Band Vol.							
			BIRC3									GAPDH							
siRNA	Treatment	Lane	N = 1	N = 2	N = 3	N = 4	N = 5	N = 6	N = 7	N = 8	Lane	N = 1	N = 2	N = 3	N = 4	N = 5	N = 6	N = 7	N = 8
-	NS	1	9531730	11432087	11228160	1740528	11155014	14060358	13333572	14790084	1	1.12E+08	1.37E+08	1.56E+08	77461404	1.21E+08	96886753	2.66E+08	1.79E+08
CTL	NS	2	7254143	10277478	14195760	2468232	12498876	20443644	13561344	15274932	2	76813440	1.61E+08	1.41E+08	1.02E+08	1.43E+08	1.1E+08	2.69E+08	1.86E+08
GR	NS	3	863265	2379962	759120	236088	4512924	15806700	7629336	2581194	3	41218560	1.61E+08	1.38E+08	30838140	1.2E+08	1.24E+08	2.35E+08	1.78E+08
-	Dex	4	33517406	23479838	24330000	7522632	16257618	27963936	34235352	44021712	4	2.57E+08	1.72E+08	1.51E+08	71760036	1.27E+08	1.12E+08	1.96E+08	1.79E+08
CTL	Dex	5	11975054	27282567	14176320	6923880	20554794	7559136	39663864	44782950	5	1.22E+08	1.89E+08	99378096	84527124	1.62E+08	97313440	2.35E+08	1.58E+08
GR	Dex	6	2279810	3191606	2392560	2810808	6252012	14442012	8022024	6875340	6	1.02E+08	1.93E+08	1.53E+08	78736836	1.3E+08	94288048	2.25E+08	1.57E+08
-	IL + Dex	7	1.9E+08	185927421	2.14E+08	83352240	1.89E+08	2.66E+08	1.97E+08	2.37E+08	7	2.13E+08	1.76E+08	1.61E+08	1.13E+08	1.43E+08	1.03E+08	2.01E+08	1.67E+08
CTL	IL + Dex	8	51014639	165393736	1.85E+08	68111712	2.11E+08	2.38E+08	2.77E+08	2.28E+08	8	1.07E+08	1.5E+08	1.17E+08	83206776	1.63E+08	1.05E+08	2.33E+08	1.06E+08
GR	IL + Dex	9	31712577	2833584	28157280	26567352	90847963	2.06E+08	85646120	1.1E+08	9	46109280	1.07E+08	84187350	51785868	94694268	94213603	2.32E+08	1.01E+08

			Normalized Values							
			BIRC3							
siRNA	Treatment	Lane	N = 1	N = 2	N = 3	N = 4	N = 5	N = 6	N = 7	N = 8
-	NS	1	0.084958	0.083477	0.072052	0.02247	0.092379	0.145122	0.050216	0.082669
CTL	NS	2	0.094438	0.06367	0.100933	0.024244	0.087223	0.186187	0.050341	0.081966
GR	NS	3	0.020944	0.014797	0.005502	0.007656	0.037616	0.127693	0.032528	0.014535
-	Dex	4	0.13057	0.136268	0.16074	0.10483	0.128104	0.249188	0.174936	0.245424
CTL	Dex	5	0.097862	0.144157	0.14265	0.081913	0.12722	0.077678	0.169081	0.283056
GR	Dex	6	0.02241	0.016547	0.015649	0.035699	0.048146	0.153169	0.035588	0.043767
-	IL + Dex	7	0.892186	1.058662	1.329006	0.738811	1.32264	2.59254	0.976594	1.415961
CTL	IL + Dex	8	0.476412	1.10082	1.579343	0.818584	1.299531	2.258322	1.187877	2.146777
GR	IL + Dex	9	0.68777	0.026372	0.33446	0.513023	0.959382	2.188198	0.369312	1.087763

Figure 5D - A549 cells: protein

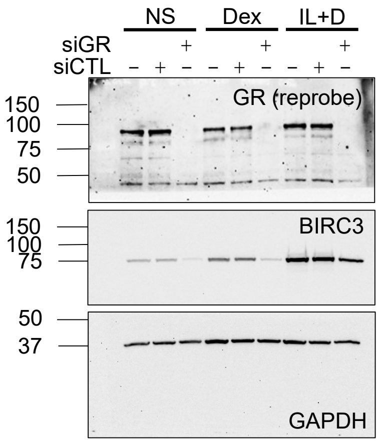

Chemiluminescence

			Adj. Total Band Vol.								Adj. Total Band Vol.						
			GR								GAPDH						
siRNA	Treatment	Lane	N = 1	N = 2	N = 3	N = 4	N = 5	N = 6	N = 7	Lane	N = 1	N = 2	N = 3	N = 4	N = 5	N = 6	N = 7
-	NS	1	11692044	60662948	1.05E+08	1.48E+08	2.71E+08	1.47E+08	2.32E+08	1	1.1E+08	1.23E+08	96695280	1.83E+08	3.09E+08	1.78E+08	1.56E+08
CTL	NS	2	3945594	47448936	77688720	1.49E+08	2.16E+08	1.41E+08	2.15E+08	2	35371088	1.02E+08	86724112	1.7E+08	2.95E+08	2.25E+08	1.41E+08
GR	NS	3	505938	42120	158760	2404302	52540985	26398028	6583	3	6802707	68678036	62794188	81020484	2.41E+08	1.45E+08	1.38E+08
-	Dex	4	9343470	16128350	18828515	62494308	1.27E+08	60027199	1.1E+08	4	69421496	99692788	46661836	1.11E+08	2.19E+08	1.14E+08	1.51E+08
CTL	Dex	5	1443252	14617438	16638646	44354844	1.5E+08	51408128	60145920	5	10746431	1.02E+08	59391800	80397360	2.5E+08	1.9E+08	99378096
GR	Dex	6	138804	66528	60912	1216470	17114496	13985712	172293	6	6850425	83572096	78523104	77513844	2.14E+08	1.79E+08	1.53E+08
-	IL + Dex	7	4623618	55763424	19002600	65905584	94924025	89316451	1.09E+08	7	36377263	2.08E+08	79381492	1.31E+08	2.01E+08	1.64E+08	1.61E+08
CTL	IL + Dex	8	254388	23892624	28504656	95534562	83824300	75263232	84785635	8	17824119	1.78E+08	78663872	1.96E+08	2.34E+08	1.68E+08	1.17E+08
GR	IL + Dex	9	46698	138240	24408	173634	15099105	15591680	12031	9	18693647	1.06E+08	51482928	1.13E+08	2.52E+08	2.22E+08	84187350

			Normalized Values						
			GR						
siRNA	Treatment	Lane	N = 1	N = 2	N = 3	N = 4	N = 5	N = 6	N = 7
-	NS	1	0.105831	0.491567	1.086684	0.807159	0.877717	0.827146	1.488789
CTL	NS	2	0.111549	0.466924	0.895815	0.877931	0.733042	0.627473	1.527198
GR	NS	3	0.074373	0.000613	0.002528	0.029675	0.217758	0.182033	4.77E-05
-	Dex	4	0.13459	0.161781	0.40351	0.563603	0.582536	0.525325	0.726709
CTL	Dex	5	0.134301	0.14337	0.280151	0.551695	0.600146	0.270883	0.605223
GR	Dex	6	0.020262	0.000796	0.000776	0.015694	0.080012	0.078256	0.001127
-	IL + Dex	7	0.127102	0.268144	0.239383	0.504056	0.47321	0.544641	0.674696
CTL	IL + Dex	8	0.014272	0.134037	0.36236	0.488293	0.358929	0.447323	0.722273
GR	IL + Dex	9	0.002498	0.001307	0.000474	0.001539	0.059913	0.070264	0.000143

Figure 6A - A549 cells: protein

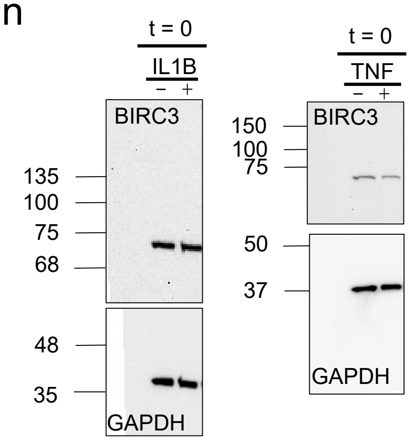

Chemiluminescence

		Adj. Total Band Vol.						Adj. Total Band Vol.				
		BIRC3						GAPDH				
Treatment	Lane	N = 1	N = 2	N = 3	N = 4	N = 5	Lane	N = 1	N = 2	N = 3	N = 4	N = 5
NS	1	8082029	27874890	66974156	66893556	33772800	1	197587221	1.63E+08	221078484	169078166	261322200
IL1B	2	6363346	52169389	81751130	53212125	26587125	2	145292895	50766884	227865835	163780636	210224355
Treatment	Lane	N = 1	N = 2	N = 3	N = 4	N = 5	Lane	N = 1	N = 2	N = 3	N = 4	N = 5
NS	1	26197815	21489986	44926752	26415698.5	16733725	1	153582513	1.27E+08	52417521	82393758	103256953.5
TNF	2	14930732	12186838.5	27254724	4499160	11046245	2	102668871.5	96815829	38214312.5	71386263	88333700

		Normalized Values				
		BIRC3				
Treatment	Lane	N = 1	N = 2	N = 3	N = 4	N = 5
NS	1	0.040903602	0.191656	0.302943	0.395637	0.129238
IL1B	2	0.04578	0.985007	0.107629	0.337046	0.128658
Treatment	Lane	N = 1	N = 2	N = 3	N = 4	N = 5
NS	1	0.170894	0.171398	0.868313	0.320723	0.167
TNF	2	0.145243816	0.12620828	0.721919953	0.062986646	0.128

Figure 6A - A549 cells: protein

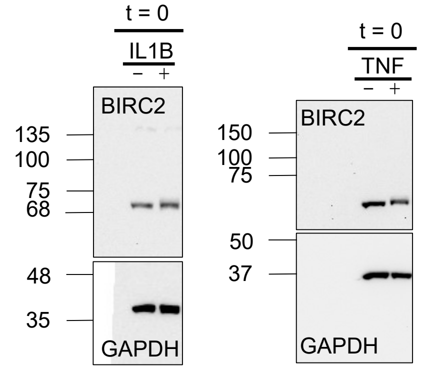

Chemiluminescence

		Adj. Total Band Vol.						Adj. Total Band Vol.				
		BIRC2						GAPDH				
Treatment	Lane	N = 1	N = 2	N = 3	N = 4	N = 5	Lane	N = 1	N = 2	N = 3	N = 4	N = 5
NS	1	94403472	102085900	64657065	61436982	39730845	1	173897080	2.09E+08	166513302	283813787	323811264
IL1B	2	62762970	74566084.5	27465795	63294838	55991191.5	2	126447282	2.53E+08	161126342	187468750	283292342
Treatment	Lane	N = 1	N = 2	N = 3	N = 4	N = 5	Lane	N = 1	N = 2	N = 3	N = 4	N = 5
NS	1	61134030	94260608	27515602	54308880	150703277	1	140001484.5	1.17E+08	116266861	208564211	146203838
TNF	2	30389590.5	42635934	10990809	21651564	98905536	2	103703597	84325669	70674923	143646320	114605855

		Normalized Values				
		BIRC2				
Treatment	Lane	N = 1	N = 2	N = 3	N = 4	N = 5
NS	1	0.542869794	0.487356475	0.388299699	0.216469336	0.122697538
IL1B	2	0.496356814	0.294195107	0.170461233	0.337628741	0.197644564
Treatment	Lane	N = 1	N = 2	N = 3	N = 4	N = 5
NS	1	0.448169	0.79	0.237653	0.260352	1.049
TNF	2	0.293	0.496	0.1543	0.148805	0.871

Figure 6B - A549 cells: protein

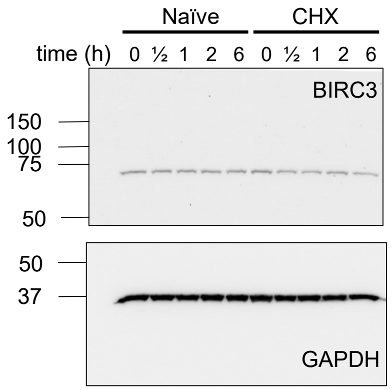

Chemiluminescence

				Adj. Total Band Vol.						Adj. Total Band Vol.				
				BIRC3						GAPDH				
Treatment	CHX	Time	Lane	N = 1	N = 2	N = 3	N = 4	N = 5	Lane	N = 1	N = 2	N = 3	N = 4	N = 5
NS	-	0	1	27964296	32950174	26784906	56225170	35369705	1	137407728	1.57E+08	184015686	40512498	112649950
NS	-	0.5	2	23771625	27052025	19248086	41588580	21348350	2	119933016	1.33E+08	147478902	34940633	78360840
NS	-	1	3	20902250	25367321	19908070	37379925	12626816	3	114019712	1.17E+08	148531356	34053201	55538436
NS	-	2	4	22068375	26772529	20673932	42747395	12148125	4	102463625	1.15E+08	138872634	35990206	63293094
NS	-	6	5	20455750	26334931	13778502	41612835	16966488	5	104355582	1.25E+08	143852478	39592371	63222738
NS	+	0	6	22967500	31031140	12511306	38979600	17155530	6	107731518	1.37E+08	160497896	41894762	45337431
NS	+	0.5	7	23464125	19757920	16162614	33590205	10421440	7	91215875	1.08E+08	151346346	39500734	49073310
NS	+	1	8	20277750	19348336	15842997	40474335	9947145	8	91920750	1.08E+08	125486960	41036094	47256108
NS	+	2	9	18625013	25030187	15706215	42362280	9927650	9	93764300	1.28E+08	121303718	36471288	54755418
NS	+	6	10	17774186	18472569	15413616	33510816	16100674	10	99200368	1.3E+08	146024286	40096980	63684200

				BIRC3/GAPDH (% of t = 0)				
Treatment	CHX	Time	Lane	N = 1	N = 2	N = 3	N = 4	N = 5
NS	-	0	1	100	100	100	100	100
NS	-	0.5	2	186.0336	63.76814	89.86803	127.2924	134.4842
NS	-	1	3	164.8129	74.64153	99.71884	101.6965	103.9139
NS	-	2	4	126.249	84.71455	141.6279	88.80621	58.77197
NS	-	6	5	122.3748	82.04422	121.6021	93.28202	38.96863
NS	+	0	6	100	100	100	100	100
NS	+	0.5	7	46.02112	96.98831	141.8167	92.66664	54.38213
NS	+	1	8	58.1675	77.1762	135.5881	112.808	59.83551
NS	+	2	9	46.87361	78.42609	142.1238	116.9065	63.39063
NS	+	6	10	87.58658	81.04756	118.7335	72.71101	68.25898

Figure 6B - A549 cells: protein

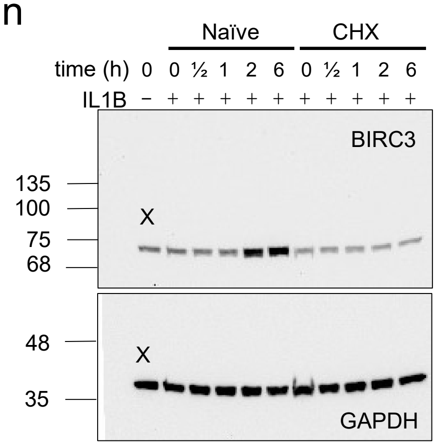

Chemiluminescence

				Adj. Total Band Vol.						Adj. Total Band Vol.				
				BIRC3						GAPDH				
Treatment	CHX	Time	Lane	N = 1	N = 2	N = 3	N = 4	N = 5	Lane	N = 1	N = 2	N = 3	N = 4	N = 5
IL1B	-	0	1	5986395	74462463	76026300	65685110	30881550	1	171699000	58015869	198438326	146101960	194818965
IL1B	-	0.5	2	9161321	51583518	68439168	52733660	29037450	2	204092425	39288078	193585851	164001656	224917275
IL1B	-	1	3	11732096	58340866	74824836	51197517	30675300	3	185669130	45132876	180477696	166135832	227911035
IL1B	-	2	4	21493143	46063963	97242816	99327984	97549350	4	173226396	29261574	212663166	153623692	220149600
IL1B	-	6	5	53701137	137658180	130109072	175931462	142220400	5	163393380	33862374	224664180	162170716	167622345
IL1B	+	0	6	6740296	29876315	87475960	40739139	22292700	6	118886790	43517898	257293344	181459312	225629745
IL1B	+	0.5	7	7236432	16399299	86683840	36142499	20004600	7	137478165	30832650	249484383	178833732	181596525
IL1B	+	1	8	7797036	23294931	77420956	33037196	19114200	8	154904559	37893096	250863225	178299156	234824040
IL1B	+	2	9	6242038	33355944	84722564	34655395	18147900	9	177537613	44214822	359692560	164094020	264307890
IL1B	+	6	10	5842552	49559312	97689500	37269314	18250592	10	272794368	61203978	380456509	170246752	251877150

				BIRC3/GAPDH (% of t = 0)				
Treatment	CHX	Time	Lane	N = 1	N = 2	N = 3	N = 4	N = 5
IL1B	-	0	1	100	100	100	100	100
IL1B	-	0.5	2	98.051	133.2941	143.5666	95.40041	100.3456
IL1B	-	1	3	138.0247	131.2323	120.7801	91.43156	104.6132
IL1B	-	2	4	271.0233	159.8175	214.4344	191.8333	344.4049
IL1B	-	6	5	717.9097	412.7103	877.9922	321.8708	659.4666
IL1B	+	0	6	100	100	100	100	100
IL1B	+	0.5	7	114.9772	53.99769	66.14342	59.96244	85.62196
IL1B	+	1	8	109.9477	62.41113	92.72829	54.9749	63.26688
IL1B	+	2	9	76.79923	76.58894	96.96516	62.65975	53.36778
IL1B	+	6	10	46.78299	82.20654	90.73809	64.95059	56.31849

Figure 6B - A549 cells: protein

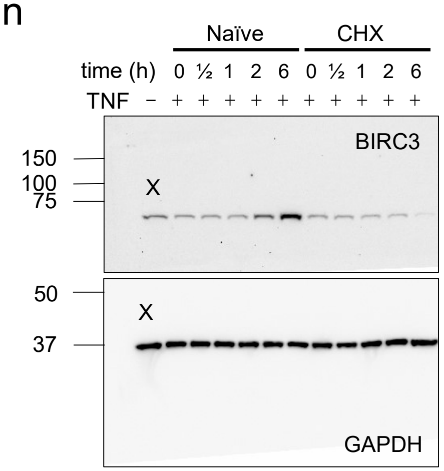

Chemiluminescence

				Adj. Total Band Vol.						Adj. Total Band Vol.				
				BIRC3						GAPDH				
Treatment	CHX	Time	Lane	N = 1	N = 2	N = 3	N = 4	N = 5	Lane	N = 1	N = 2	N = 3	N = 4	N = 5
TNF	-	0	1	14287676	16097864	12490932	30108120	9462265	1	50320981	1.08E+08	103128544	35550515	96780190
TNF	-	0.5	2	4673980	19381216	11586460	27100920	9856085	2	35297912	96307042	94406607	31462795	88662210
TNF	-	1	3	16128712	19239248	10593812	30295608	14186075	3	32159380	1.06E+08	101380016	33414563	92402310
TNF	-	2	4	28648276	37629272	28566185	42865032	21896015	4	31042134	1.05E+08	85798485	36148840	84664190
TNF	-	6	5	95430966	108906480	87628407	124106472	65905545	5	19675868	1.05E+08	83248720	38292072	75394020
TNF	+	0	6	14067842	13763600	11882745	24401328	12630225	6	18533402	96943678	90503113	40878110	79887210
TNF	+	0.5	7	9149130	15124456	10057608	19578216	9980060	7	13182494	1.06E+08	85106325	38093419	79976960
TNF	+	1	8	11470308	13081576	7494399	17505432	9844920	8	17705798	1.04E+08	72014098	33834346	85012720
TNF	+	2	9	10252430	11134760	5148756	15773184	7014955	9	28276373	1.15E+08	97559128	36683010	75623730
TNF	+	6	10	1703094	2532016	1392947	5063352	1219305	10	34098119	1.1E+08	116842904	35218001	77956062

				BIRC3/GAPDH (% of t = 0)				
Treatment	CHX	Time	Lane	N = 1	N = 2	N = 3	N = 4	N = 5
TNF	-	0	1	100	100	100	100	100
TNF	-	0.5	2	25.39	138.6	97.24	119.3	86.89
TNF	-	1	3	96.17	124.4	82.8	125.6	120
TNF	-	2	4	177	247.7	263.8	164.3	202.1
TNF	-	6	5	930.1	714.8	834	448.9	683.3
TNF	+	0	6	100	100	100	100	100
TNF	+	0.5	7	133.1	98.37	93.64	71.19	97.54
TNF	+	1	8	124.2	86.85	82.46	71.67	90.52
TNF	+	2	9	69.53	66.83	41.82	59.56	72.51
TNF	+	6	10	9.578	15.8	9.446	19.92	12.23

Figure 6B - A549 cells: protein

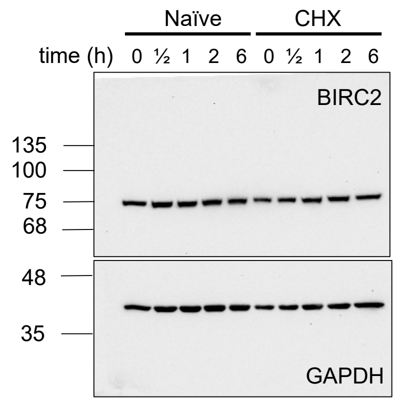

Chemiluminescence

				Adj. Total Band Vol.						Adj. Total Band Vol.				
				BIRC2						GAPDH				
Treatment	CHX	Time	Lane	N = 1	N = 2	N = 3	N = 4	N = 5	Lane	N = 1	N = 2	N = 3	N = 4	N = 5
NS	-	0	1	57295068	123679384	51399059	108202770	170934344	1	140905190	1.28E+08	167088481	219987648	71542565
NS	-	0.5	2	56205705	79170128	49753824	70123528	126538038	2	118029483	84341000	130495860	201509226	56300250
NS	-	1	3	51772149	68974200	51341108	71097111	102701520	3	105500885	76016640	122391060	191578100	61505500
NS	-	2	4	52448217	55886838	63805320	61948656	88182918	4	112385280	83099375	135267990	185372000	50210000
NS	-	6	5	53365488	38149488	65977096	67301304	80488890	5	108059050	92814468	149920980	197992640	59721500
NS	+	0	6	74656701	64841832	56628420	58707096	107329860	6	107910392	1.06E+08	157307040	205424574	60609250
NS	+	0.5	7	59754426	46009974	36832396	65757456	110379024	7	97838600	85193875	135769425	190852353	56693000
NS	+	1	8	58191615	71360513	51367898	51356240	108511812	8	111958480	85031750	158894736	210269780	58396125
NS	+	2	9	56782080	58707070	44498754	60112860	110818854	9	100628484	61901806	157078040	190623015	50832625
NS	+	6	10	50650612	52298991	41245875	70669980	173984382	10	125597700	95421614	140471350	217105206	72961976

				BIRC2/GAPDH (% of t = 0)				
Treatment	CHX	Time	Lane	N = 1	N = 2	N = 3	N = 4	N = 5
NS	-	0	1	100	100	100	100	100
NS	-	0.5	2	133.1601	80.18687	118.9011	88.68699	107.3143
NS	-	1	3	91.13728	61.22612	108.5889	156.2428	101.4706
NS	-	2	4	93.31583	54.63208	89.73071	146.2264	88.82063
NS	-	6	5	105.029	58.0683	79.3373	103.5847	76.69202
NS	+	0	6	100	100	100	100	100
NS	+	0.5	7	101.2868	64.15503	141.8167	121.3097	75.6828
NS	+	1	8	117.4794	76.45216	135.5881	96.84649	62.59182
NS	+	2	9	104.1043	67.35345	142.1238	104.9424	57.53192
NS	+	6	10	59.37686	82.00555	118.7335	62.82365	37.28868

Figure 6B - A549 cells: protein

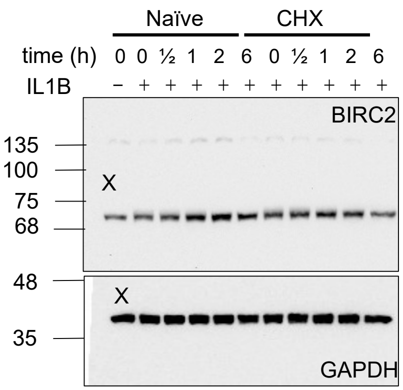

Chemiluminescence

				Adj. Total Band Vol.						Adj. Total Band Vol.				
				BIRC2						GAPDH				
Treatment	CHX	Time	Lane	N = 1	N = 2	N = 3	N = 4	N = 5	Lane	N = 1	N = 2	N = 3	N = 4	N = 5
IL1B	-	0	1	80266564	82848366	36843165	73979132	48732795	1	140015260	2.18E+08	203924316	231072450	332852852
IL1B	-	0.5	2	103055114	51026516	37281663	59960720	56987298	2	205286792	1.43E+08	195485208	156086700	361821436
IL1B	-	1	3	87377251	48933171	36598365	49225836	85078863	3	164822264	1.42E+08	170902852	142574775	307796580
IL1B	-	2	4	84468355	45491134	47329173	70623798	100035684	4	143328096	1.52E+08	139319652	129726275	326403024
IL1B	-	6	5	96955716	76278252	50833791	86896524	73219068	5	125785624	2.71E+08	126430332	127356250	215522364
IL1B	+	0	6	45259376	66283803	18088425	52610544	63249588	6	112879304	2.89E+08	118328368	143865050	233731832
IL1B	+	0.5	7	69111714	45948046	23141709	61170557	70142544	7	143221848	2.34E+08	100121112	149211475	229160932
IL1B	+	1	8	90700627	55183146	32468742	58610695	75843783	8	178212138	2.11E+08	96375676	161626806	299016840
IL1B	+	2	9	94888916	50113046	56838429	52607596	68076891	9	211263394	1.67E+08	145125396	182588413	291994768
IL1B	+	6	10	91253305	69327360	70834542	60196226	48866673	10	239517915	1.51E+08	204770298	213002726	274473675

				BIRC2/GAPDH (% of t = 0)				
Treatment	CHX	Time	Lane	N = 1	N = 2	N = 3	N = 4	N = 5
IL1B	-	0	1	100	100	100	100	100
IL1B	-	0.5	2	103.0575	117.1735	97.78173	112.0217	75.53706
IL1B	-	1	3	108.8313	113.0819	114.6697	100.682	132.5666
IL1B	-	2	4	120.9857	98.46552	126.4713	158.7539	146.9864
IL1B	-	6	5	158.239	92.5177	160.1771	198.9681	162.9327
IL1B	+	0	6	100	100	100	100	100
IL1B	+	0.5	7	99.06349	64.38165	96.09955	119.5478	146.797
IL1B	+	1	8	104.4827	85.88416	85.35877	105.7462	121.6468
IL1B	+	2	9	92.2067	98.42757	65.14697	84.01881	111.8152
IL1B	+	6	10	78.21352	150.9027	71.01817	82.41104	85.38634

Figure 6B - A549 cells: protein

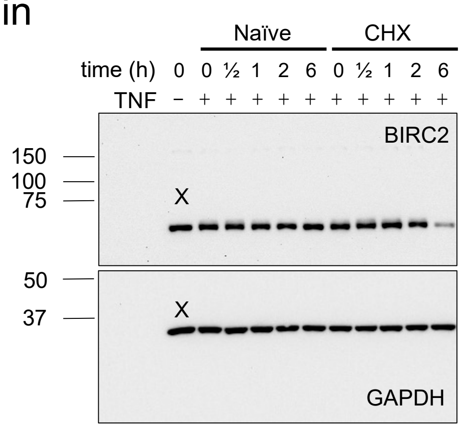

Chemiluminescence

				Adj. Total Band Vol.						Adj. Total Band Vol.				
				BIRC2						GAPDH				
Treatment	CHX	Time	Lane	N = 1	N = 2	N = 3	N = 4	N = 5	Lane	N = 1	N = 2	N = 3	N = 4	N = 5
TNF	-	0	1	29665266	55594740	12936844	27396024	99885960	1	99754902	92297212	75967008	152662080	126209590
TNF	-	0.5	2	36186174	40019187	15003716	20020050	86859054	2	107555444	1E+08	77198160	149278717	122512780
TNF	-	1	3	32252832	46036510	12969556	21121980	84563514	3	114371138	95908500	75981204	143754880	120263260
TNF	-	2	4	33189183	60115536	18229608	32747370	87061068	4	115525584	1.02E+08	77241958	143942880	105370460
TNF	-	6	5	59333394	103304520	21771998	49521948	119686572	5	113475862	96471413	78952588	135794080	108336150
TNF	+	0	6	31113915	29677128	9044774	15907104	97925112	6	107652292	76354125	65382838	134630560	103002120
TNF	+	0.5	7	34691892	34023486	11858006	14320664	93583674	7	115339710	77308250	62273298	147011837	87745959
TNF	+	1	8	19988367	25950672	9386370	12411652	104042880	8	108603468	77269848	72121140	160751085	104176540
TNF	+	2	9	13665573	22898786	6692832	12412788	77416398	9	116659647	85826664	69828720	171815655	99836750
TNF	+	6	10	5549418	7220748	2394744	3223440	19860876	10	109586718	73713400	64311468	167311155	112761456

				BIRC2/GAPDH (% of t = 0)				
Treatment	CHX	Time	Lane	N = 1	N = 2	N = 3	N = 4	N = 5
TNF	-	0	1	100	100	100	100	100
TNF	-	0.5	2	16.6	114.7	80.63	125.9	81.39
TNF	-	1	3	67.24	96.18	96.87	110.6	80.72
TNF	-	2	4	127.1	97.98	118.7	152.9	94.85
TNF	-	6	5	229.2	178.3	216.1	178.7	126.8
TNF	+	0	6	100	100	100	100	100
TNF	+	0.5	7	145.7	102.6	88.82	123.4	122.4
TNF	+	1	8	72.08	62.77	67.78	84.34	114.7
TNF	+	2	9	33.49	39.95	53.84	62.11	89.02
TNF	+	6	10	5.45	17.27	19.77	24.13	20.22

Figure 7A - A549 cells: protein

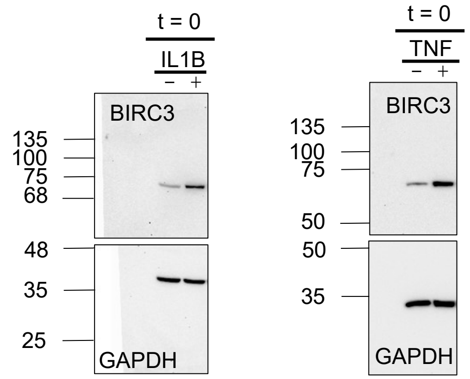

Chemiluminescence

		Adj. Total Band Vol.						Adj. Total Band Vol.				
		BIRC3						GAPDH				
Treatment	Lane	N = 1	N = 2	N = 3	N = 4	N = 5	Lane	N = 1	N = 2	N = 3	N = 4	N = 5
NS	1	8447470	10956759	18918104	15026938	11471760	1	97299156	1.11E+08	2.62E+08	2.84E+08	1.36E+08
IL1B	2	71901792	50671583	1.7E+08	1.18E+08	66334959	2	84585109	56293708	2.06E+08	1.87E+08	1.24E+08
Treatment	Lane	N = 1	N = 2	N = 3	N = 4	N = 5	Lane	N = 1	N = 2	N = 3	N = 4	N = 5
NS	1	7009968	10300123	10168860	26415699	16423330	1	80358850	98181056	37928021	82393758	1.02E+08
TNF	2	39209702	56683140	36089100	91252018	98964670	2	69504930	92333780	34205640	58826698	1.25E+08

		Normalized Values				
		BIRC3				
Treatment	Lane	N = 1	N = 2	N = 3	N = 4	N = 5
NS	1	0.08682	0.09894	0.072256	0.052946	0.084568
IL1B	2	0.921917	0.97026	0.820771	0.661137	0.537034
Treatment	Lane	N = 1	N = 2	N = 3	N = 4	N = 5
NS	1	0.086552	0.10507	0.268264	0.320723	0.16357
TNF	2	0.54564	0.608281	1.107801	1.580289	0.841088

Figure 7B - A549 cells: protein

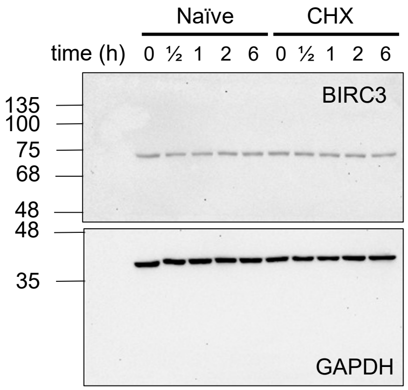

Chemiluminescence

				Adj. Total Band Vol.						Adj. Total Band Vol.				
				BIRC3						GAPDH				
Treatment	CHX	Time	Lane	N = 1	N = 2	N = 3	N = 4	N = 5	Lane	N = 1	N = 2	N = 3	N = 4	N = 5
NS	-	0	1	8989940	11290551	25375976	26773870	16165932	1	1.33E+08	1.11E+08	2.35E+08	2.53E+08	1.72E+08
NS	-	0.5	2	9175666	10166310	21741384	29681048	10813870	2	1.3E+08	81375608	2.13E+08	2.99E+08	1.54E+08
NS	-	1	3	7665364	7608150	17131128	27497224	10647100	3	1.34E+08	62896960	1.73E+08	2.42E+08	1.62E+08
NS	-	2	4	7207381	7760775	17616816	26999156	12818510	4	1.36E+08	56361648	1.82E+08	2.46E+08	1.19E+08
NS	-	6	5	5918596	7727610	18812472	25207292	13739570	5	1.26E+08	45049552	1.81E+08	2.28E+08	1.15E+08
NS	+	0	6	5420447	6756585	21394632	23734244	15758150	6	99627048	32606728	1.92E+08	2.07E+08	1.03E+08
NS	+	0.5	7	5089455	5703060	17995320	19977168	13723590	7	1.02E+08	23357944	1.98E+08	1.92E+08	99814820
NS	+	1	8	5627619	5577000	14266224	26346928	12186110	8	1.07E+08	20089600	1.74E+08	2.27E+08	94071710
NS	+	2	9	5169485	5613795	13948368	21320492	13021490	9	1.12E+08	38488612	1.98E+08	2.25E+08	1.43E+08
NS	+	6	10	5633990	4342956	13929672	29415390	12453098	10	90273416	39250944	2.6E+08	2.29E+08	1.57E+08

				BIRC3/GAPDH (% of t = 0)				
Treatment	CHX	Time	Lane	N = 1	N = 2	N = 3	N = 4	N = 5
NS	-	0	1	100	100	100	100	100
NS	-	0.5	2	115.4008	80.89943	93.11288	89.8877	56.81632
NS	-	1	3	93.81559	78.32957	90.17132	103.0049	53.22259
NS	-	2	4	86.72938	89.16569	88.032	99.42815	87.22113
NS	-	6	5	77.34433	111.0788	94.48031	100.1501	96.59904
NS	+	0	6	100	100	100	100	100
NS	+	0.5	7	82.12545	158.1065	82.59752	94.53831	111.0374
NS	+	1	8	86.45485	179.7652	74.79433	105.3835	104.6171
NS	+	2	9	75.71697	94.44972	64.0917	85.92572	73.37436
NS	+	6	10	102.4035	71.64926	48.83716	116.3231	63.92351

Figure 7B - A549 cells: protein

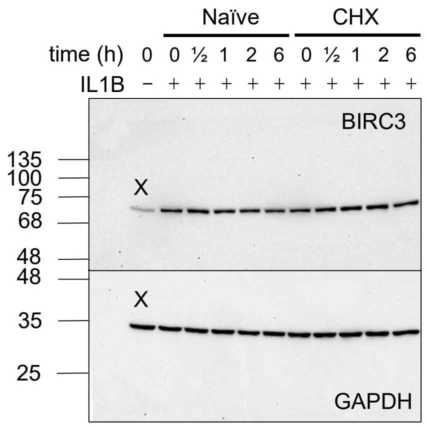

Chemiluminescence

				Adj. Total Band Vol.						Adj. Total Band Vol.				
				BIRC3						GAPDH				
Treatment	CHX	Time	Lane	N = 1	N = 2	N = 3	N = 4	N = 5	Lane	N = 1	N = 2	N = 3	N = 4	N = 5
IL1B	-	0	1	72258480	56377365	2.01E+08	1.2E+08	69123183	1	61161819	74651612	2.26E+08	2.31E+08	1.23E+08
IL1B	-	0.5	2	69518304	56387925	1.85E+08	1.1E+08	82172631	2	55686678	69944488	1.99E+08	1.56E+08	1.36E+08
IL1B	-	1	3	67017168	45787500	1.63E+08	1.23E+08	63686496	3	74168487	37586300	2.01E+08	1.43E+08	1.22E+08
IL1B	-	2	4	73385280	37853475	1.72E+08	1.32E+08	56637231	4	99402315	32926648	2.19E+08	1.3E+08	1.06E+08
IL1B	-	6	5	72600624	40772490	1.44E+08	1.07E+08	55117191	5	1.14E+08	37380932	1.93E+08	1.27E+08	1.19E+08
IL1B	+	0	6	71545104	44965800	1.39E+08	1.15E+08	63546735	6	1.08E+08	37935804	1.85E+08	1.44E+08	1.25E+08
IL1B	+	0.5	7	65073888	39263895	1.3E+08	1.07E+08	76688403	7	1.11E+08	26326148	1.68E+08	1.49E+08	1.32E+08
IL1B	+	1	8	64672704	44956230	1.51E+08	1.09E+08	86285007	8	1.1E+08	38412760	1.98E+08	1.62E+08	1.47E+08
IL1B	+	2	9	62801280	46920390	1.82E+08	1.36E+08	99517782	9	90579219	45683716	2.41E+08	1.83E+08	1.48E+08
IL1B	+	6	10	54662664	34756824	1.68E+08	1.16E+08	78679152	10	86052792	68011312	2.65E+08	2.13E+08	1.44E+08

				BIRC3/GAPDH (% of t = 0)				
Treatment	CHX	Time	Lane	N = 1	N = 2	N = 3	N = 4	N = 5
IL1B	-	0	1	100	100	100	100	100
IL1B	-	0.5	2	135.4116	83.08922	113.5753	106.9008	112.7383
IL1B	-	1	3	98.01098	125.5537	98.75927	130.1656	97.36344
IL1B	-	2	4	80.07935	118.4869	95.57608	153.3397	99.26129
IL1B	-	6	5	68.79766	112.4163	90.62749	127.3669	86.3514
IL1B	+	0	6	100	100	100	100	100
IL1B	+	0.5	7	63.64044	153.7157	94.24064	108.0365	108.1964
IL1B	+	1	8	63.84968	120.622	92.90361	102.1509	109.0559
IL1B	+	2	9	75.20523	105.8552	91.69855	112.4604	124.9026
IL1B	+	6	10	68.90233	52.67093	77.17263	82.70319	101.7651

Figure 7B - A549 cells: protein

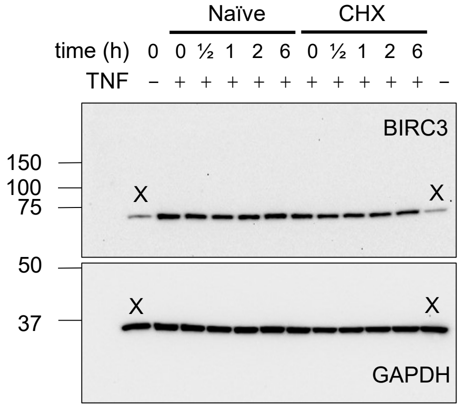

Chemiluminescence

				Adj. Total Band Vol.						Adj. Total Band Vol.				
				BIRC3						GAPDH				
Treatment	CHX	Time	Lane	N = 1	N = 2	N = 3	N = 4	N = 5	Lane	N = 1	N = 2	N = 3	N = 4	N = 5
TNF	-	0	1	55149860	67540995	36463440	93695823	95292260	1	77064350	1E+08	26909400	67618418	92356470
TNF	-	0.5	2	49801874	49322012	30670560	80314012	65514400	2	69120480	98791440	25891080	64478722	1.02E+08
TNF	-	1	3	33401766	40007050	32459040	58392142	63245140	3	66997480	92288130	36194760	56696360	1.2E+08
TNF	-	2	4	31309114	49472426	45694440	69713470	71394260	4	62667880	94007760	38671200	56207865	1.35E+08
TNF	-	6	5	32989422	55604895	61470960	97581475	1.08E+08	5	69909290	90226950	42805800	53187645	1.58E+08
TNF	+	0	6	23269544	45825284	35714760	88808212	1.03E+08	6	61945510	84403550	41501880	50034978	1.58E+08
TNF	+	0.5	7	23415824	33379294	38095320	69325138	83464500	7	51332930	68790590	48000186	49952672	1.34E+08
TNF	+	1	8	21881262	34096066	44683440	77876557	92491280	8	55815870	74668880	54528840	60473777	1.35E+08
TNF	+	2	9	28351608	33895514	38380320	81104415	75967920	9	55058520	81823170	56539130	70651609	1.32E+08
TNF	+	6	10	29156190	32403819	14427000	67676785	69074740	10	61069140	90485230	30182185	70031976	1.06E+08

				BIRC3/GAPDH (% of t = 0)				
Treatment	CHX	Time	Lane	N = 1	N = 2	N = 3	N = 4	N = 5
TNF	-	0	1	100	100	100	100	100
TNF	-	0.5	2	132.0484	82.07618	106.9325	78.82036	76.47909
TNF	-	1	3	91.3703	71.26665	80.95211	65.17225	62.54482
TNF	-	2	4	91.56297	86.51575	106.663	78.48436	63.10157
TNF	-	6	5	86.48363	101.3147	129.63	116.0968	81.20521
TNF	+	0	6	100	100	100	100	100
TNF	+	0.5	7	83.60023	79.77077	71.64187	87.82042	73.99747
TNF	+	1	8	71.84702	75.06894	73.97049	81.48978	81.24818
TNF	+	2	9	94.37289	68.10227	61.27702	72.64169	68.58038
TNF	+	6	10	87.499	58.87273	43.14828	61.15146	77.66801

Figure 8B - A549 cells: protein

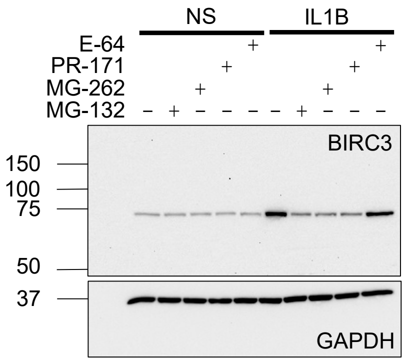

Chemiluminescence

			Adj. Total Band Vol.						Adj. Total Band Vol.				
			BIRC3						GAPDH				
Treatment	Inhibitor	Lane	N = 1	N = 2	N = 3	N = 4	N = 5	Lane	N = 1	N = 2	N = 3	N = 4	N = 5
NS	-	1	263313	14678638	3556050	17947412	1.42E+08	1	9399264	1.88E+08	1.58E+08	2.29E+08	1.58E+08
NS	MG-132	2	383760	12245701	8307600	17787212	1.13E+08	2	9101664	1.52E+08	1.94E+08	1.59E+08	1.94E+08
NS	MG-262	3	430951	14667229	4102050	15385081	1E+08	3	7492000	1.31E+08	96834255	1.57E+08	96834255
NS	PR-171	4	390853	11589463	3428100	15636101	1.09E+08	4	6218496	1.2E+08	89999644	1.37E+08	89999644
NS	E-64	5	533123	10215699	4202100	13570700	1.27E+08	5	5340960	1.31E+08	77487690	88999467	77487690
IL1B	-	6	5105853	1.07E+08	91068600	1.08E+08	1.26E+08	6	6210784	1.2E+08	58798810	89629462	58798810
IL1B	MG-132	7	952184	21269720	4354350	40237130	1.14E+08	7	6018816	82614340	1.13E+08	98419970	1.13E+08
IL1B	MG-262	8	793268	20627657	4389750	46572779	1.43E+08	8	6118080	1.03E+08	1.07E+08	1.25E+08	1.07E+08
IL1B	PR-171	9	769201	22196718	5108700	35185587	1.09E+08	9	6372832	1.47E+08	1.16E+08	1.39E+08	1.16E+08
IL1B	E-64	10	5699549	1.17E+08	78422084	1.16E+08	1.69E+08	10	7287381	1.47E+08	1.63E+08	1.23E+08	1.63E+08

			Normalized Values				
			BIRC3				
Treatment	Inhibitor	Lane	N = 1	N = 2	N = 3	N = 4	N = 5
NS	-	1	0.028014	0.077892	0.022451	0.078232	0.895887
NS	MG-132	2	0.042164	0.080799	0.042872	0.112059	0.585681
NS	MG-262	3	0.057521	0.111939	0.042362	0.098128	1.037
NS	PR-171	4	0.062853	0.096621	0.03809	0.114309	1.213196
NS	E-64	5	0.099818	0.077989	0.054229	0.152481	1.634649
IL1B	-	6	0.822095	0.894871	1.548817	1.19966	2.145405
IL1B	MG-132	7	0.158201	0.257458	0.038615	0.408831	1.011903
IL1B	MG-262	8	0.12966	0.201245	0.041099	0.371255	1.343187
IL1B	PR-171	9	0.1207	0.151119	0.04423	0.253329	0.941759
IL1B	E-64	10	0.782112	0.796898	0.481073	0.944756	1.037091

Figure 8B - A549 cells: protein

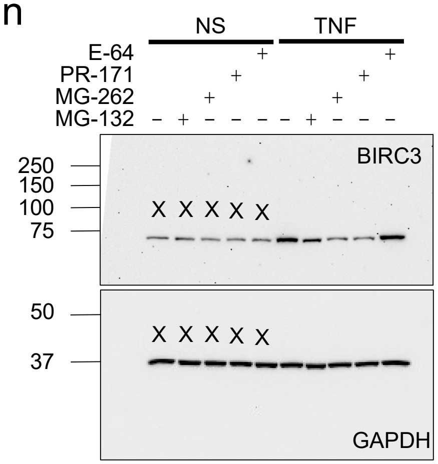

Chemiluminescence

			Adj. Total Band Vol.							Adj. Total Band Vol.					
			BIRC3							GAPDH					
Treatment	Inhibitor	Lane	N = 1	N = 2	N = 3	N = 4	N = 5	N = 6	Lane	N = 1	N = 2	N = 3	N = 4	N = 5	N = 6
NS	-	1	7698192	8201800	17312222	22763007	24316202	37498556	1	1.29E+08	57696330	1.22E+08	78315291	2.22E+08	121882314
NS	MG-132	2	9176397	11821319	13243768	24919146	19564524	3780897	2	1.13E+08	58071320	98343665	72727224	2.39E+08	116160171
NS	MG-262	3	12731410	12665650	9730923	15427278	24492699	22437868	3	1.09E+08	43937656	1.01E+08	75992584	2.07E+08	107166964
NS	PR-171	4	8560034	13416136	11198853	17284167	32594016	23995065	4	1.18E+08	61831312	98815789	73849224	2.19E+08	114219250
NS	E-64	5	13351338	16106398	10430820	20882350	26116608	29296066	5	1.25E+08	60402632	1.03E+08	84278384	1.74E+08	109246420
TNF	-	6	69960280	78364664	75748695	1.02E+08	1.72E+08	218672865	6	1.33E+08	55036208	85846789	79591552	2.15E+08	113850076
TNF	MG-132	7	29394276	39167850	34135969	45736884	61584327	49894439	7	1.47E+08	65962496	88568116	70198656	2.31E+08	120715663
TNF	MG-262	8	10891758	11107890	9093651	21608400	76062210	22424996	8	1.46E+08	50296005	92593404	74132845	2.54E+08	123357956
TNF	PR-171	9	16247736	16578960	20704326	20847465	54354720	28300609	9	1.48E+08	82627776	1.07E+08	70572510	2.44E+08	130404935
TNF	E-64	10	46425552	49462190	1.37E+08	1.25E+08	1.66E+08	164220509	10	99532592	66327891	1.4E+08	96048881	2.41E+08	128557401

			Normalized Values					
			BIRC3					
Treatment	Inhibitor	Lane	N = 1	N = 2	N = 3	N = 4	N = 5	N = 6
NS	-	1	0.059538	0.142155	0.142299	0.290659	0.109315	0.307662
NS	MG-132	2	0.081496	0.203566	0.134668	0.342638	0.081839	0.032549
NS	MG-262	3	0.117335	0.288264	0.096575	0.20301	0.118529	0.209373
NS	PR-171	4	0.072594	0.21698	0.113331	0.234047	0.149047	0.210079
NS	E-64	5	0.107078	0.266651	0.101594	0.247778	0.149931	0.268165
TNF	-	6	0.524842	1.423875	0.882371	1.282778	0.797836	1.920709
TNF	MG-132	7	0.199297	0.59379	0.385421	0.651535	0.266184	0.413322
TNF	MG-262	8	0.074554	0.22085	0.098211	0.291482	0.299841	0.181788
TNF	PR-171	9	0.109812	0.200646	0.194401	0.295405	0.222435	0.217021
TNF	E-64	10	0.466436	0.745722	0.978962	1.298132	0.691831	1.27741

Figure 8B - A549 cells: protein

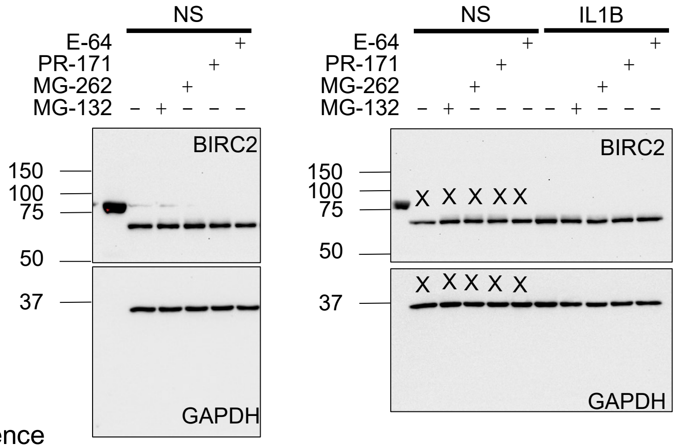

Chemiluminescence

			Adj. Total Band Vol.										
			BIRC2						GAPDH				
Treatment	Inhibitor	Lane	N = 1	N = 2	N = 3	N = 4	N = 5	Lane	N = 1	N = 2	N = 3	N = 4	N = 5
NS	-	1	3497718	1.14E+08	58344770	1.95E+08	79652864	1	5284314	1.24E+08	1.5E+08	2.04E+08	59004152
NS	MG-132	2	2660840	1.1E+08	98886435	1.96E+08	81436528	2	4678542	1.12E+08	1.58E+08	1.31E+08	47994900
NS	MG-262	3	2678452	1.18E+08	88853905	1.48E+08	64087557	3	4768995	1.1E+08	1.22E+08	1.43E+08	40986420
NS	PR-171	4	1718156	90672582	86289120	1.18E+08	58976579	4	4862055	1.09E+08	1.33E+08	1.09E+08	56352010
NS	E-64	5	1801116	79828826	1.01E+08	1.39E+08	68033595	5	4021430	93462081	1.46E+08	1.11E+08	54400500
IL1B	-	6	3019030	1.18E+08	1.52E+08	1.19E+08	1.27E+08	6	4146432	78815100	1.5E+08	1.12E+08	63600072
IL1B	MG-132	7	1301384	1.04E+08	1.03E+08	1.16E+08	1.02E+08	7	4031328	99006300	95502968	84627396	50207187
IL1B	MG-262	8	1940133	88406164	87480605	1.02E+08	82521398	8	4721984	1.12E+08	92912920	1.1E+08	57486346
IL1B	PR-171	9	2192182	1.05E+08	1.05E+08	1.02E+08	83491187	9	4604864	1.07E+08	87124952	1.05E+08	46439972
IL1B	E-64	10	4594926	1.58E+08	1.18E+08	1.31E+08	91979549	10	5370160	1.19E+08	90870234	2.09E+08	52059316

			Normalized Values				
			BIRC2				
Treatment	Inhibitor	Lane	N = 1	N = 2	N = 3	N = 4	N = 5
NS	-	1	0.661906	0.912223	0.389817	0.956014	1.349954
NS	MG-132	2	0.568733	0.987412	0.625811	1.499882	1.696775
NS	MG-262	3	0.561639	1.067793	0.729586	1.03255	1.563629
NS	PR-171	4	0.353381	0.829237	0.650034	1.081023	1.046575
NS	E-64	5	0.447879	0.854131	0.690051	1.255634	1.250606
IL1B	-	6	0.728103	1.493039	1.014558	1.066555	1.992182
IL1B	MG-132	7	0.322818	1.051468	1.077726	1.370555	2.029866
IL1B	MG-262	8	0.410872	0.786628	0.941533	0.925601	1.435496
IL1B	PR-171	9	0.476058	0.989128	1.205535	0.970186	1.79783
IL1B	E-64	10	0.85564	1.332729	1.296505	0.624455	1.766822

Figure 8B - A549 cells: protein

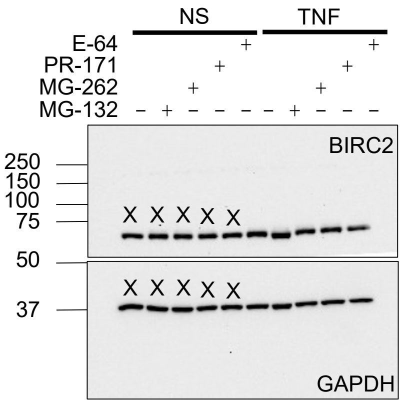

Chemiluminescence

			Adj. Total Band Vol.							Adj. Total Band Vol.					
			BIRC2							GAPDH					
Treatment	Inhibitor	Lane	N = 1	N = 2	N = 3	N = 4	N = 5	N = 6	Lane	N = 1	N = 2	N = 3	N = 4	N = 5	N = 6
NS	-	1	75830560	65864760	90410328	22418624	55205020	64709586	1	1.35E+08	1.35E+08	98709903	17109015	2.46E+08	102294820
NS	MG-132	2	78522340	68869450	99522150	33904668	61579310	74069146	2	1.19E+08	1.08E+08	91978568	18864000	2.27E+08	73706947
NS	MG-262	3	74267570	62062520	85847124	24297904	51245090	70122166	3	1.21E+08	99025815	86405711	19285034	2.39E+08	89039410
NS	PR-171	4	66843660	60226400	84446254	18569594	47255910	58405727	4	1.08E+08	84471891	81775316	16226839	2.23E+08	94949361
NS	E-64	5	73033220	71117410	95936156	43483460	49064210	52336107	5	1.09E+08	84238210	84026873	19580177	1.93E+08	102871110
TNF	-	6	88401170	85959770	91313763	65235043	63158550	47524214	6	95062096	84962600	82234168	24121554	1.92E+08	99576465
TNF	MG-132	7	1.1E+08	1.03E+08	1.11E+08	90805864	71023680	40549175	7	93187328	83192192	71273088	25655848	1.86E+08	103009113
TNF	MG-262	8	62252840	61808240	65610960	38474462	87591920	26576489	8	1.12E+08	77625605	83873044	24989300	1.71E+08	74501367
TNF	PR-171	9	63017760	66358500	75965962	40393626	67880800	40039082	9	1.09E+08	86317187	1.03E+08	29904840	1.77E+08	70152310
TNF	E-64	10	45541990	44648760	52107100	14442580	88486190	58438540	10	74786775	70408369	92106011	17868840	1.97E+08	78562957

			Normalized Values					
			BIRC2					
Treatment	Inhibitor	Lane	N = 1	N = 2	N = 3	N = 4	N = 5	N = 6
NS	-	1	0.56032	0.488704	0.91592	1.31034	0.224629	0.632579
NS	MG-132	2	0.662035	0.637839	1.082015	1.797321	0.271587	1.004914
NS	MG-262	3	0.611887	0.626731	0.993535	1.259936	0.21449	0.787541
NS	PR-171	4	0.618878	0.712976	1.032662	1.144375	0.212326	0.615125
NS	E-64	5	0.667578	0.844242	1.141732	2.22079	0.254508	0.508754
TNF	-	6	0.929931	1.011737	1.110411	2.70443	0.328494	0.477264
TNF	MG-132	7	1.178938	1.243469	1.552379	3.539383	0.382858	0.393646
TNF	MG-262	8	0.556612	0.796235	0.782265	1.539637	0.511356	0.356725
TNF	PR-171	9	0.577419	0.768775	0.737544	1.350739	0.383563	0.570745
TNF	E-64	10	0.608958	0.63414	0.56573	0.808255	0.449892	0.743843

Figure 8C - A549 cells: protein

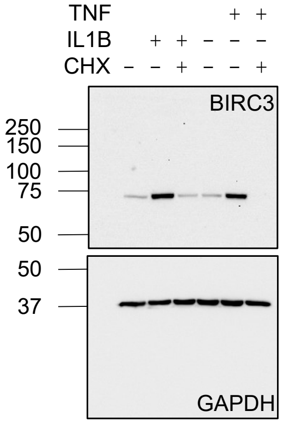

Chemiluminescence

			Adj. Total Band Vol.							Adj. Total Band Vol.					
			BIRC3							GAPDH					
Treatment	CHX	Lane	N = 1	N = 2	N = 3	N = 4	N = 5	N = 6	Lane	N = 1	N = 2	N = 3	N = 4	N = 5	N = 6
NS	-	1	2917491	23380336	13109421	9824451	14865724	22514087	1	60689520	156347008	106337896	109065255	164400333	255845887
IL1B	-	2	108540576	252409984	83664528	131091525	149363043	151456443	2	75414630	98383108	115390153	100974566	143148582	193855233
IL1B	+	3	6355008	6534480	14183128	7427086	12853685	15025811	3	66553900	115114284	112249039	129365236	183129626	165320254
NS	-	4			8482903	8530579	18555381	16854069	4			133426251	137323950	190687628	231267278
TNF	-	5	36747216	104996432	51275115	91706907	107494533	91568849	5	63982760	97204195	148361040	138526850	200885966	277162215
TNF	+	6	357696	52592	2680740	53901	220108	102831	6	55357165	69446595	151488630	135126600	172231125	172928899

			Adj. Total Band Vol.					
			BIRC3					
Treatment	CHX	Lane	N = 1	N = 2	N = 3	N = 4	N = 5	N = 6
NS	-	1	0.048072	0.149541	0.123281	0.090079	0.090424	0.087999
IL1B	-	2	1.439251	2.565583	0.725058	1.298263	1.043413	0.781286
IL1B	+	3	0.095487	0.056765	0.126354	0.057412	0.070189	0.090889
NS	-	4	#DIV/0!	#DIV/0!	0.063577	0.06212	0.097308	0.072877
TNF	-	5	0.57433	1.080164	0.34561	0.662015	0.535102	0.33038
TNF	+	6	0.006462	0.000757	0.017696	0.000399	0.001278	0.000595

Figure 8C - A549 cells: protein

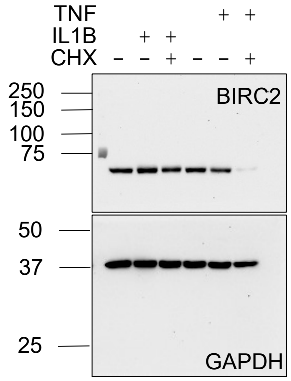

Chemiluminescence

			Adj. Total Band Vol.							Adj. Total Band Vol.					
			BIRC2							GAPDH					
Treatment	CHX	Lane	N = 1	N = 2	N = 3	N = 4	N = 5	N = 6	Lane	N = 1	N = 2	N = 3	N = 4	N = 5	N = 6
NS	-	1	21666692	166253052	31907601	96624652	119048864	148515768	1	178033515	271453912	160436409	162858496	273017176	453050368
IL1B	-	2	123914142	188317264	41063737	116843727	135059904	178337349	2	197437080	240402325	158158395	136668840	208330604	272718044
IL1B	+	3	67623696	121880372	24627426	61821510	82986300	111974143	3	173877364	231227716	150402825	109342156	212755954	297988064
NS	-	4	18041959	256078929	36910403	71272880	105180903	191576443	4	192209745	192209745	148003020	92729696	207223362	320882904
TNF	-	5	66245456	110718368	42460791	50732448	69690879	168969438	5	178304856	241270065	128200383	71302000	178588366	145519842
TNF	+	6	12290572	8369904	11728635	275706	2929572	1617110	6	160774924	182956800	101246364	69690361	138302164	107229180

			Adj. Total Band Vol.					
			BIRC2					
Treatment	CHX	Lane	N = 1	N = 2	N = 3	N = 4	N = 5	N = 6
NS	-	1	0.1217	0.612454	0.19888	0.593304	0.436049	0.327813
IL1B	-	2	0.627613	0.783342	0.259637	0.854941	0.648296	0.653926
IL1B	+	3	0.388916	0.527101	0.163743	0.565395	0.390054	0.375767
NS	-	4	0.093866	1.332289	0.24939	0.768609	0.507573	0.597029
TNF	-	5	0.371529	0.458898	0.331206	0.711515	0.390232	1.161144
TNF	+	6	0.076446	0.045748	0.115843	0.003956	0.021182	0.015081

Figure 8C - A549 cells: protein

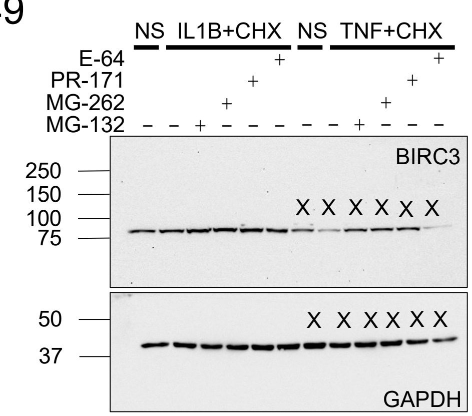

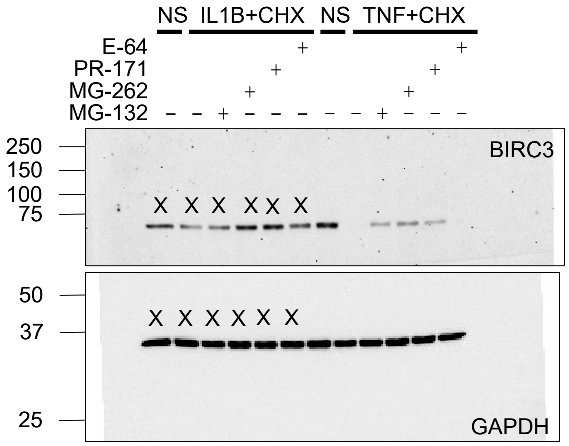

Chemiluminescence

				Adj. Total Band Vol.							Adj. Total Band Vol.					
				BIRC3							GAPDH					
Treatment	CHX	Inhibitor	Lane	N = 1	N = 2	N = 3	N = 4	N = 5	N = 6	Lane	N = 1	N = 2	N = 3	N = 4	N = 5	N = 6
NS	-	-	1	2917491	23380336	13109421	9824451	14865724	22514087	1	60689520	1.56E+08	1.06E+08	1.09E+08	1.64E+08	2.56E+08
IL1B	+	-	2	6355008	6534480	63157190	19673827	15689940	1.07E+08	2	66553900	1.15E+08	86616877	1.22E+08	88074984	1.94E+08
IL1B	+	MG-132	3	5184864	11176712	94826172	18929804	20654900	1.05E+08	3	55305504	75250571	95062517	93503648	36387298	1.61E+08
IL1B	+	MG-262	4	5972256	5733136	92679294	31306271	43788920	1.29E+08	4	53524368	75573991	86802940	1.15E+08	1.12E+08	1.91E+08
IL1B	+	PR-171	5	7615584	5092000	83518252	28582949	43983660	1.08E+08	5	52380700	82711211	1.02E+08	1.45E+08	94954812	192412001
IL1B	+	E-64	6	5580000	2567432	61203204	13656331	28757120	78430684	6	43908400	86195355	92761116	1.33E+08	98408126	2.15E+08
NS	-	-	7	2917491	23380336	13109421	9824451	14865724	22514087	7	60689520	1.56E+08	1.06E+08	1.09E+08	1.64E+08	2.56E+08
TNF	+	-	8	357696	52592	9764072	164222	201180	960310	8	55357165	69446595	87981936	1.05E+08	1.25E+08	1.38E+08
TNF	+	MG-132	9	2613312	201096	42513596	3651663	8700580	42133616	9	62196633	82099853	80209394	1.14E+08	1.02E+08	1.02E+08
TNF	+	MG-262	10	3872448	4440376	45512884	4444042	14320600	63440220	10	67296350	1.2E+08	73116224	1.12E+08	1.21E+08	1.04E+08
TNF	+	PR-171	11	6509664	2118728	45085600	2519536	7126420	48268318	11	57518020	1.29E+08	85201857	73978161	1.19E+08	72727441
TNF	+	E-64	12	4596138	249432	6438848	220585	197680	3441678	12	98153640	1.39E+08	72629025	52872701	74573447	78697769

				Normalized Values					
				BIRC3					
Treatment	CHX	Inhibitor	Lane	N = 1	N = 2	N = 3	N = 4	N = 5	N = 6
NS	-	-	1	0.048072	0.149541	0.123281	0.090079	0.090424	0.087999
IL1B	+	-	2	0.095487	0.056765	0.729156	0.161119	0.178143	0.552448
IL1B	+	MG-132	3	0.09375	0.148527	0.997514	0.20245	0.56764	0.648676
IL1B	+	MG-262	4	0.11158	0.075861	1.067698	0.272459	0.390773	0.67298
IL1B	+	PR-171	5	0.145389	0.061564	0.820351	0.196959	0.463206	0.563013
IL1B	+	E-64	6	0.127083	0.029786	0.659794	0.102854	0.292223	0.365058
NS	-	-	7	0.048072	0.149541	0.123281	0.090079	0.090424	0.087999
TNF	+	-	8	0.006462	0.000757	0.110978	0.00157	0.00161	0.006973
TNF	+	MG-132	9	0.042017	0.002449	0.530033	0.031964	0.085	0.41298
TNF	+	MG-262	10	0.057543	0.037083	0.622473	0.0397	0.118364	0.608663
TNF	+	PR-171	11	0.113176	0.01647	0.529162	0.034058	0.059739	0.663688
TNF	+	E-64	12	0.046826	0.001792	0.088654	0.004172	0.002651	0.043733

Figure 8C - A549 cells: protein

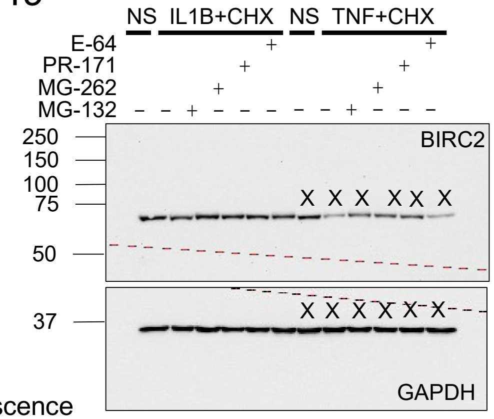

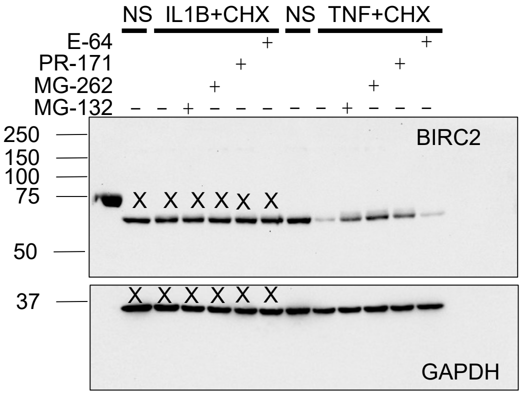

Chemiluminescence

				Adj. Total Band Vol.							Adj. Total Band Vol.					
				BIRC2							GAPDH					
Treatment	CHX	Inhibitor	Lane	N = 1	N = 2	N = 3	N = 4	N = 5	N = 6	Lane	N = 1	N = 2	N = 3	N = 4	N = 5	N = 6
NS	-	-	1	21666692	1.66E+08	31907601	96624652	1.19E+08	1.49E+08	1	1.78E+08	2.71E+08	1.6E+08	1.63E+08	2.73E+08	453050368
IL1B	+	-	2	67623696	1.22E+08	54240690	89872116	16190797	89423628	2	1.74E+08	2.31E+08	95996180	2.13E+08	1.98E+08	100691085
IL1B	+	MG-132	3	54305722	1.22E+08	82531981	66628079	20950862	91486185	3	85183232	2.36E+08	1.14E+08	1.69E+08	1.4E+08	94611384
IL1B	+	MG-262	4	51665604	1.3E+08	67942000	63695464	44359367	1.09E+08	4	80215350	2.43E+08	79114880	1.22E+08	1.4E+08	114474160
IL1B	+	PR-171	5	58026678	1.26E+08	68140835	78714510	44781327	1.14E+08	5	1.03E+08	2.44E+08	90562759	97611610	1.32E+08	116172976
IL1B	+	E-64	6	36888506	1.02E+08	56974800	65841760	29150175	71897847	6	63809902	2.69E+08	96977381	80921912	1.46E+08	113557760
NS	-	-	7	21666692	1.66E+08	31907601	96624652	1.19E+08	1.49E+08	7	1.78E+08	2.71E+08	1.6E+08	1.63E+08	2.73E+08	453050368
TNF	+	-	8	12290572	8369904	15588503	2952579	502242	524349	8	1.61E+08	1.83E+08	1.15E+08	88339854	1.09E+08	10814310
TNF	+	MG-132	9	33524958	53277204	33450165	27191290	9317096	21626346	9	1.75E+08	2.11E+08	1.09E+08	1.13E+08	1.13E+08	52485862
TNF	+	MG-262	10	40192778	80705876	36097327	42783335	14199091	28178283	10	1.38E+08	2.58E+08	93507802	1.61E+08	1.27E+08	74654684
TNF	+	PR-171	11	36663228	62055840	40182371	30895668	7985182	12443109	11	1.81E+08	2.6E+08	1.04E+08	2.21E+08	1.17E+08	47588616
TNF	+	E-64	12	18927586	9473926	15237586	3441703	536081	155085	12	2.17E+08	2.49E+08	1.19E+08	2.26E+08	1.15E+08	8490636

				Normalized Values					
				BIRC2					
Treatment	CHX	Inhibitor	Lane	N = 1	N = 2	N = 3	N = 4	N = 5	N = 6
NS	-	-	1	0.1217	0.612454	0.19888	0.593304	0.436049	0.327813
IL1B	+	-	2	0.388916	0.527101	0.56503	0.421611	0.081754	0.888099
IL1B	+	MG-132	3	0.637517	0.515533	0.724096	0.393491	0.149284	0.966968
IL1B	+	MG-262	4	0.644086	0.533408	0.858777	0.52323	0.3166	0.948983
IL1B	+	PR-171	5	0.562031	0.515997	0.752416	0.806405	0.338519	0.977343
IL1B	+	E-64	6	0.5781	0.37831	0.587506	0.813646	0.199398	0.633139
NS	-	-	1	0.1217	0.612454	0.19888	0.593304	0.436049	0.327813
TNF	+	-	2	0.076446	0.045748	0.135999	0.033423	0.004621	0.048487
TNF	+	MG-132	3	0.191477	0.253085	0.306271	0.241352	0.082348	0.412041
TNF	+	MG-262	4	0.291864	0.31285	0.386035	0.265925	0.11174	0.377448
TNF	+	PR-171	5	0.202587	0.238268	0.386458	0.139662	0.068279	0.261472
TNF	+	E-64	6	0.087377	0.038088	0.127549	0.015205	0.004666	0.018265

Supplemental Fig. S1 - NF-κB activity

	Fold / NS				
log [IL1B (ng/ml)]	N = 1	N = 2	N = 3	N = 4	N = 5
-2.5	1.595611	1.28083	1.135665	1.110941	1.262993
-2	2.063932	1.60974	1.464891	1.457606	1.806812
-1.5	3.893382	2.878168	1.950716	2.167933	2.767953
-1	6.298017	4.930786	3.618322	4.69563	4.555151
-0.5	5.717865	4.937782	4.1003	5.667951	5.926998
0	6.495079	5.426413	3.8995	5.765624	5.573483
0.5	6.223603	5.676081	3.951056	5.891979	3.705269
1	5.651569	5.451379	3.700346	6.40025	5.943908

	Fold / NS			
log [TNF (ng/ml)]	N = 1	N = 2	N = 3	N = 4
-2	1.449424	1.614335	1.559763	1.389665
-1.5	2.353872	2.457597	2.363321	2.176358
-1	3.019623	3.332283	2.973156	2.970264
-0.5	4.238063	3.206014	4.271639	4.027409
0	4.770454	5.168764	6.276489	4.127819
0.5	5.310605	6.32326	5.680196	5.097327
1	5.801243	6.32326	4.58192	4.916093
1.5	5.549293	5.956787	4.842971	5.473474
2	5.730734	6.307156	5.772898	5.279468

Supplemental Fig. S2A - A549 cells: mRNA

		BIRC3					
Treatment	Time (h)	N = 1	N = 2	N = 3	N = 4	N = 5	N = 6
NS	0.5	1	1	1	1	1	1
NS	1	0.376986	1.239299	0.692604	0.893278	1.343843	1.421869
NS	2	0.48424	1.168023	0.745708	0.976285	1.717673	1.566174
NS	4	0.481238	0.881055	0.765863	1.02223	1.162238	1.51016
NS	6	0.367296	0.985468	1.419679	1.290995	2.092141	1.779111
NS	8	0.312226	0.956192	1.224942	1.063549	1.765114	2.160969
NS	10	0.395267	1.120858	0.816865	3.426022	1.61612	1.521628
NS	24	0.687389	0.47954	1.859472	2.547107	3.175251	2.08424
Dex	0.5	0.474273	1.171377	0.785654	1.17051	1.548267	0.894783
Dex	1	0.500479	1.130723	1.059161	1.713638	1.321496	0.984553
Dex	2	1.590571	5.252274	2.546194	4.256764	1.356266	3.058956
Dex	4	3.254525	5.333515	4.756141	9.418573	3.646492	5.650627
Dex	6	2.503253	4.769888	8.26144	7.218992	4.882632	6.170979
Dex	8	1.755838	3.066698	8.253209	7.224497	6.252806	3.733268
Dex	10	3.150313	2.998388	7.925919	9.794669	5.227314	3.521437
Dex	24	1.248252	4.468023	6.34973	5.006275	6.695777	6.221036

		BIRC3					
Treatment	Time (h)	N = 1	N = 2	N = 3	N = 4	N = 5	N = 6
NS	0.5	1	1	1	1	1	1
NS	1	0.254995	1.032033	0.743335	1.157557	1.21003	1.236804
NS	2	0.498536	1.26786	0.685218	1.125273	1.67489	1.363899
NS	4	0.586869	0.746048	0.95882	0.821687	1.137316	1.029598
NS	6	0.508844	0.986322	1.33833	0.958574	1.881976	1.576604
NS	8	0.672965	1.241414	1.448543	0.964358	1.750688	1.652409
NS	10	0.378961	0.842863	1.116549	1.13889	1.384137	1.260242
NS	24	1.044355	1.313397	1.455558	1.248118	2.63516	2.306019
Bud	0.5	0.318608	1.298652	0.69233	0.981663	1.23574	1.18104
Bud	1	0.312684	2.05774	1.053203	1.37369	1.103801	1.681492
Bud	2	0.76118	3.78918	1.987228	2.972892	2.299214	3.174732
Bud	4	1.852948	6.708181	3.968844	4.157537	3.727313	3.949394
Bud	6	1.264275	6.524984	5.157063	4.398285	5.363135	5.376965
Bud	8	1.336375	5.728625	5.271167	4.59708	5.364265	3.714561
Bud	10	2.036164	8.463304	5.230251	5.591704	3.781668	4.157867
Bud	24	0.642602	5.537727	4.4669	3.109231	5.578099	7.140703

		BIRC2					
Treatment	Time (h)	N = 1	N = 2	N = 3	N = 4	N = 5	N = 6
NS	0.5	1	1	1	1	1	1
NS	1	1.168764	0.978255	0.895277	1.032054	1.031321	1.282803
NS	2	1.058424	1.322728	0.941637	0.839586	0.886401	1.297554
NS	4	1.306556	1.22125	0.956916	0.914097	0.926168	1.286541
NS	6	1.207502	1.081788	1.438019	1.279971	0.903208	0.951806
NS	8	1.097625	0.595039	1.532568	1.115687	1.352512	1.4352
NS	10	1.291908	1.206521	1.362443	1.616532	0.734119	1.27218
NS	24	1.351283	0.700477	1.924082	0.882567	0.915744	1.463474
Dex	0.5	1.139885	1.025316	1.175336	0.911967	0.876872	0.808047
Dex	1	1.123547	1.126876	1.009482	1.133415	0.638845	0.417732
Dex	2	1.278033	1.423958	1.000462	0.952387	0.454731	0.547884
Dex	4	1.6344	0.829459	1.404181	1.597848	0.511097	1.231273
Dex	6	1.430416	1.28725	2.447312	1.59078	0.879213	0.95993
Dex	8	1.887761	1.136594	1.741756	1.582738	0.900896	0.441888
Dex	10	1.157876	1.172009	2.524828	1.962616	0.937415	0.927813
Dex	24	1.355885	1.265818	1.967928	1.321355	0.927241	1.252219

		BIRC2					
Treatment	Time (h)	N = 1	N = 2	N = 3	N = 4	N = 5	N = 6
NS	0.5	1	1	1	1	1	1
NS	1	1.716515	1.01141	1.068936	1.973998	1.040268	1.036111
NS	2	1.976136	1.17533	1.010276	1.544049	1.124896	0.922597
NS	4	2.619151	1.008897	1.052731	1.786561	1.04448	0.724792
NS	6	2.346137	0.964574	1.164041	1.626347	1.195265	0.826908
NS	8	3.867204	1.150058	1.742731	2.773913	1.141382	1.211266
NS	10	2.375712	1.038919	1.583546	2.198256	0.834494	0.736625
NS	24	4.775938	1.023979	1.296614	2.258869	0.713652	1.12284
Bud	0.5	1.101705	1.479384	0.930744	2.178822	1.496339	0.725243
Bud	1	0.977449	1.91548	1.13552	2.093413	1.249599	0.972568
Bud	2	0.93639	1.07383	0.96398	2.03584	1.174137	0.89681
Bud	4	1.946959	1.104492	1.511701	2.590949	1.316764	0.71424
Bud	6	1.137704	1.365052	1.977751	2.596017	1.508575	0.991126
Bud	8	1.706945	1.404572	2.22874	3.114572	1.610427	0.649552
Bud	10	2.722613	1.481838	1.73983	2.817052	1.851616	0.977531
Bud	24	1.147106	0.881466	2.258021	2.492596	2.482472	1.228304

Supplemental Fig. S2B - A549 cells: mRNA

		BIRC3									
Time (h)	Treatment	N = 1	N = 2	N = 3	N = 4	N = 5	N = 6	N = 7	N = 8	N = 9	N = 10
6	NS	1	1	1	1	1	1	1	1	1	1
	Dex	4.722934	3.087961	3.086209	5.570225	10.82078	5.17778	4.038462	6.145356	4.697072	4.779609
	IL1B	52.8642	17.29925	13.3138	15.87909	65.83215	25.89006	24.42308	34.27558	21.1119	20.22077
	IL1B+Dex	45.24296	26.95055	9.701593	18.35697	51.37553	23.26704	20.5	37.94465	17.86774	20.40277
24	NS	1	1	1	1	1	1				
	Dex	3.249814	2.808021	2.879371	5.919811	4.908521	3.250256				
	IL1B	8.042803	16.71158	31.24027	36.37942	26.99591	13.2088				
	IL1B+Dex	16.34323	16.05519	13.11512	40.13528	26.20707	10.98465				

		BIRC2									
Time (h)	Treatment	N = 1	N = 2	N = 3	N = 4	N = 5	N = 6	N = 7	N = 8	N = 9	N = 10
6	NS	1	1	1	1	1	1	1	1	1	1
	Dex	1.137748	0.971849	1.24858	0.902541	1.244329	1.538348	0.763481	1.684098	1.882608	1.092766
	IL1B	2.756204	2.500793	2.363409	3.217345	2.610183	3.760261	2.922065	3.407544	2.505013	2.462744
	IL1B+Dex	1.941676	1.952284	2.020507	1.566599	1.26834	2.066548	1.480264	2.468487	1.09048	1.285832
24	NS	1	1	1	1	1	1				
	Dex	1.025588	0.969542	0.924069	1.351179	1.381769	1.184993				
	IL1B	1.250273	1.792716	1.342437	2.252258	2.066239	2.079466				
	IL1B+Dex	0.894908	1.13067	0.721663	1.368807	1.332867	1.505867				

Supplemental Fig. S2C - A549 cells: mRNA

		BIRC3										
Time (h)	Treatment	N = 1	N = 2	N = 3	N = 4	N = 5	N = 6	N = 7	N = 8	N = 9	N = 10	N = 11
6	NS	1	1	1	1	1	1	1	1	1	1	1
	Dex	4.722934	3.087961	3.086209	5.570225	10.82078	5.17778	5.219045	4.23233	2.519802	2.983822	3.749649
	TNF	44.84517	11.2332	5.789616	17.34214	24.8232	14.54421	16.1676	10.08569	9.092582	8.168695	19.72551
	TNF+Dex	66.33466	21.31984	9.472929	24.88675	30.27363	41.17368	22.66128	20.24109	26.56516	14.63005	34.08309
24	NS	1	1	1	1	1	1	1	1	1	1	1
	Dex	3.249814	2.808021	2.879371	5.919811	4.908521	3.250256	3.119562	1.030352	1.801434	2.576972	3.471219
	TNF	8.923063	9.047032	7.790541	27.5373	7.616896	10.82615	9.497019	3.309978	6.909141	6.710249	19.76287
	TNF+Dex	15.02538	16.60869	15.83099	40.21461	23.73588	12.34266	18.15756	6.411953	10.23528	11.46178	17.88202

		BIRC2										
Time (h)	Treatment	N = 1	N = 2	N = 3	N = 4	N = 5	N = 6	N = 7	N = 8	N = 9	N = 10	N = 11
6	NS	1	1	1	1	1	1	1	1	1	1	1
	Dex	1.137748	0.971849	1.24858	0.902541	1.244329	1.538348	1.462674	0.972009	0.896849	0.901292	0.782211
	TNF	3.660654	2.860539	2.675912	2.569345	3.006011	4.489556	3.414354	2.797836	3.064269	2.532493	2.56392
	TNF+Dex	2.951114	1.925826	1.578058	1.788529	1.38024	3.293069	2.505587	2.091028	1.497864	1.608401	1.997679
24	NS	1	1	1	1	1	1	1	1	1	1	1
	Dex	1.025588	0.969542	0.924069	1.351179	1.381769	1.184993	1.015805	0.71209	1.211927	1.086397	0.634794
	TNF	1.583205	1.745147	1.526411	1.873687	1.510864	2.9727	2.017257	1.419878	2.361793	2.008202	2.698106
	TNF+Dex	0.764932	1.252529	0.793651	1.679086	1.419427	1.130768	1.338405	0.930088	1.065968	1.267575	1.74688

Supplemental Fig. S2D - A549 cells: mRNA

		BIRC3					
Time (h)	Treatment	N = 1	N = 2	N = 3	N = 4	N = 5	N = 6
6	NS	1	1	1	1	1	1
	Dex	4.722934	3.087961	3.086209	5.570225	10.82078	5.17778
	IL1B	52.8642	17.29925	13.3138	15.87909	65.83215	25.89006
	IL1B+Dex	45.24296	26.95055	9.701593	18.35697	51.37553	23.26704
	IFNG	3.631448	1.490532	0.450567	1.096191	3.376008	1.688449
	IFNG+Dex	18.80679	6.738921	0.960216	6.577814	10.80532	10.69761
	IFNG+IL1B	61.70883	17.26027	14.13806	12.69044	78.85982	18.07959
	IFNG+IL1B+Dex	114.2482	19.10271	10.10403	28.81408	80.01568	29.09453
24	NS	1	1	1	1	1	1
	Dex	3.249814	2.808021	2.879371	5.919811	4.908521	3.250256
	IL1B	8.042803	16.71158	31.24027	36.37942	26.99591	13.2088
	IL1B+Dex	16.34323	16.05519	13.11512	40.13528	26.20707	10.98465
	IFNG	1.137474	4.819386	2.20847	4.738238	4.1117	2.5928
	IFNG+Dex	4.995317	11.5133	10.8349	7.950635	16.72203	9.869737
	IFNG+IL1B	18.55364	38.01183	25.72657	125.3738	58.71953	41.29402
	IFNG+IL1B+Dex	54.90076	26.56847	32.59791	80.6419	60.36568	41.89801

		BIRC2					
Time (h)	Treatment	N = 1	N = 2	N = 3	N = 4	N = 5	N = 6
6	NS	1	1	1	1	1	1
	Dex	1.137748	0.971849	1.24858	0.902541	1.244329	1.538348
	IL1B	2.756204	2.500793	2.363409	3.217345	2.610183	3.760261
	IL1B+Dex	1.941676	1.952284	2.020507	1.566599	1.26834	2.066548
	IFNG	1.907788	0.884116	1.215118	0.832983	1.477082	1.195476
	IFNG+Dex	1.606411	1.327883	1.093739	1.012272	0.858477	1.75913
	IFNG+IL1B	4.055487	2.706167	3.25354	2.505669	3.361103	2.781642
	IFNG+IL1B+Dex	3.758368	1.073701	1.91123	1.838554	2.197585	2.131752
24	NS	1	1	1	1	1	1
	Dex	1.025588	0.969542	0.924069	1.351179	1.381769	1.184993
	IL1B	1.250273	1.792716	1.342437	2.252258	2.066239	2.079466
	IL1B+Dex	0.894908	1.13067	0.721663	1.368807	1.332867	1.505867
	IFNG	0.661208	1.410883	0.8309	1.222943	1.206364	0.910255
	IFNG+Dex	0.771519	1.313779	1.147963	0.904479	1.127894	1.201424
	IFNG+IL1B	1.357499	2.165255	1.851168	2.468382	1.872507	2.913703
	IFNG+IL1B+Dex	1.324495	0.881667	0.930763	1.305633	1.095588	1.490187

Supplemental Fig. S2E - pHBEC-SC: mRNA

		BIRC3 (Fold tpm)				
Time (h)	Treatment	N = 1	N = 2	N = 3	N = 4	N = 5
2	NS	1	1	1	1	1
	Dex	3.429346	3.537215	1.609739	1.637225	4.177906
	IL1B	69.14838	18.19472	15.99084	32.19265	17.78736
	IL1B+Dex	144.2016	88.25255	92.80582	88.78642	133.7952
	IFNG	0.716739	1.757573	0.632296	0.539626	1.05645
	IFNG+Dex	1.590195	2.85789	3.163411	2.001267	1.605195
	IFNG+IL1B	35.52652	21.09091	12.54072	19.13345	18.42618
	IFNG+IL1B+Dex	91.79033	47.29928	52.23965	63.13816	53.64285
6	NS	1	1	1	1	1
	Dex	2.31778	3.00631	4.115142	1.221228	6.573967
	IL1B	7.048317	1.708988	8.527812	5.474639	16.93013
	IL1B+Dex	44.68838	25.16167	76.85478	28.65023	71.48207
	IFNG	0.565577	0.282617	0.68572	0.652708	4.397903
	IFNG+Dex	8.14037	8.924946	16.35121	4.386627	25.03773
	IFNG+IL1B	9.369318	3.898025	9.966781	10.42617	18.92364
	IFNG+IL1B+Dex	206.3983	70.76478	172.2027	72.20657	158.6862
24	NS	1	1	1	1	1
	Dex	1.531767	2.12225	0.657485	2.287064	3.520307
	IL1B	2.426444	1.638347	0.573723	5.015428	2.406777
	IL1B+Dex	7.10475	8.422668	4.324506	15.53108	17.74695
	IFNG	6.932206	1.786495	5.157274	19.89557	10.36568
	IFNG+Dex	29.5791	17.40474	57.76937	147.0451	156.9039
	IFNG+IL1B	9.550011	4.406325	7.197301	28.64994	17.303
	IFNG+IL1B+Dex	71.79112	42.37148	91.71524	207.8232	179.5744

		BIRC2 (Fold tpm)				
Time (h)	Treatment	N = 1	N = 2	N = 3	N = 4	N = 5
2	NS	1	1	1	1	1
	Dex	1.224816	1.031916	1.312101	0.682488	1.007643
	IL1B	1.184787	1.145214	1.574368	0.757063	0.923728
	IL1B+Dex	1.588795	1.253422	2.092081	0.854579	1.517246
	IFNG	1.066223	0.985315	1.067439	0.598963	0.90336
	IFNG+Dex	1.081415	1.000569	1.339916	0.651072	1.137333
	IFNG+IL1B	1.292004	1.07181	1.587335	0.833808	0.910195
	IFNG+IL1B+Dex	1.324714	1.194494	1.771672	0.84043	1.306732
6	NS	1	1	1	1	1
	Dex	1.102613	1.180493	1.20202	0.959373	1.216996
	IL1B	1.203332	0.877057	1.253399	1.18956	1.036763
	IL1B+Dex	1.41566	1.362298	1.714353	1.356906	1.43359
	IFNG	1.042184	1.024534	1.237541	1.199424	1.207221
	IFNG+Dex	1.103024	1.169047	1.547756	1.396482	1.285289
	IFNG+IL1B	1.252812	1.061641	1.363687	1.410412	1.428821
	IFNG+IL1B+Dex	1.353915	1.302731	1.925532	1.630431	1.520601
24	NS	1	1	1	1	1
	Dex	0.965676	1.112921	1.066285	1.080483	1.027083
	IL1B	1.069544	0.976229	0.997284	1.015445	1.074689
	IL1B+Dex	1.024188	1.154443	1.137011	1.082575	1.061293
	IFNG	1.309165	1.311218	1.785006	1.509889	1.261053
	IFNG+Dex	1.409565	1.469326	1.754162	1.460478	1.562023
	IFNG+IL1B	1.413724	1.365952	1.89144	1.416089	1.20295
	IFNG+IL1B+Dex	1.36551	1.551126	1.715084	1.702222	1.437678

Supplemental Fig. S3 - A549 cells: protein

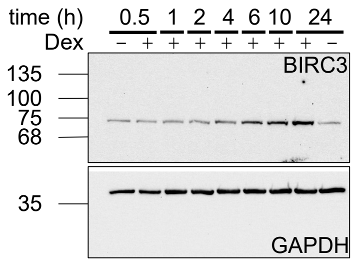

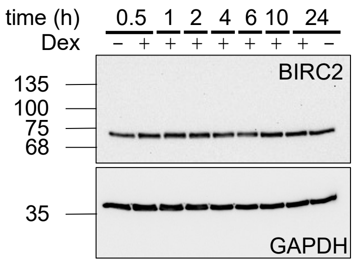

Chemiluminescence

			Adj. Total Band Vol.						Adj. Total Band Vol.				
			BIRC3						GAPDH				
Treatment	Time	Lane	N = 1	N = 2	N = 3	N = 4	N = 5	Lane	N = 1	N = 2	N = 3	N = 4	N = 5
NS	0.5	1	22017096	31705248	16889114	3966336	27340444	1	98997010	1.45E+08	1.31E+08	1.87E+08	14278104
Dex		2	17562744	36504208	42994178	6039792	44917730	2	77375584	1.45E+08	1.46E+08	1.37E+08	34315146
Dex	1	3	24103872	30324432	18386774	4106916	48506880	3	1.03E+08	80877304	1.62E+08	1.12E+08	38130320
Dex	2	4	24471072	32699159	20391284	5434506	42609600	4	99341792	81781625	1.56E+08	1.63E+08	17510590
Dex	4	5	36863856	37039408	49104282	6408864	40965120	5	98315184	72187670	1.6E+08	1.01E+08	13976410
Dex	6	6	62184456	54853094	49495592	8586468	52675008	6	1.18E+08	78929370	1.49E+08	1.57E+08	26180440
Dex	10	7	80023464	51954986	59792604	13105818	93110592	7	1.18E+08	76526730	1.45E+08	1.79E+08	31416850
Dex	24	8	1.72E+08	82384897	1.8E+08	24772374	1.37E+08	8	1.12E+08	71949049	1.44E+08	1.52E+08	46300044
NS		9	20938713	36054417	24172930	4671612	37773648	9	1.65E+08	2.15E+08	1.37E+08	2.2E+08	87114872

			Normalized Values				
			BIRC3				
Treatment	Time	Lane	N = 1	N = 2	N = 3	N = 4	N = 5
NS	0.5	1	0.222402	0.217988	0.129324	0.021246	1.914851
Dex		2	0.22698	0.252372	0.294396	0.043957	1.308977
Dex	1	3	0.235143	0.374944	0.113791	0.036795	1.272134
Dex	2	4	0.246332	0.399835	0.131117	0.033359	2.433362
Dex	4	5	0.374956	0.513099	0.307116	0.063657	2.931019
Dex	6	6	0.525976	0.694964	0.332257	0.054635	2.011999
Dex	10	7	0.676848	0.678913	0.41278	0.073368	2.963715
Dex	24	8	1.533282	1.145045	1.248572	0.162678	2.948796
NS		9	0.126827	0.167436	0.17702	0.021255	0.433607

			Adj. Total Band Vol.						Adj. Total Band Vol.				
			BIRC2						GAPDH				
Treatment	Time	Lane	N = 1	N = 2	N = 3	N = 4	N = 5	Lane	N = 1	N = 2	N = 3	N = 4	N = 5
NS	0.5	1	44636184	6.4E+07	3.7E+07	63898164	1.2E+08	1	1.74E+08	1.3E+08	3.8E+08	1.9E+08	3.56E+08
Dex		2	48469536	1.2E+08	2.4E+07	67557996	1.4E+08	2	1.95E+08	1.5E+08	2.8E+08	2.74E+08	3.45E+08
Dex	1	3	81775008	6.6E+07	3.6E+07	58763232	1.5E+08	3	1.86E+08	1.1E+08	3.8E+08	1.65E+08	2.85E+08
Dex	2	4	42647040	7.6E+07	3.7E+07	62322282	1.5E+08	4	2.19E+08	1.2E+08	4.3E+08	1.3E+08	2.51E+08
Dex	4	5	41134608	6.9E+07	4.7E+07	84461850	1.2E+08	5	1.62E+08	1.3E+08	4.5E+08	1.8E+08	2.54E+08
Dex	6	6	39842712	3.6E+07	1.7E+07	83986848	9.9E+07	6	1.59E+08	1E+08	3.7E+08	1.53E+08	2.71E+08
Dex	10	7	36683280	1E+08	3.1E+07	58734720	1.8E+08	7	2.17E+08	1.2E+08	3.5E+08	1.78E+08	3.17E+08
Dex	24	8	40304304	3.9E+07	1.2E+07	71215056	1.8E+08	8	1.82E+08	1E+08	3.3E+08	2.16E+08	3.64E+08
NS		9	50251347	1.3E+08	1.8E+07	70028070	1.6E+08	9	1.76E+08	2E+08	3.5E+08	1.43E+08	3.89E+08

			Normalized Values				
			BIRC2				
Treatment	Time	Lane	N = 1	N = 2	N = 3	N = 4	N = 5
NS	0.5	1	0.257114	0.506673	0.09846	0.336411	0.328545
Dex		2	0.24804	0.818014	0.084344	0.246403	0.415051
Dex	1	3	0.438798	0.617306	0.095424	0.356908	0.532523
Dex	2	4	0.19448	0.632987	0.086201	0.478376	0.58261
Dex	4	5	0.253632	0.536176	0.103658	0.469954	0.48443
Dex	6	6	0.250264	0.358586	0.046497	0.548977	0.366068
Dex	10	7	0.169303	0.806486	0.088897	0.329757	0.562458
Dex	24	8	0.221005	0.387399	0.037436	0.329893	0.492927
NS		9	0.28503	0.653368	0.052997	0.488286	0.419458

Supplemental Fig. S3 - A549 cells: protein

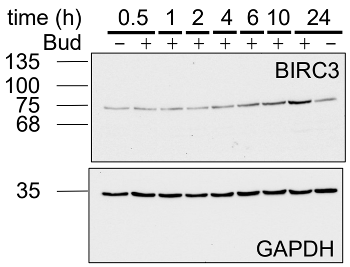

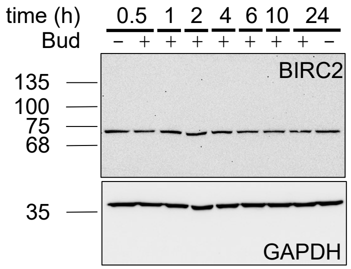

Chemiluminescence

			Adj. Total Band Vol.						Adj. Total Band Vol.								Normalized Values				
			BIRC3						GAPDH								BIRC3				
Treatment	Time	Lane	N = 1	N = 2	N = 3	N = 4	N = 5	Lane	N = 1	N = 2	N = 3	N = 4	N = 5	Treatment	Time	Lane	N = 1	N = 2	N = 3	N = 4	N = 5
NS	0.5	1	23343501	54460120	29025389	1043900	14789171	1	1.99E+08	1.46E+08	1.75E+08	51186911	1.29E+08	NS	0.5	1	0.117014	0.373088	0.166125	0.020394	0.114301
BUD		2	23542038	63129221	38043710	2333650	16569416	2	1.37E+08	1.6E+08	1.96E+08	1E+08	1.73E+08	BUD		2	0.171963	0.394684	0.193837	0.023285	0.09591
BUD	1	3	22089834	38013360	41943566	1408990	18158422	3	1.87E+08	53686425	2E+08	61049834	1.57E+08	BUD	1	3	0.118115	0.708063	0.209371	0.023079	0.115564
BUD	2	4	21215376	45759483	36201358	2019050	13541920	4	1.72E+08	84492159	1.51E+08	83992043	1.07E+08	BUD	2	4	0.12308	0.541583	0.239674	0.024039	0.126952
BUD	4	5	30375072	56075943	48159018	5848810	23153022	5	1.6E+08	79635318	1.77E+08	1.22E+08	1.42E+08	BUD	4	5	0.190116	0.704159	0.27235	0.047924	0.162822
BUD	6	6	56138706	51151716	63803190	6127110	37387632	6	2.19E+08	40377690	1.87E+08	95939961	1.84E+08	BUD	6	6	0.256736	1.266831	0.341852	0.063864	0.20359
BUD	10	7	79341950	1.11E+08	74259532	13037200	54072760	7	2.19E+08	73582812	1.67E+08	95083944	1.57E+08	BUD	10	7	0.362839	1.503813	0.444929	0.137113	0.344142
BUD	24	8	1.91E+08	1.2E+08	2.23E+08	28760710	97026546	8	1.74E+08	89229315	2.17E+08	1.92E+08	1.54E+08	BUD	24	8	1.096936	1.345827	1.028893	0.149736	0.629538
NS		9	11122845	23831256	43537848	1658910	25207320	9	2.63E+08	63319644	2.15E+08	1.1E+08	2.01E+08	NS		9	0.042293	0.376364	0.202716	0.015103	0.125599

			Adj. Total Band Vol.						Adj. Total Band Vol.								Normalized Values				
			BIRC2						GAPDH								BIRC2				
Treatment	Time	Lane	N = 1	N = 2	N = 3	N = 4	N = 5	Lane	N = 1	N = 2	N = 3	N = 4	N = 5	Treatment	Time	Lane	N = 1	N = 2	N = 3	N = 4	N = 5
NS	0.5	1	1.52E+08	79943792	24575600	1.01E+08	1.36E+08	1	1.25E+08	1.13E+08	2.82E+08	3.42E+08	129168904	NS	0.5	1	1.211435	0.709603	0.087227	0.295275	1.053901
BUD		2	1.41E+08	1.04E+08	13835650	1.08E+08	1.7E+08	2	91165239	1.21E+08	2.32E+08	3.06E+08	165197264	BUD		2	1.542556	0.861028	0.059723	0.352672	1.026615
BUD	1	3	1.96E+08	52513128	24444860	79541378	1.29E+08	3	1.21E+08	54017976	2.33E+08	3.09E+08	123433568	BUD	1	3	1.616287	0.972142	0.104757	0.25755	1.0489
BUD	2	4	1.5E+08	65970450	22229960	69842552	1.42E+08	4	97450519	81584502	2.22E+08	2.64E+08	99084680	BUD	2	4	1.537501	0.808615	0.100121	0.264698	1.431847
BUD	4	5	1.7E+08	53315028	21471420	64369772	1.36E+08	5	82180277	96912282	2.23E+08	2.5E+08	80458633	BUD	4	5	2.065685	0.550137	0.096301	0.256975	1.695226
BUD	6	6	1.95E+08	33331662	14326700	69940708	1.34E+08	6	1.2E+08	53811576	2.52E+08	2.45E+08	98998462	BUD	6	6	1.627975	0.619414	0.056845	0.285726	1.354032
BUD	10	7	1.93E+08	59610250	13536880	66947904	1.81E+08	7	99687520	77475852	2.55E+08	2.4E+08	165280200	BUD	10	7	1.933082	0.769404	0.053017	0.279031	1.092934
BUD	24	8	2.03E+08	54698130	14064960	1.43E+08	1.83E+08	8	85129153	1.43E+08	2.54E+08	3.05E+08	189771044	BUD	24	8	2.37966	0.383428	0.055374	0.469405	0.96213
NS		9	2.24E+08	29936235	20739810	87668870	2.54E+08	9	1.32E+08	1.56E+08	2.64E+08	2.88E+08	222508620	NS		9	1.689045	0.191389	0.078655	0.304224	1.142187

Supplemental Fig. S3 - A549 cells: protein

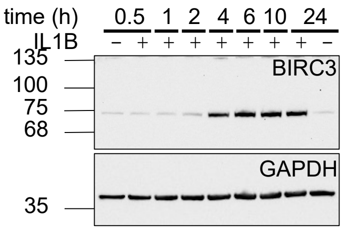

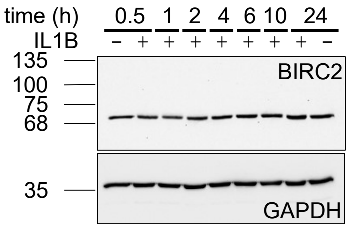

Chemiluminescence

			Adj. Total Band Vol.				
			BIRC3				
Treatment	Time	Lane	N = 1	N = 2	N = 3	N = 4	N = 5
NS	0.5	1	4376452	6678700	1.5E+07	3774498	2E+07
IL1B		2	3693072	1E+07	2.2E+07	3798864	2.8E+07
IL1B	1	3	4617092	8712592	1.1E+07	3805002	2.7E+07
IL1B	2	4	8613784	1.7E+07	2E+07	1E+07	5.9E+07
IL1B	4	5	7.2E+07	7.7E+07	1.2E+08	2.9E+07	1.1E+08
IL1B	6	6	1.3E+08	1.1E+08	1.7E+08	5.3E+07	1.4E+08
IL1B	10	7	1.3E+08	9E+07	1.8E+08	4.9E+07	2.2E+08
IL1B	24	8	1.2E+08	1.5E+08	2.1E+08	6.8E+07	2.9E+08
NS		9	3500184	6178704	1.5E+07	3233982	3.3E+07

	Adj. Total Band Vol.				
	GAPDH				
Lane	N = 1	N = 2	N = 3	N = 4	N = 5
1	2E+08	1.4E+08	2E+08	1.8E+08	2.7E+08
2	1.9E+08	1.4E+08	1.3E+08	1.9E+08	2.2E+08
3	1.7E+08	1.1E+08	1.4E+08	1.2E+08	2.2E+08
4	1.8E+08	1.2E+08	1.2E+08	1.2E+08	2.1E+08
5	1.8E+08	1.1E+08	1.2E+08	1.1E+08	2.1E+08
6	1.9E+08	1.2E+08	1.1E+08	1.1E+08	2.3E+08
7	1.8E+08	1.2E+08	9.9E+07	1E+08	2.6E+08
8	1.7E+08	1.3E+08	2E+08	1E+08	2.7E+08
9	2.4E+08	1.6E+08	3E+08	1.2E+08	3.4E+08

			Normalized Values				
			BIRC3				
Treatment	Time	Lane	N = 1	N = 2	N = 3	N = 4	N = 5
NS	0.5	1	0.021934	0.047268	0.076403	0.02077	0.075976
IL1B		2	0.019579	0.075717	0.171619	0.019822	0.124483
IL1B	1	3	0.027289	0.076595	0.08043	0.032258	0.119286
IL1B	2	4	0.046916	0.136258	0.159788	0.087372	0.278146
IL1B	4	5	0.396515	0.730509	1.004001	0.254123	0.534171
IL1B	6	6	0.674148	0.861026	1.514961	0.460682	0.618081
IL1B	10	7	0.743375	0.762566	1.79354	0.484047	0.854586
IL1B	24	8	0.714297	1.153681	1.058337	0.660865	1.087788
NS		9	0.014609	0.039097	0.049655	0.026298	0.097016

			Adj. Total Band Vol.				
			BIRC2				
Treatment	Time	Lane	N = 1	N = 2	N = 3	N = 4	N = 5
NS	0.5	1	76965750	25892304	42592000	89618410	1E+08
IL1B		2	63619600	13502658	15682733	1.3E+08	7.1E+07
IL1B	1	3	55593164	9391837	34163140	98590932	6.8E+07
IL1B	2	4	73875439	20424157	46891658	1.47E+08	8.3E+07
IL1B	4	5	85861171	15971228	72357472	1.75E+08	1.8E+08
IL1B	6	6	1.08E+08	10535954	62801574	1.96E+08	1.7E+08
IL1B	10	7	97720876	28717403	46366023	1.65E+08	1.9E+08
IL1B	24	8	1.27E+08	31383989	39293254	1.09E+08	1.7E+08
NS		9	1.17E+08	19039813	28121937	52558704	5.1E+07

	Adj. Total Band Vol.				
	GAPDH				
Lane	N = 1	N = 2	N = 3	N = 4	N = 5
1	99671325	1.41E+08	1.13E+08	1.53E+08	67242616
2	97475061	1.35E+08	60456632	1.52E+08	53282327
3	1E+08	1.14E+08	83054844	1.35E+08	56043540
4	1.06E+08	1.23E+08	1E+08	1.43E+08	59139913
5	1.08E+08	1.05E+08	90727593	1.39E+08	69048147
6	1.03E+08	1.22E+08	1.1E+08	1.5E+08	74125156
7	86996312	1.18E+08	1.2E+08	1.42E+08	63685321
8	97701670	1.33E+08	1.06E+08	1.34E+08	68773875
9	1.12E+08	1.58E+08	1.13E+08	90752145	69547000

			Normalized Values				
			BIRC2				
Treatment	Time	Lane	N = 1	N = 2	N = 3	N = 4	N = 5
NS	0.5	1	0.772196	0.18325	0.37695	0.587491	1.505731
IL1B		2	0.652676	0.099748	0.259405	0.858224	1.341585
IL1B	1	3	0.554547	0.082566	0.411332	0.7296	1.204805
IL1B	2	4	0.697815	0.166673	0.467418	1.029502	1.400306
IL1B	4	5	0.797832	0.151434	0.797524	1.259023	2.566529
IL1B	6	6	1.052535	0.086061	0.572597	1.313039	2.236083
IL1B	10	7	1.123276	0.242633	0.387791	1.163114	3.019768
IL1B	24	8	1.296565	0.236006	0.370983	0.808963	2.515015
NS		9	1.048091	0.120478	0.249744	0.579146	0.727138

Supplemental Fig. S3 - A549 cells: protein

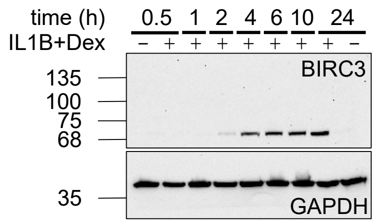

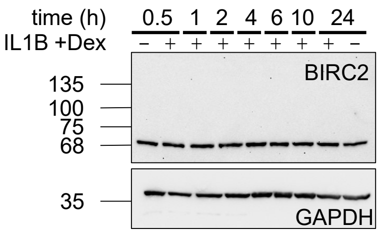

Chemiluminescence

			Adj. Total Band Vol.						Adj. Total Band Vol.								Normalized Values				
			BIRC3						GAPDH								BIRC3				
Treatment	Time	Lane	N = 1	N = 2	N = 3	N = 4	N = 5	Lane	N = 1	N = 2	N = 3	N = 4	N = 5	Treatment	Time	Lane	N = 1	N = 2	N = 3	N = 4	N = 5
NS	0.5	1	1082796	1055054	1105456	15642	5451	1	4.7E+08	4.53E+08	1.03E+08	2.01E+08	2.02E+08	NS	0.5	1	0.002305	0.00233	0.010715	7.79E-05	2.7E-05
IL1B+DEX		2	487756	1038708	757856	13272	16116	2	3.4E+08	4.35E+08	72136080	1.6E+08	1.55E+08	IL1B+DEX		2	0.001433	0.002389	0.010506	8.32E-05	0.000104
IL1B+DEX	1	3	703922	442976	1390048	26307	45741	3	4.2E+08	4.46E+08	1.08E+08	1.89E+08	2.07E+08	IL1B+DEX	1	3	0.001675	0.000993	0.012861	0.000139	0.000221
IL1B+DEX	2	4	4209141	927213	1835504	357159	86742	4	3.76E+08	3.7E+08	81534960	2.18E+08	2.2E+08	IL1B+DEX	2	4	0.011193	0.002505	0.022512	0.001638	0.000394
IL1B+DEX	4	5	43301250	27900450	8612920	5.6E+07	18914496	5	4.3E+08	3.9E+08	94586160	2.38E+08	2.02E+08	IL1B+DEX	4	5	0.100686	0.071476	0.091059	0.233886	0.09357
IL1B+DEX	6	6	58363254	58999756	2.4E+07	1.9E+08	79063560	6	4.57E+08	3.43E+08	1.17E+08	1.84E+08	1.75E+08	IL1B+DEX	6	6	0.127734	0.171965	0.206273	1.05276	0.45106
IL1B+DEX	10	7	66241350	70186500	2.5E+07	1.8E+08	88048125	7	4.74E+08	3.63E+08	1.05E+08	1.41E+08	1.85E+08	IL1B+DEX	10	7	0.139774	0.1932	0.24297	1.289695	0.474941
IL1B+DEX	24	8	1.35E+08	1.02E+08	2.7E+07	2E+08	1.9E+08	8	4.63E+08	3.91E+08	84001400	1.23E+08	1.52E+08	IL1B+DEX	24	8	0.291045	0.260318	0.316436	1.656598	1.248669
NS		9	1001925	972000	642524	90060	13272	9	5.15E+08	4.91E+08	1.29E+08	2.05E+08	1.68E+08	NS		9	0.001946	0.001979	0.004995	0.000439	7.92E-05

			Adj. Total Band Vol.						Adj. Total Band Vol.								Normalized Values				
			BIRC2						GAPDH								BIRC2				
Treatment	Time	Lane	N = 1	N = 2	N = 3	N = 4	N = 5	Lane	N = 1	N = 2	N = 3	N = 4	N = 5	Treatment	Time	Lane	N = 1	N = 2	N = 3	N = 4	N = 5
NS	0.5	1	1.63E+08	2.48E+08	2.7E+07	1.1E+08	6.5E+07	1	2.06E+08	3.13E+08	1.5E+08	1.9E+08	1.95E+08	NS	0.5	1	0.792221	0.790352	0.177066	0.573643	0.330823
IL1B+DEX		2	1.03E+08	2.58E+08	1.1E+07	9.9E+07	5.4E+07	2	1.58E+08	3.1E+08	1.1E+08	1.6E+08	1.49E+08	IL1B+DEX		2	0.650471	0.831345	0.101024	0.629271	0.362087
IL1B+DEX	1	3	1.76E+08	2.61E+08	3.2E+07	1E+08	6.5E+07	3	1.92E+08	3.73E+08	1.7E+08	1.7E+08	1.98E+08	IL1B+DEX	1	3	0.913944	0.700567	0.18763	0.624646	0.326504
IL1B+DEX	2	4	1.72E+08	2.46E+08	3.9E+07	1E+08	6.1E+07	4	1.84E+08	3.02E+08	1.8E+08	1.5E+08	2.2E+08	IL1B+DEX	2	4	0.933401	0.816109	0.213979	0.697147	0.275916
IL1B+DEX	4	5	2.21E+08	2.64E+08	4.4E+07	1.3E+08	9.3E+07	5	1.69E+08	3.06E+08	1.4E+08	1.4E+08	1.98E+08	IL1B+DEX	4	5	1.305098	0.862101	0.305433	0.914211	0.468734
IL1B+DEX	6	6	2.24E+08	2.74E+08	5.1E+07	1.1E+08	7.2E+07	6	1.9E+08	4.42E+08	1.3E+08	1.7E+08	1.72E+08	IL1B+DEX	6	6	1.178515	0.619689	0.403027	0.62279	0.418768
IL1B+DEX	10	7	2.15E+08	2.65E+08	3.8E+07	1.3E+08	6.6E+07	7	1.96E+08	3.08E+08	1.4E+08	1.8E+08	1.82E+08	IL1B+DEX	10	7	1.095713	0.859753	0.26623	0.71021	0.362637
IL1B+DEX	24	8	1.59E+08	2.24E+08	2.5E+07	1.1E+08	7.1E+07	8	1.52E+08	2.9E+08	1.5E+08	1.5E+08	1.46E+08	IL1B+DEX	24	8	1.042189	0.772323	0.165077	0.690783	0.483243
NS		9	2.13E+08	99176580	9.3E+07	1.7E+08	4.1E+07	9	2.64E+08	1.92E+08	2.3E+08	1.7E+08	1.6E+08	NS		9	0.807892	0.516431	0.405799	1.009861	0.257745

Supplemental Fig. S3 - A549 cells: protein

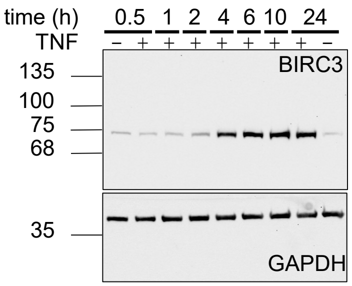

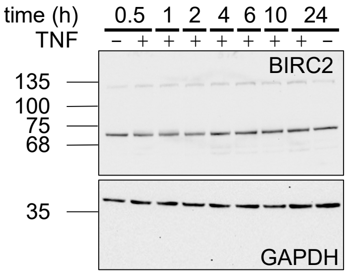

Chemiluminescence

			Adj. Total Band Vol.						Adj. Total Band Vol.				
			BIRC3						GAPDH				
Treatment	Time	Lane	N = 1	N = 2	N = 3	N = 4	N = 5	Lane	N = 1	N = 2	N = 3	N = 4	N = 5
NS	0.5	1	8179542	22772972	7880928	32383256	12223856	1	1.67E+08	2.02E+08	1.16E+08	63787020	3.17E+08
TNF		2	6004152	20976948	5695712	22062040	12828285	2	1.48E+08	1.85E+08	1.24E+08	56395240	3E+08
TNF	1	3	7852194	21492260	3395920	16131888	11186874	3	1.66E+08	2E+08	1.33E+08	27468980	3.21E+08
TNF	2	4	15248331	16164044	7130640	27631664	26589320	4	1.92E+08	1.36E+08	1.18E+08	56949640	3.24E+08
TNF	4	5	77544054	79464860	84019936	86861264	57009065	5	1.47E+08	1.21E+08	1.56E+08	67200760	3.25E+08
TNF	6	6	1.37E+08	1.44E+08	1.33E+08	96153792	61304430	6	1.64E+08	1.6E+08	1.19E+08	49234900	2.95E+08
TNF	10	7	1.62E+08	1.42E+08	1.42E+08	1.38E+08	1.03E+08	7	2.02E+08	1.34E+08	1.47E+08	1.16E+08	3.83E+08
TNF	24	8	1.33E+08	1.78E+08	1.69E+08	1.59E+08	82394151	8	1.24E+08	1.28E+08	1.78E+08	1.9E+08	3.12E+08
NS		9	5383854	12313652	6768256	20801844	17004000	9	1.71E+08	1.69E+08	1.37E+08	1.66E+08	3.45E+08

			Normalized Values				
			BIRC3				
Treatment	Time	Lane	N = 1	N = 2	N = 3	N = 4	N = 5
NS	0.5	1	0.048888	0.11284	0.068103	0.507678	0.038587
TNF		2	0.040637	0.113219	0.046045	0.391204	0.042723
TNF	1	3	0.047253	0.107467	0.025545	0.587277	0.034856
TNF	2	4	0.079516	0.118705	0.060379	0.485195	0.082015
TNF	4	5	0.528723	0.657053	0.536946	1.292564	0.175195
TNF	6	6	0.83496	0.897119	1.11434	1.95296	0.207661
TNF	10	7	0.803436	1.056293	0.969047	1.189202	0.269617
TNF	24	8	1.071603	1.392534	0.948593	0.83319	0.264012
NS		9	0.031459	0.07269	0.049259	0.125492	0.049345

			Adj. Total Band Vol.						Adj. Total Band Vol.				
			BIRC2						GAPDH				
Treatment	Time	Lane	N = 1	N = 2	N = 3	N = 4	N = 5	Lane	N = 1	N = 2	N = 3	N = 4	N = 5
NS	0.5	1	26869140	72971820	70697060	1.5E+08	2.38E+08	1	2.85E+08	3.45E+08	3.29E+08	76487400	138826625
TNF		2	13145580	65591505	34848910	1.2E+08	1.54E+08	2	3.57E+08	2.57E+08	2.89E+08	1.06E+08	129224656
TNF	1	3	13722300	27199431	32889540	1.36E+08	1.28E+08	3	3.29E+08	2.27E+08	3.19E+08	1.19E+08	114856268
TNF	2	4	14354280	22947967	30928560	1.14E+08	1.28E+08	4	3.05E+08	2.23E+08	3.16E+08	1.01E+08	122406930
TNF	4	5	21608280	33968746	47589760	1.55E+08	1.5E+08	5	2.82E+08	2.31E+08	2.96E+08	1.19E+08	111047336
TNF	6	6	32810580	31950863	53891990	1.79E+08	2.46E+08	6	3.66E+08	2.18E+08	3.19E+08	1.4E+08	150647056
TNF	10	7	31381560	42799778	41046490	1.76E+08	2.74E+08	7	3.59E+08	2.32E+08	2.78E+08	83593400	155094500
TNF	24	8	32088960	44314438	46729330	1.81E+08	2.43E+08	8	3.8E+08	2.47E+08	3.02E+08	1.63E+08	137083000
NS		9	39947400	1.9E+08	53883648	1.72E+08	2.56E+08	9	4.09E+08	3.73E+08	3E+08	2.24E+08	127766016

			Normalized Values				
			BIRC2				
Treatment	Time	Lane	N = 1	N = 2	N = 3	N = 4	N = 5
NS	0.5	1	0.094276	0.211751	0.215172	1.95658	1.711548
TNF		2	0.036839	0.254969	0.120469	1.133062	1.187889
TNF	1	3	0.041739	0.119558	0.103159	1.145376	1.115421
TNF	2	4	0.047127	0.102987	0.097724	1.130119	1.048045
TNF	4	5	0.076611	0.146902	0.160696	1.303231	1.348551
TNF	6	6	0.089741	0.14656	0.168895	1.276791	1.630189
TNF	10	7	0.087515	0.184657	0.147568	2.105015	1.767561
TNF	24	8	0.08455	0.179229	0.154584	1.107577	1.773952
NS		9	0.09765	0.509346	0.179475	0.767785	2.002433

Supplemental Fig. S3 - A549 cells: protein

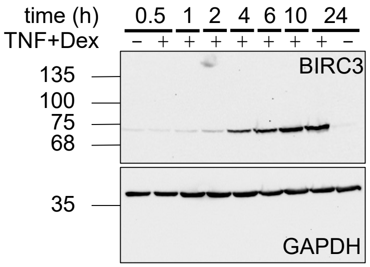

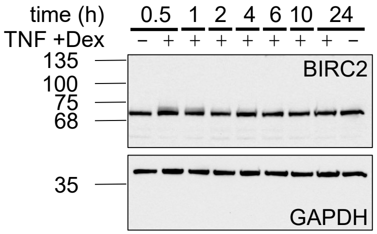

Chemiluminescence

			Adj. Total Band Vol.				
			BIRC3				
Treatment	Time	Lane	N = 1	N = 2	N = 3	N = 4	N = 5
NS	0.5	1	2798400	3563035	7640830	14677530	12629765
TNF+DEX		2	2464880	4353720	3799958	17675700	16658180
TNF+DEX	1	3	4235660	2158902	3369695	10597860	18698240
TNF+DEX	2	4	9460880	2310231	5952705	22598310	19733340
TNF+DEX	4	5	65401160	8058105	42954420	49513380	57473240
TNF+DEX	6	6	1.04E+08	15294522	75458980	66224340	1.1E+08
TNF+DEX	10	7	2.2E+08	18834876	1.62E+08	1.08E+08	1.54E+08
TNF+DEX	24	8	2.42E+08	27888774	1.76E+08	1.65E+08	2.52E+08
NS		9	2888820	355218	3585011	11050340	24844600

	Adj. Total Band Vol.				
	GAPDH				
Lane	N = 1	N = 2	N = 3	N = 4	N = 5
1	1.76E+08	1.88E+08	1.32E+08	1.5E+08	1.64E+08
2	1.47E+08	1.81E+08	1.4E+08	1.7E+08	2.21E+08
3	1.74E+08	1.17E+08	1.63E+08	1.06E+08	1.44E+08
4	1.84E+08	1.44E+08	1.57E+08	1.29E+08	1.87E+08
5	1.76E+08	1.52E+08	1.99E+08	1.43E+08	1.57E+08
6	1.27E+08	1.16E+08	1.76E+08	1.13E+08	1.38E+08
7	1.34E+08	1.43E+08	1.62E+08	1.12E+08	1.79E+08
8	1.3E+08	1.45E+08	1.42E+08	1.41E+08	1.65E+08
9	1.32E+08	1.74E+08	1.26E+08	1.42E+08	1.94E+08

			Normalized Values				
			BIRC3				
Treatment	Time	Lane	N = 1	N = 2	N = 3	N = 4	N = 5
NS	0.5	1	0.015866	0.018979	0.057875	0.097843	0.07695
TNF+DEX		2	0.016815	0.024032	0.027227	0.103766	0.07525
TNF+DEX	1	3	0.024347	0.018515	0.020614	0.099963	0.130002
TNF+DEX	2	4	0.051292	0.016095	0.037911	0.174567	0.105593
TNF+DEX	4	5	0.372271	0.052913	0.215452	0.345229	0.367238
TNF+DEX	6	6	0.823876	0.132082	0.428683	0.588176	0.793911
TNF+DEX	10	7	1.640745	0.131847	1.001726	0.968587	0.858201
TNF+DEX	24	8	1.864216	0.192515	1.240033	1.1681	1.530754
NS		9	0.021958	0.002047	0.028471	0.078006	0.128239

			Adj. Total Band Vol.				
			BIRC2				
Treatment	Time	Lane	N = 1	N = 2	N = 3	N = 4	N = 5
NS	0.5	1	60230137	1.28E+08	4.4E+07	1.2E+08	57919176
TNF+DEX		2	61893936	1.24E+08	3.5E+07	1.39E+08	67760203
TNF+DEX	1	3	1.7E+08	64692276	5.9E+07	86444424	72358992
TNF+DEX	2	4	1.76E+08	56089884	5.1E+07	85216824	58150505
TNF+DEX	4	5	2.16E+08	64391728	5.8E+07	1.29E+08	70446072
TNF+DEX	6	6	1.86E+08	73545503	3.5E+07	1.15E+08	80588632
TNF+DEX	10	7	2.15E+08	54410084	1.9E+07	1.03E+08	95694525
TNF+DEX	24	8	1.84E+08	41375063	1.4E+07	1.13E+08	96873612
NS		9	1.99E+08	1.14E+08	3E+07	1.51E+08	1.6E+08

	Adj. Total Band Vol.				
	GAPDH				
Lane	N = 1	N = 2	N = 3	N = 4	N = 5
1	85502774	98304038	2.3E+08	2.44E+08	58406588
2	85157000	1.19E+08	1.8E+08	2.93E+08	94496152
3	1.41E+08	97148831	1.9E+08	2.34E+08	105974352
4	1.07E+08	69120688	1.7E+08	2.3E+08	76882712
5	1.14E+08	85084273	1.7E+08	2.31E+08	93450350
6	1.28E+08	96617296	1.4E+08	2.14E+08	95990928
7	1.03E+08	72381306	1.4E+08	1.91E+08	77230480
8	1.25E+08	94973934	1.6E+08	2.51E+08	88989632
9	1.16E+08	1.2E+08	2.1E+08	2.3E+08	86065040

			Normalized Values				
			BIRC2				
Treatment	Time	Lane	N = 1	N = 2	N = 3	N = 4	N = 5
NS	0.5	1	0.704423	1.30137	0.189893	0.49138	0.991655
TNF+DEX		2	0.726821	1.041632	0.19574	0.474815	0.717068
TNF+DEX	1	3	1.203769	0.665909	0.319675	0.370143	0.682797
TNF+DEX	2	4	1.64739	0.811478	0.310158	0.370494	0.756353
TNF+DEX	4	5	1.885281	0.756799	0.335806	0.560031	0.753834
TNF+DEX	6	6	1.448504	0.761204	0.242655	0.539622	0.839544
TNF+DEX	10	7	2.086117	0.751715	0.134116	0.536591	1.239077
TNF+DEX	24	8	1.480743	0.435647	0.083741	0.447887	1.088594
NS		9	1.72122	0.948281	0.140671	0.655249	1.854229

Supplemental Fig. S4 - A549 cells: protein

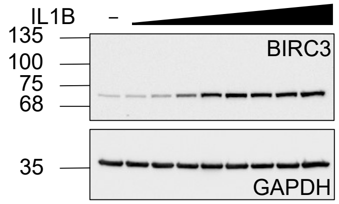

Chemiluminescence

			Adj. Total Band Vol.									Adj. Total Band Vol.							
			BIRC3									GAPDH							
Stim	Concentration (ng/mL)	Lane	N = 1	N = 2	N = 3	N = 4	N = 5	N = 6	N = 7	N = 8	Lane	N = 1	N = 2	N = 3	N = 4	N = 5	N = 6	N = 7	N = 8
NS	-	1	933009	162125	73860	2634309	7563633	1987225	708290	3605360	1	44638560	23466777	97131000	58459554	279549860	74806038	30398108	1.21E+08
IL1B	-2.5	2	1181635	350375	95440	3308557	44104821	3109525	1678733	3427380	2	28539840	55600935	1.29E+08	58213755	251677424	81588156	30150412	64431234
IL1B	-2	3	1753465	347750	133360	5183640	3026218	4099005	2913358	15804800	3	43720416	33609918	1.43E+08	54087120	217619072	1.05E+08	32848901	66918753
IL1B	-1.5	4	3794658	521500	227820	13924584	4519012	6901275	7380618	81858260	4	42736512	15260571	98439800	52354008	212553042	87860634	32671688	1.13E+08
IL1B	-1	5	9941463	2637500	595460	31918252	52206443	9900455	12859735	1.15E+08	5	36756192	28730493	1.12E+08	58417392	256473452	83575752	32612619	1.11E+08
IL1B	-0.5	6	9205434	6300000	2307120	53743180	87014881	13477376	11243477	1.23E+08	6	54504192	40000449	1.25E+08	56288978	327746106	99474288	35659045	98215911
IL1B	0	7	14722834	3247375	2233720	45564420	88696724	12530632	11697439	1.37E+08	7	45195494	18672492	90098400	61831506	290264088	88139634	35160077	1.02E+08
IL1B	0.5	8	13133617	4602625	2735780	53685622	84817276	13979740	11961761	1.27E+08	8	47615328	34045809	1.48E+08	62588787	296471669	70439688	35347021	1.02E+08
IL1B	1	9	13995527	3527875	2237960	66803037	1.08E+08	13769055	10406831	1.09E+08	9	59127552	37109430	1.38E+08	85001130	365237061	66324810	37039394	88108929

			Fold of NS							
			BIRC3							
Stim	Concentration (ng/mL)	Lane	N = 1	N = 2	N = 3	N = 4	N = 5	N = 6	N = 7	N = 8
NS	-	1	1	1	1	1	1	1	1	1
IL1B	-2.5	2	1.980871	0.912125	0.9748	1.261252	6.476951	1.434685	2.389592	1.792368
IL1B	-2	3	1.918833	1.497625	1.225426	2.126815	0.513963	1.473512	3.806348	7.957976
IL1B	-1.5	4	4.248131	4.946374	3.043475	5.902297	0.785787	2.956818	9.695196	24.42423
IL1B	-1	5	12.94029	13.28779	7.011664	12.12511	7.523337	4.459277	16.92317	35.18544
IL1B	-0.5	6	8.080509	22.79708	24.35948	21.18794	9.812616	5.100164	13.53214	42.32849
IL1B	0	7	15.5855	25.17292	32.6032	16.35328	11.29388	5.351695	14.2783	45.29894
IL1B	0.5	8	13.1966	19.56795	24.2603	19.03488	10.57377	7.470872	14.52372	41.97303
IL1B	1	9	11.32462	13.76044	21.38519	17.44055	10.94403	7.814798	12.05841	41.83215

Supplemental Fig. S4 - A549 cells: protein

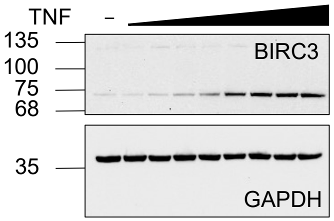

Chemiluminescence

			Adj. Total Band Vol.									Adj. Total Band Vol.							
			BIRC3									GAPDH							
Stim	Concentration (ng/mL)	Lane	N = 1	N = 2	N = 3	N = 4	N = 5	N = 6	N = 7	N = 8	Lane	N = 1	N = 2	N = 3	N = 4	N = 5	N = 6	N = 7	N = 8
NS	-	1	2774458	519138	3218160	1417900	4688250	3981704	674288	3466980	1	1.69E+08	58919100	2.24E+08	70298197	374062882	2.21E+08	1.25E+08	75693276
TNF	-1.5	2	5280465	562304	17261860	4065600	2443060	16493139	543906	5377020	2	1.03E+08	1.23E+08	3.04E+08	62086112	363690005	2.33E+08	1.08E+08	86639436
TNF	-1	3	23560207	1433455	22034540	3012020	1539860	15085966	1577984	14395920	3	2.63E+08	2.04E+08	2.68E+08	33707178	244505126	2.56E+08	1.22E+08	84119418
TNF	-0.5	4	32948231	8151116	66838420	7018440	2221425	39445336	2588880	40284200	4	2.07E+08	1.39E+08	3.1E+08	33711838	284521852	2.15E+08	1.33E+08	1.01E+08
TNF	0	5	83396721	22190571	1.08E+08	12596320	4519944	27467227	2368182	59324760	5	1.47E+08	1.55E+08	3.21E+08	53954645	301110842	2.16E+08	96127776	83662569
TNF	0.5	6	1.74E+08	50866547	1.14E+08	2163260	5342040	55352361	4086598	70877400	6	2.82E+08	1.72E+08	2.86E+08	35805343	236179322	2.34E+08	99628110	82752804
TNF	1	7	82694052	33689344	2.13E+08	4641560	22262760	54044387	7079488	99126060	7	2.42E+08	1.34E+08	3.02E+08	20463458	234120576	2.2E+08	1.41E+08	83738124
TNF	1.5	8	1.68E+08	32468090	2.1E+08	1.32E+08	67196175	48506495	7718936	1.14E+08	8	2.01E+08	1.48E+08	2.98E+08	2.7E+08	301739173	2.44E+08	99846102	96432192
TNF	2	9	1.74E+08	78479990	2.12E+08	1.27E+08	74485035	74544757	8335872	1.44E+08	9	2.8E+08	1.97E+08	2.54E+08	2.57E+08	253933437	2.84E+08	1.1E+08	84206358

			Fold of NS							
			BIRC3							
Stim	Concentration (ng/mL)	Lane	N = 1	N = 2	N = 3	N = 4	N = 5	N = 6	N = 7	N = 8
NS	-	1	1	1	1	1	1	1	1	1
TNF	-2.5	2	3.107289	0.519643	3.95229	3.2466	0.535965	3.928452	0.93627	1.354977
TNF	-2	3	5.454865	0.796179	5.717333	4.430309	0.50249	3.270404	2.401575	3.736364
TNF	-1.5	4	9.668954	6.665434	15.00767	10.32183	0.622945	10.19534	3.61063	8.689811
TNF	-1	5	34.62391	16.19908	23.3762	11.5748	1.197679	7.067443	4.563681	15.48142
TNF	-0.5	6	37.61596	33.51582	27.75782	2.995432	1.804675	13.13516	7.598522	18.69954
TNF	0	7	20.81851	28.44327	49.09996	11.24562	7.587056	13.63047	9.32641	25.84465
TNF	0.5	8	50.88414	24.84244	49.03746	24.19764	17.76833	11.04461	14.32107	25.82146
TNF	1	9	37.83696	45.16549	58.0857	24.47825	23.40362	14.57239	13.98894	37.26351

Supplemental Fig. S4 - A549 cells: protein

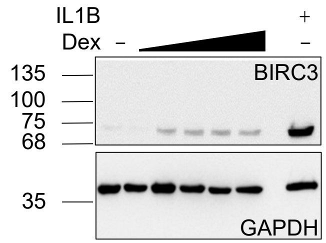

Chemiluminescence

		Adj. Total Band Vol.						Adj. Total Band Vol.				
		BIRC3						GAPDH				
Treatment	Concentration log (M)	N = 1	N = 2	N = 3	N = 4	N = 5	Lane	N = 1	N = 2	N = 3	N = 1	N = 5
NS	-	8293758	4165095	149656	1123977	817750	1	19529691	6595344	4643093	10700200	18124572
Dex	-10	4427790	4047700	89492	1021680	557700	2	16974828	5375772	7673400	7706560	17264359
Dex	-9	7332005	4395300	776412	1680096	1589700	3	23327934	3526812	6263768	5220680	14812092
Dex	-8	24248557	8174300	955916	2515113	2621900	4	13859202	4806360	5728345	5615760	17605523
Dex	-7	20750981	9643600	1005004	2378287	2725750	5	12371769	5722956	7788207	4021920	13856256
Dex	-6	29594996	10469190	938912	3055322	3657360	6	14746197	5663822	8811131	4966335	10983629
IL1B	1 ng/mL	1.4E+08	12463808	15596240	14403080	12924314	7	20716077	3873844	7900270	2873550	7520214

			Normalized Values				
			BIRC3				
Treatment	Concentration log (M)	Lane	N = 1	N = 2	N = 3	N = 4	N = 5
NS	-	1	1	1	1	1	1
Dex	-10	2	0.614222	1.192285	0.361834	1.262086	0.715974234
Dex	-9	3	0.7401	1.973416	3.845651	3.063667	2.378734549
Dex	-8	4	4.119948	2.693065	5.177306	4.263669	3.300763434
Dex	-7	5	3.949579	2.66828	4.003534	5.629441	4.360008433
Dex	-6	6	4.725875	2.926955	3.306028	5.856733	7.38021583
IL1B	1 ng/mL	9	15.858	5.09473	61.2479	47.71678	38.09118718

Supplemental Fig. S4 - A549 cells: protein

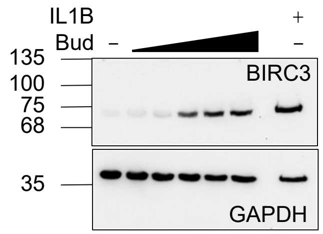

Chemiluminescence

		Adj. Total Band Vol.								Adj. Total Band Vol.						
		BIRC3								GAPDH						
Treatment	Concentration log (M)	N = 1	N = 2	N = 3	N = 4	N = 5	N = 6	N = 7	Lane	N = 1	N = 2	N = 3	N = 4	N = 5	N = 6	N = 7
NS	-	44960146	5427200	1434480	156085	4111030	4017780	5060220	1	1.23E+08	13014550	8095920	13993005	2.78E+08	1.69E+08	177284360
Bud	-10	11721240	9035440	2312454	609818	4410660	8137152	11735508	2	71432460	7542668	6945318	25676235	2.21E+08	1.84E+08	214796352
Bud	-9	63957842	25193868	2521176	524170	5988364	8953560	13154532	3	2.22E+08	11443215	10907066	22984272	2.01E+08	1.92E+08	176138880
Bud	-8	1.07E+08	29189432	2742090	608599	7976076	10126152	15515424	4	1.63E+08	14744198	8706399	9510039	2E+08	1.72E+08	126579456
Bud	-7	1.36E+08	18266132	3985541	2424379	12096720	10953984	17580924	5	1.95E+08	9427012	8634948	23089430	2.03E+08	1.56E+08	191745600
Bud	-6	1.61E+08	36079644	4484646	2573998	14503980	11805072	24383100	6	2.49E+08	13967408	11027232	23712104	2.52E+08	1.86E+08	174772416
IL1B	1 ng/mL	1.81E+08	1.47E+08	14699135	10761491	2.63E+08	97000980	95694360	9	2E+08	18385278	8092810	18885204	2.24E+08	1.73E+08	196085568

			Normalized Values						
			BIRC3						
Treatment	Concentration log (M)	Lane	N = 1	N = 2	N = 3	N = 4	N = 5	N = 6	N = 7
NS	-	1	1	1	1	1	1	1	1
Bud	-10	2	0.450711	2.872616	1.87911205	2.129211	1.348748	1.869268	1.91415
Bud	-9	3	0.790579	5.279589	1.304568637	2.044519	2.02062	1.963226	2.616503
Bud	-8	4	1.804931	4.747422	1.777521446	5.737184	2.697565	2.481771	4.29439
Bud	-7	5	1.908017	4.646499	2.60494924	9.413196	4.031578	2.959906	3.212309
Bud	-6	6	1.779774	6.194407	2.29526781	9.731683	3.891827	2.670519	4.887841
IL1B	1 ng/mL	9	2.483848	19.23817	10.2509507	51.08585	79.37483	23.69306	17.09786

Supplemental Fig. S5A – BEAS-2B cells

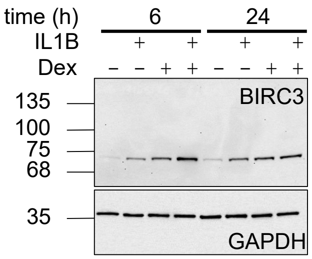

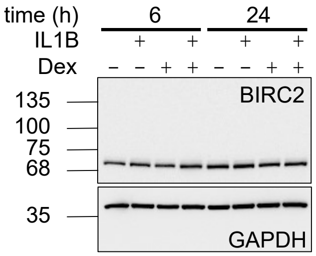

Chemiluminescence

			Adj. Total Band Vol.					Adj. Total Band Vol.								Normalized Values			
			BIRC3					GAPDH								BIRC3			
Time (h)	Stim	Lane	N = 1	N = 2	N = 3	N = 4	Lane	N = 1	N = 2	N = 3	N = 4		Time (h)	Stim	Lane	N = 1	N = 2	N = 3	N = 4
6	NS	1	7343752	2706626	2488044	433206	1	1.49E+08	1.01E+08	1.4E+08	3.11E+08		6	NS	1	0.049255	0.026689	0.017816	0.001395
	IL1B	2	30812091	20005518	20143464	18594894	2	1.35E+08	1.23E+08	1.84E+08	3.52E+08			IL1B	2	0.227399	0.162323	0.109696	0.052767
	Dex	3	71489758	42382730	33748494	36213906	3	1.39E+08	1.26E+08	1.82E+08	3.04E+08			Dex	3	0.515829	0.335066	0.185695	0.119073
	IL1B + Dex	4	1.42E+08	94331523	1.15E+08	1.42E+08	4	1.29E+08	1.34E+08	2.52E+08	3.23E+08			IL1B + Dex	4	1.101751	0.704752	0.456031	0.438492
24	NS	5	14219723	7204002	6299814	3639816	5	1.58E+08	1.55E+08	2.13E+08	4.15E+08		24	NS	5	0.090219	0.046494	0.029604	0.008762
	IL1B	6	70045686	36894390	26121510	37898514	6	1.61E+08	1.49E+08	1.7E+08	4.11E+08			IL1B	6	0.434528	0.247215	0.153304	0.092163
	Dex	7	1.12E+08	52890604	54899574	97373196	7	1.1E+08	1.33E+08	1.48E+08	3.87E+08			Dex	7	1.015869	0.397803	0.370212	0.251412
	IL1B + Dex	8	1.62E+08	69694508	80480130	1.01E+08	8	98006841	1.18E+08	1.48E+08	3.04E+08			IL1B + Dex	8	1.648477	0.589081	0.544199	0.331388

			Adj. Total Band Vol.					Adj. Total Band Vol.								Normalized Values			
			BIRC2					GAPDH								BIRC2			
Time (h)	Stim	Lane	N = 1	N = 2	N = 3	N = 4	Lane	N = 1	N = 2	N = 3	N = 4		Time (h)	Stim	Lane	N = 1	N = 2	N = 3	N = 4
6	NS	1	55824780	86561046	1.08E+08	1.68E+08	1	1.47E+08	3.1E+08	2.63E+08	1.65E+08		6	NS	1	0.379745	0.279219	0.411887	1.016569
	IL1B	2	69283291	85049895	1.38E+08	1.57E+08	2	1.92E+08	2.45E+08	2.6E+08	1.65E+08			IL1B	2	0.360814	0.346661	0.529504	0.953315
	Dex	3	58319569	98636048	1.17E+08	1.47E+08	3	1.77E+08	2.22E+08	2.36E+08	2.1E+08			Dex	3	0.329376	0.444556	0.493314	0.703772
	IL1B + Dex	4	50212668	92549415	1.69E+08	1.37E+08	4	1.36E+08	1.74E+08	2.5E+08	2.08E+08			IL1B + Dex	4	0.368603	0.531746	0.676042	0.659537
24	NS	5	92649260	1.49E+08	1.95E+08	1.3E+08	5	1.56E+08	2.15E+08	2.43E+08	1.67E+08		24	NS	5	0.593525	0.694946	0.803283	0.782121
	IL1B	6	1.29E+08	1.79E+08	2.17E+08	2.34E+08	6	2.6E+08	2.45E+08	2.84E+08	2.96E+08			IL1B	6	0.496208	0.732857	0.76255	0.791094
	Dex	7	1.08E+08	1.3E+08	1.7E+08	1.18E+08	7	1.79E+08	2.3E+08	2.98E+08	1.61E+08			Dex	7	0.600562	0.563794	0.569818	0.729689
	IL1B + Dex	8	1.18E+08	1.31E+08	1.94E+08	1.82E+08	8	1.82E+08	2.44E+08	3.3E+08	2.44E+08			IL1B + Dex	8	0.648073	0.537316	0.589305	0.746132

Supplemental Fig. S5A – CALU3 cells

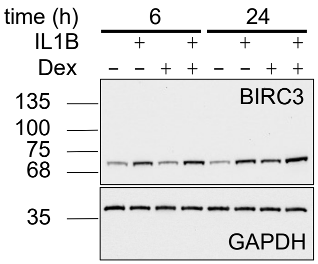

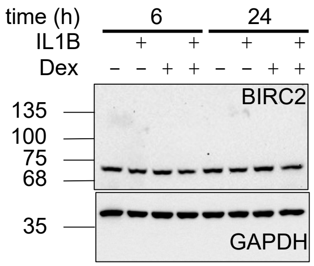

Chemiluminescence

			Adj. Total Band Vol.						Adj. Total Band Vol.				
			BIRC3						GAPDH				
Time (h)	Stim	Lane	N = 1	N = 2	N = 3	N = 4	N = 5	Lane	N = 1	N = 2	N = 3	N = 4	N = 5
6	NS	1	12732150	60783996	38788542	53799429	22546577	1	136284400	255256324	163069695	184870139	184870139
	IL1B	2	60440785	1.35E+08	92097018	1.16E+08	84456104	2	105903620	276346232	132659415	171636422	171636422
	Dex	3	12905580	56183556	38191608	68218373	44803510	3	99916132	314429492	130287405	181544343	181544343
	IL1B + Dex	4	91944355	1.72E+08	1.17E+08	2.03E+08	1.41E+08	4	89371724	234331564	128709210	150804166	150804166
24	NS	5	1013200	42672084	29365596	42368955	27811490	5	160547100	204923904	144482235	169984413	169984413
	IL1B	6	51683220	1.75E+08	1.15E+08	1.01E+08	1.14E+08	6	56615180	225671708	128668410	136985099	136985099
	Dex	7	23012805	1.03E+08	83827458	67962841	56600378	7	76178708	211002564	127835325	138338865	138338865
	IL1B + Dex	8	1.29E+08	3.32E+08	2.37E+08	2.62E+08	1.24E+08	8	65118964	247120674	127094295	146444644	146444644

			Normalized Values				
			BIRC3				
Time (h)	Stim	Lane	N = 1	N = 2	N = 3	N = 4	N = 5
6	NS	1	0.093423	0.238129	0.237865	0.291012	0.121959
	IL1B	2	0.570715	0.489808	0.694237	0.678508	0.492064
	Dex	3	0.129164	0.178684	0.293134	0.375767	0.246791
	IL1B + Dex	4	1.028786	0.734699	0.90799	1.347234	0.936677
24	NS	5	0.006311	0.208234	0.203247	0.249252	0.163612
	IL1B	6	0.912886	0.774058	0.892033	0.73382	0.828803
	Dex	7	0.30209	0.48808	0.655746	0.491278	0.409143
	IL1B + Dex	8	1.985447	1.344416	1.865309	1.791446	0.847898

			Adj. Total Band Vol.					Adj. Total Band Vol.			
			BIRC2					GAPDH			
Time (h)	Stim	Lane	N = 1	N = 2	N = 3	N = 4	Lane	N = 1	N = 2	N = 3	N = 4
6	NS	1	68378660	47573367	13045120	1.62E+08	1	1.77E+08	2.6E+08	3.19E+08	2.11E+08
	IL1B	2	55181000	45297382	13647620	50714820	2	1.48E+08	2.38E+08	2.46E+08	2.54E+08
	Dex	3	69949880	55046617	14896000	68730696	3	1.74E+08	2.22E+08	3.17E+08	2.52E+08
	IL1B + Dex	4	46379340	50010631	10197460	67812480	4	1.6E+08	2.26E+08	2.84E+08	2.02E+08
24	NS	5	71401400	24488453	17561880	1.02E+08	5	1.48E+08	2.27E+08	2.49E+08	1.69E+08
	IL1B	6	59560200	4993742	5717320	40141737	6	1.39E+08	1.97E+08	2.26E+08	1.53E+08
	Dex	7	82016060	13926379	12879720	93005601	7	1.37E+08	2.37E+08	2.23E+08	1.62E+08
	IL1B + Dex	8	54288920	25448788	6768300	43544543	8	1.56E+08	2.71E+08	2.54E+08	1.36E+08

			Adj. Total Band Vol.			
			Normalized Values			
Time (h)	Stim	Lane	N = 1	N = 2	N = 3	N = 4
6	NS	1	0.386687	0.183167	0.040877	0.765682
	IL1B	2	0.371621	0.189986	0.055502	0.199483
	Dex	3	0.402416	0.248474	0.047043	0.273067
	IL1B + Dex	4	0.290454	0.221069	0.035867	0.335059
24	NS	5	0.48226	0.107686	0.070533	0.599164
	IL1B	6	0.429054	0.025312	0.025273	0.262912
	Dex	7	0.598095	0.058831	0.057844	0.57388
	IL1B + Dex	8	0.347728	0.094059	0.02664	0.31945

Supplemental Fig. S5B - BEAS-2B: Protein

	6h				24h	
	BIRC3/GAPDH				BIRC3/GAPDH	
B2B	Sum (ΔIL1B + ΔDex)	Comb Δ(IL1B +Dex)			Sum (ΔIL1B + ΔDex)	Comb Δ(IL1B +Dex)
N = 1	0.7432284	1.101751		N = 1	1.450397	1.648477
N = 2	0.4973885	0.704752		N = 2	0.645017	0.589081
N = 3	0.2953916	0.456031		N = 3	0.523516	0.544199
N = 4	0.17184088	0.438492		N = 4	0.343575	0.331388

Supplemental Fig. S5B - Calu-3: Protein

	6h				24h	
	BIRC3/GAPDH				BIRC3/GAPDH	
CALU3	Sum (ΔIL1B + ΔDex)	Comb Δ(IL1B +Dex)			Sum (ΔIL1B + ΔDex)	Comb Δ(IL1B +Dex)
N = 1	0.699879	1.028786		N = 1	1.214976	1.985447
N = 2	0.668492	0.734699		N = 2	1.262139	1.344416
N = 3	0.98737	0.90799		N = 3	1.547779	1.865309
N = 4	1.054275	1.347234		N = 4	1.225098	1.791446
N = 5	0.738855	0.936677		N = 5	1.237946	0.847898

Supplemental Fig. S6 - A549 cells: protein

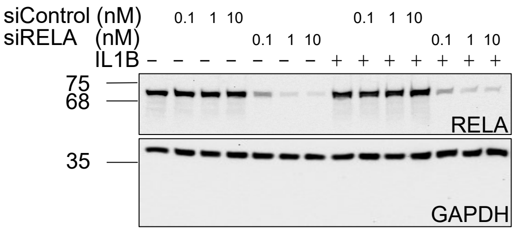

Chemiluminescence

				Adj. Total Band Vol.							Adj. Total Band Vol.					
				RELA							GAPDH					
Treatment	siRNA	Concentration (nM)	Lane	N = 1	N = 2	N = 3	N = 4	N = 5	N = 6	Lane	N = 1	N = 2	N = 3	N = 4	N = 5	N = 6
NS	-	-	1	82503063	1.72E+08	1.29E+08	1.7E+08	11565813	14505798	1	1.31E+08	1.12E+08	1.85E+08	81876306	4151845	74481666
NS	CTL	0.1	2	1.23E+08	4310768	1.06E+08	1.75E+08	36313263	16372779	2	2.4E+08	41038119	1.46E+08	1.04E+08	42309650	83267682
NS	CTL	1	3	1.3E+08	2829288	1.15E+08	1.45E+08	9173682	15724884	3	2.19E+08	37212584	1.56E+08	1.34E+08	25504125	90364691
NS	CTL	10	4	1.35E+08	1.8E+08	1.17E+08	1.3E+08	42037443	17013060	4	2.12E+08	1.07E+08	1.19E+08	1.36E+08	39096780	95606616
NS	RELA	0.1	5	26118822	60144238	22088970	28696390	11718546	9791886	5	2.18E+08	1.39E+08	1.38E+08	1.21E+08	58391135	1.01E+08
NS	RELA	1	6	10212014	26975564	5761935	6095685	433062	6525621	6	2.43E+08	1.19E+08	1.07E+08	1.56E+08	13849220	99551998
NS	RELA	10	7	8267666	24791074	5050215	4685495	345450	7845369	7	1.83E+08	1.48E+08	1.37E+08	1.46E+08	22123240	1.05E+08
IL1B	-	-	8	1.18E+08	1.99E+08	1.03E+08	1.13E+08	20697747	19679793	8	2.05E+08	1.29E+08	1.09E+08	1.84E+08	13498125	1E+08
IL1B	CTL	0.1	9	1.3E+08	1.79E+08	98616285	1.07E+08	44916585	19314885	9	2.95E+08	1.65E+08	1.48E+08	1.41E+08	73498225	1.12E+08
IL1B	CTL	1	10	1.38E+08	1.68E+08	1.05E+08	1.13E+08	44283897	19038525	10	2.16E+08	1.4E+08	1.16E+08	1.92E+08	54499305	1.12E+08
IL1B	CTL	10	11	1.32E+08	2.03E+08	1.08E+08	1.52E+08	46907994	19237053	11	2.33E+08	1.76E+08	1.29E+08	1.67E+08	59081940	1.18E+08
IL1B	RELA	0.1	12	25367848	54695102	15708870	21674890	12166161	10259724	12	2.16E+08	1.97E+08	1.51E+08	1.78E+08	58076380	1.03E+08
IL1B	RELA	1	13	7915800	19804309	4462020	9286980	7549292	6072165	13	2.86E+08	1.89E+08	1.26E+08	1.95E+08	43593050	1.21E+08
IL1B	RELA	10	14	4213020	22758890	2956500	8003580	5610696	5460930	14	1.82E+08	2.49E+08	1.62E+08	1.72E+08	77960800	1.79E+08

				Normalized Values					
				RELA					
Treatment	siRNA	Concentration (nM)	Lane	N = 1	N = 2	N = 3	N = 4	N = 5	N = 6
NS	-	-	1	0.629571	1.530466	0.698833	2.077186	2.785704	0.194757
NS	CTL	0.1	2	0.512219	0.105043	0.729169	1.692497	0.858274	0.196628
NS	CTL	1	3	0.590525	0.07603	0.737307	1.07917	0.359694	0.174016
NS	CTL	10	4	0.637291	1.685309	0.978429	0.95532	1.075215	0.177949
NS	RELA	0.1	5	0.11955	0.432201	0.159594	0.23757	0.20069	0.096745
NS	RELA	1	6	0.042058	0.22623	0.05367	0.039101	0.03127	0.06555
NS	RELA	10	7	0.045096	0.167862	0.036809	0.032156	0.015615	0.075021
IL1B	-	-	8	0.576567	1.54579	0.944053	0.612891	1.533379	0.196133
IL1B	CTL	0.1	9	0.441767	1.083942	0.666844	0.756616	0.611125	0.17224
IL1B	CTL	1	10	0.640238	1.201018	0.904086	0.587723	0.812559	0.169908
IL1B	CTL	10	11	0.566076	1.149818	0.841408	0.908321	0.793948	0.162876
IL1B	RELA	0.1	12	0.117349	0.278115	0.103729	0.121906	0.209486	0.099494
IL1B	RELA	1	13	0.027656	0.104649	0.035327	0.047574	0.173177	0.050158
IL1B	RELA	10	14	0.023117	0.09137	0.018208	0.046533	0.071968	0.03044

Supplemental Fig. S6 - A549 cells: protein

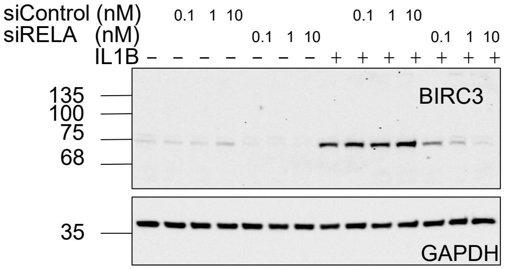

Chemiluminescence

Treatment	siRNA	Concentration (nM)	Lane	Adj. Total Band Vol. BIRC3 N = 1	N = 2	N = 3	N = 4	N = 5	N = 6
NS	-	-	1	3904794	3571355	3716232	273182	95823	8813420
NS	CTL	0.1	2	7125890	1334395	3688620	358818	916370	10827040
NS	CTL	1	3	10453200	598455	2835924	514132	32965	11667880
NS	CTL	10	4	14965650	8509810	7170540	3865628	1499100	17525060
NS	RELA	0.1	5	2777472	2495190	1353612	2476808	532714	11021220
NS	RELA	1	6	1309536	1008430	605748	1948140	68250	11080160
NS	RELA	10	7	1089360	461590	690768	1078982	60333	9643008
IL1B	-	-	8	85551164	58514825	55564548	57861180	7102823	1.04E+08
IL1B	CTL	0.1	9	1.2E+08	49979440	79996800	61518248	32937996	1.14E+08
IL1B	CTL	1	10	1.28E+08	65189280	68435016	63500832	33066579	1.03E+08
IL1B	CTL	10	11	1.33E+08	65866475	1.3E+08	76009534	36058113	99741460
IL1B	RELA	0.1	12	61685856	37073520	25640940	45042166	22218651	1.2E+08
IL1B	RELA	1	13	10300370	7460770	8046636	11951752	10440703	90392820
IL1B	RELA	10	14	3673560	6808530	2620176	3068676	9164155	67903080

Treatment	siRNA	Concentration (nM)	Lane	Adj. Total Band Vol. GAPDH N = 1	N = 2	N = 3	N = 4	N = 5	N = 6
NS	-	-	1	1.14E+08	1.38E+08	1.37E+08	81876306	3148860	94777332
NS	CTL	0.1	2	2.44E+08	60982614	1.21E+08	1.04E+08	42443072	77601699
NS	CTL	1	3	2.58E+08	57262548	1.1E+08	1.34E+08	16959712	73964820
NS	CTL	10	4	2.23E+08	1.14E+08	95975740	1.36E+08	37107280	72030630
NS	RELA	0.1	5	2.33E+08	1.21E+08	1.13E+08	1.21E+08	48646752	76278762
NS	RELA	1	6	2.62E+08	1.01E+08	79940140	1.56E+08	20564768	63419125
NS	RELA	10	7	2.05E+08	1.22E+08	94209500	1.46E+08	23088688	64987659
IL1B	-	-	8	2.35E+08	1.08E+08	70845600	1.84E+08	12634800	55527420
IL1B	CTL	0.1	9	2.53E+08	1.17E+08	1.11E+08	1.41E+08	53119024	68324758
IL1B	CTL	1	10	2.6E+08	1.03E+08	78558900	1.92E+08	33618032	88307310
IL1B	CTL	10	11	2.3E+08	1.13E+08	98153473	1.67E+08	47462352	1.05E+08
IL1B	RELA	0.1	12	2.31E+08	1.18E+08	1.09E+08	1.78E+08	45543568	95234908
IL1B	RELA	1	13	2.6E+08	99680735	1.12E+08	1.95E+08	38469088	1.24E+08
IL1B	RELA	10	14	2.01E+08	1.53E+08	1.38E+08	1.72E+08	56169120	1.52E+08

				Normalized Values					
				BIRC3					
Treatment	siRNA	Concentration (nM)	Lane	N = 1	N = 2	N = 3	N = 4	N = 5	N = 6
NS	-	-	1	0.034139	0.02579	0.027049	0.003337	0.030431	0.092991
NS	CTL	0.1	2	0.029155	0.021882	0.030437	0.003466	0.021591	0.139521
NS	CTL	1	3	0.04045	0.010451	0.025765	0.003826	0.001944	0.157749
NS	CTL	10	4	0.066991	0.074947	0.074712	0.028476	0.040399	0.2433
NS	RELA	0.1	5	0.011933	0.020545	0.011996	0.020505	0.010951	0.144486
NS	RELA	1	6	0.004991	0.009994	0.007578	0.012497	0.003319	0.174713
NS	RELA	10	7	0.005321	0.003796	0.007332	0.007405	0.002613	0.148382
IL1B	-	-	8	0.36343	0.542089	0.784305	0.313713	0.562163	1.880093
IL1B	CTL	0.1	9	0.474566	0.42699	0.721394	0.436343	0.620079	1.672775
IL1B	CTL	1	10	0.49335	0.63414	0.87113	0.329983	0.983597	1.169103
IL1B	CTL	10	11	0.580199	0.584132	1.326689	0.454889	0.75972	0.953807
IL1B	RELA	0.1	12	0.266471	0.312999	0.235151	0.25333	0.487855	1.256248
IL1B	RELA	1	13	0.039688	0.074847	0.071573	0.061225	0.271405	0.727732
IL1B	RELA	10	14	0.018273	0.044469	0.018929	0.017841	0.163153	0.44596

Supplemental Fig. S6 - A549 cells: protein

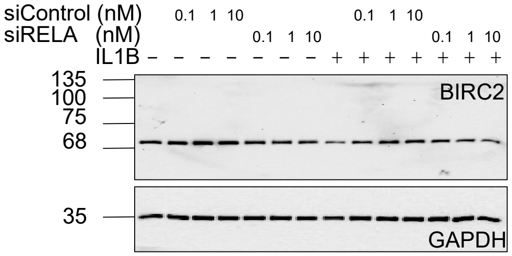

Chemiluminescence

				Adj. Total Band Vol.							Adj. Total Band Vol.					
				BIRC2							GAPDH					
Treatment	siRNA	Concentration (nM)	Lane	N = 1	N = 2	N = 3	N = 4	N = 5	N = 6	Lane	N = 1	N = 2	N = 3	N = 4	N = 5	N = 6
NS	-	-	1	43870288	16039748	59031792	25378984	196896	7866658	1	1.43E+08	1.48E+08	1.85E+08	2.2E+08	3265600	28954470
NS	CTL	0.1	2	85487556	17272	69893856	23259344	5126112	8369196	2	1.1E+08	59590844	1.46E+08	2.18E+08	32811856	34189985
NS	CTL	1	3	1.15E+08	20672	43228656	31810560	174432	7654368	3	2.09E+08	77696232	1.56E+08	2.21E+08	8989120	34130825
NS	CTL	10	4	1.13E+08	4673504	82502496	40514992	5940096	9331388	4	2.08E+08	1.31E+08	1.19E+08	2.32E+08	34529376	43015555
NS	RELA	0.1	5	86784390	7048608	44704800	27191280	1752768	8367492	5	2.21E+08	1.98E+08	1.38E+08	2.37E+08	13756400	43075585
NS	RELA	1	6	63036358	3722184	48590208	26979240	73248	8606904	6	2.59E+08	1.46E+08	1.07E+08	2.54E+08	11860912	48105200
NS	RELA	10	7	53167730	8406024	38441952	20150488	151872	4396462	7	2.57E+08	1.98E+08	1.37E+08	2.55E+08	4562544	33009375
IL1B	-	-	8	1.32E+08	5336640	67666320	57600000	1152672	17946528	8	1.91E+08	1.56E+08	1.09E+08	2.78E+08	6991680	29710736
IL1B	CTL	0.1	9	1.67E+08	5615440	90893664	36106992	4848192	19452012	9	1.65E+08	1.84E+08	1.48E+08	2.76E+08	24671696	47528390
IL1B	CTL	1	10	2.05E+08	15892960	81218880	42506496	5486688	14762604	10	2.21E+08	1.62E+08	1.16E+08	2.41E+08	20152496	24685670
IL1B	CTL	10	11	1.77E+08	17985184	1.11E+08	57819736	7740384	13717484	11	2.51E+08	1.51E+08	1.29E+08	2.92E+08	46623920	23686185
IL1B	RELA	0.1	12	1.13E+08	18029656	31711680	31312608	4126656	11048452	12	2.14E+08	2.1E+08	1.51E+08	2.66E+08	18040624	31533295
IL1B	RELA	1	13	94736488	14440616	75191760	26727224	7593504	11370508	13	2.1E+08	1.49E+08	1.26E+08	2.71E+08	27405392	12557000
IL1B	RELA	10	14	84092778	28804800	48118464	22318008	6364992	9244200	14	1.76E+08	2.41E+08	1.62E+08	2.54E+08	16644768	18131235

				Normalized Values					
				BIRC2					
Treatment	siRNA	Concentration (nM)	Lane	N = 1	N = 2	N = 3	N = 4	N = 5	N = 6
NS	-	-	1	0.306125	0.108733	0.319076	0.11541	0.060294	0.271691
NS	CTL	0.1	2	0.778491	0.00029	0.478778	0.106482	0.156227	0.244785
NS	CTL	1	3	0.548628	0.000266	0.277297	0.144245	0.019405	0.224266
NS	CTL	10	4	0.546116	0.035725	0.692015	0.174613	0.17203	0.216931
NS	RELA	0.1	5	0.392044	0.035639	0.322994	0.114536	0.127415	0.194251
NS	RELA	1	6	0.24373	0.025417	0.452595	0.106057	0.006176	0.178918
NS	RELA	10	7	0.206605	0.042369	0.280191	0.078892	0.033287	0.133188
IL1B	-	-	8	0.69013	0.03416	0.620343	0.207169	0.164863	0.604042
IL1B	CTL	0.1	9	1.011999	0.030595	0.614624	0.130694	0.196508	0.409271
IL1B	CTL	1	10	0.923528	0.097947	0.697688	0.176297	0.272258	0.598023
IL1B	CTL	10	11	0.705628	0.119006	0.867091	0.197853	0.166017	0.579134
IL1B	RELA	0.1	12	0.526605	0.08573	0.209399	0.11761	0.228742	0.350374
IL1B	RELA	1	13	0.451643	0.097215	0.595311	0.098474	0.277081	0.905512
IL1B	RELA	10	14	0.477885	0.119679	0.296338	0.087929	0.382402	0.509849

Supplemental Fig. S6 - A549 cells: protein

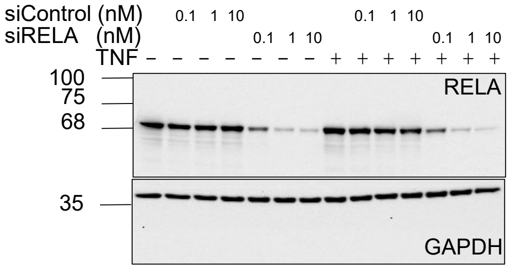

Chemiluminescence

				Adj. Total Band Vol.							Adj. Total Band Vol.					
				RELA							GAPDH					
Treatment	siRNA	Concentration (nM)	Lane	N = 1	N = 2	N = 3	N = 4	N = 5	N = 6	Lane	N = 1	N = 2	N = 3	N = 4	N = 5	N = 6
NS	-	-	1	30732625	1.43E+08	4161984	17060718	62108899	60795474	1	16098192	1.39E+08	1.33E+08	52167456	2.89E+08	2.56E+08
NS	CTL	0.1	2	55266300	1.29E+08	1.34E+08	18865518	57791223	59204265	2	23588738	1.29E+08	1.69E+08	55710204	3.2E+08	3E+08
NS	CTL	1	3	60725538	1.16E+08	1.12E+08	18295314	56996445	51849918	3	24488172	1.2E+08	1.6E+08	56906784	3.84E+08	2.79E+08
NS	CTL	10	4	60269022	1.31E+08	1.14E+08	18210009	60076419	57908124	4	25873457	1.29E+08	1.59E+08	61825896	4.06E+08	3.18E+08
NS	RELA	0.1	5	8604954	34472736	26198760	10740111	17774346	19525629	5	28009813	1.24E+08	1.55E+08	57036804	4.3E+08	4.13E+08
NS	RELA	1	6	2123334	9226656	5717040	8120895	6576123	6494562	6	26747704	1.31E+08	1.36E+08	54568800	3.97E+08	3.68E+08
NS	RELA	10	7	1891188	8737920	4207000	8000325	7123707	7054887	7	21359199	1.2E+08	1.34E+08	62089638	3.29E+08	3.47E+08
TNF	-	-	8	59748678	1.7E+08	1.38E+08	22488295	69707220	60590244	8	26252419	1.35E+08	1.33E+08	56035046	3.2E+08	3.22E+08
TNF	CTL	0.1	9	53273538	1.29E+08	1.29E+08	19306707	63408609	60577038	9	27470028	1.31E+08	1.44E+08	52547901	3.25E+08	3.38E+08
TNF	CTL	1	10	66673692	97674768	1.18E+08	19778916	66650496	64425657	10	30673672	1.36E+08	1.47E+08	55516692	3.09E+08	3.2E+08
TNF	CTL	10	11	67975038	74343168	92004920	19523001	74284215	58054413	11	26557867	1.31E+08	1.48E+08	56691228	4.32E+08	3.76E+08
TNF	RELA	0.1	12	8800164	35580816	27841800	10248444	6194451	5836866	12	25603452	1.35E+08	1.62E+08	54850884	2.19E+08	2.25E+08
TNF	RELA	1	13	2172744	8083296	8925420	6404784	10194009	9113721	13	24759311	1.31E+08	1.79E+08	60789696	3.26E+08	2.94E+08
TNF	RELA	10	14	969570	2682864	5143180	5654241	7729881	6215655	14	40959715	1.1E+08	2.54E+08	59387460	3.63E+08	2.66E+08

				Normalized Values					
				RELA					
Treatment	siRNA	Concentration (nM)	Lane	N = 1	N = 2	N = 3	N = 4	N = 5	N = 6
NS	-	-	1	1.909073	1.029244	0.031212	0.327038	0.21488	0.237565
NS	CTL	0.1	2	2.34291	1.001294	0.791384	0.338637	0.18038	0.197316
NS	CTL	1	3	2.479791	0.966926	0.697101	0.321496	0.148512	0.185753
NS	CTL	10	4	2.329376	1.01636	0.71817	0.294537	0.147881	0.18206
NS	RELA	0.1	5	0.307212	0.277587	0.168584	0.188301	0.041312	0.047276
NS	RELA	1	6	0.079384	0.070422	0.041957	0.148819	0.016578	0.017662
NS	RELA	10	7	0.088542	0.072584	0.031498	0.128851	0.02165	0.020328
IL1B	-	-	8	2.27593	1.260988	1.03787	0.401326	0.217837	0.188174
IL1B	CTL	0.1	9	1.939333	0.990152	0.894147	0.367412	0.194917	0.179476
IL1B	CTL	1	10	2.173646	0.718455	0.806175	0.35627	0.215859	0.201426
IL1B	CTL	10	11	2.559507	0.569258	0.620037	0.344374	0.171756	0.154224
IL1B	RELA	0.1	12	0.34371	0.262659	0.17196	0.186842	0.028291	0.02597
IL1B	RELA	1	13	0.087755	0.061549	0.049833	0.10536	0.031257	0.031051
IL1B	RELA	10	14	0.023671	0.024494	0.020228	0.095209	0.021282	0.023339

Supplemental Fig. S6 - A549 cells: protein

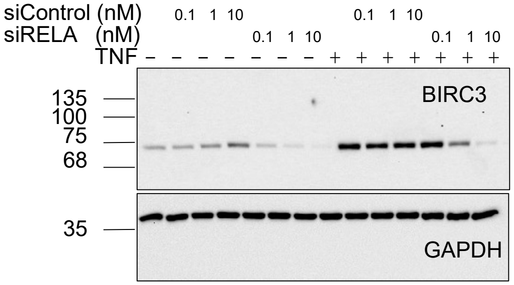

Chemiluminescence

				Adj. Total Band Vol.						Adj. Total Band Vol.				
				BIRC3						GAPDH				
Treatment	siRNA	Concentration (nM)	Lane	N = 1	N = 2	N = 3	N = 4	N = 5	Lane	N = 1	N = 2	N = 3	N = 4	N = 5
NS	-	-	1	426320	9233400	129788	8340640	56136402	1	19337157	49008584	8561408	71989020	1.49E+08
NS	CTL	0.1	2	4388520	8635836	50521754	8372280	52138269	2	28586095	52635400	23069425	81214065	2.05E+08
NS	CTL	1	3	7433880	11949564	88827248	8952020	40450140	3	27944260	50147620	23951555	85339315	2.3E+08
NS	CTL	10	4	10126680	20604012	98476716	12404980	51130611	4	27607660	59697704	22835296	1.12E+08	3.24E+08
NS	RELA	0.1	5	2313720	5011908	23123138	8528100	29584104	5	29232520	59650960	26056750	1.1E+08	3.18E+08
NS	RELA	1	6	1709160	2373756	3034114	7898520	23563848	6	26970755	65323350	27384620	1.53E+08	3.12E+08
NS	RELA	10	7	984000	1646040	1046540	8106876	23047200	7	25679010	57744070	22778555	1.48E+08	2.87E+08
TNF	-	-	8	49703400	65307660	9928356	59492580	2.28E+08	8	30622610	60241880	23426935	94538840	2.71E+08
TNF	CTL	0.1	9	46394400	51352092	10849652	62273680	1.22E+08	9	33112090	57899365	24566870	88041390	2.56E+08
TNF	CTL	1	10	72334920	57009876	12419036	54278980	3.14E+08	10	37419635	58042165	23166920	75644325	3.89E+08
TNF	CTL	10	11	67324440	69693888	33001226	63928620	2.81E+08	11	26549665	61956415	27692915	90891075	1.99E+08
TNF	RELA	0.1	12	9873600	21035520	7672402	46661720	27401220	12	30057870	62226630	22057160	95194965	2E+08
TNF	RELA	1	13	911820	2676696	3437252	32885020	96636144	13	26396835	66699670	14475075	1.14E+08	3.15E+08
TNF	RELA	10	14	350546	339504	1119244	20759960	70256340	14	40959715	71501416	8755317	1.16E+08	2.67E+08

				Adj. Total Band Vol.				
				BIRC3				
Treatment	siRNA	Concentration (nM)	Lane	N = 1	N = 2	N = 3	N = 4	N = 5
NS	-	-	1	0.022046674	0.188404	0.01516	0.11586	0.376901
NS	CTL	0.1	2	0.153519395	0.164069	2.189988	0.103089	0.254352
NS	CTL	1	3	0.266025295	0.238288	3.708621	0.104899	0.176022
NS	CTL	10	4	0.366806893	0.345139	4.312478	0.110321	0.158003
NS	RELA	0.1	5	0.079148838	0.084021	0.887415	0.07749	0.093062
NS	RELA	1	6	0.063370862	0.036339	0.110796	0.051583	0.075564
NS	RELA	10	7	0.038319234	0.028506	0.045944	0.054713	0.080311
IL1B	-	-	8	1.623094831	1.084091	0.423801	0.629292	0.843073
IL1B	CTL	0.1	9	1.401131732	0.88692	0.441638	0.707323	0.475704
IL1B	CTL	1	10	1.933073906	0.982215	0.536068	0.717555	0.805426
IL1B	CTL	10	11	2.535792448	1.124886	1.191685	0.703354	1.410064
IL1B	RELA	0.1	12	0.32848635	0.338047	0.347842	0.49017	0.136805
IL1B	RELA	1	13	0.034542778	0.040131	0.23746	0.288461	0.306652
IL1B	RELA	10	14	0.008558312	0.004748	0.127836	0.179183	0.262922

Supplemental Fig. S6 - A549 cells: protein

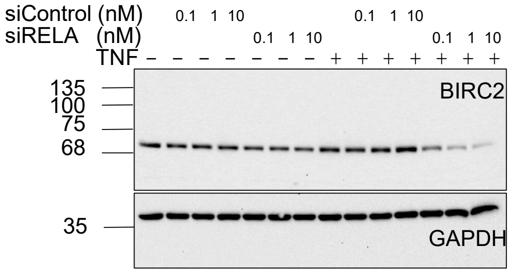

Chemiluminescence

				Adj. Total Band Vol.						Adj. Total Band Vol.				
				BIRC2						GAPDH				
Treatment	siRNA	Concentration (nM)	Lane	N = 1	N = 2	N = 3	N = 4	N = 5	Lane	N = 1	N = 2	N = 3	N = 4	N = 5
NS	-	-	1	16336086	44177375	1153960	27975704	2.13E+08	1	18611068	62847658	12742896	77440500	1.49E+08
NS	CTL	0.1	2	24831240	37349000	63381984	27994164	2.19E+08	2	18471775	59590256	29581785	85795875	2.05E+08
NS	CTL	1	3	26864880	38862375	80550760	24675198	1.98E+08	3	16064405	54387784	28686225	85551625	2.3E+08
NS	CTL	10	4	25854120	40569625	73230016	22358326	2.01E+08	4	13845395	64212456	28321920	90895125	3.24E+08
NS	RELA	0.1	5	21627480	32416125	14093816	9885188	1.87E+08	5	13977315	65678649	27948340	83733500	3.18E+08
NS	RELA	1	6	22732560	31866125	3957872	4108912	1.61E+08	6	16182385	54690592	29263460	92839750	3.12E+08
NS	RELA	10	7	14474520	28722750	2287112	7196844	1.66E+08	7	13164885	61980600	29223425	96488328	2.87E+08
TNF	-	-	8	26101800	47009875	19213128	56390756	1.12E+08	8	16243245	65644656	30437480	1.08E+08	2.71E+08
TNF	CTL	0.1	9	24715080	41933000	29773664	30796534	1E+08	9	19484040	60737864	33628465	79752625	2.56E+08
TNF	CTL	1	10	42687936	46950000	35505112	27714140	1.92E+08	10	22196900	60312472	29682340	79300125	3.89E+08
TNF	CTL	10	11	38439600	66428375	47789448	40354270	2.2E+08	11	19044335	69146528	20991515	83737750	1.99E+08
TNF	RELA	0.1	12	12430680	21863125	41342776	33654994	28579329	12	26752475	69113440	12447230	78295375	2E+08
TNF	RELA	1	13	6652958	9566750	38985080	33018550	1.06E+08	13	32708595	71358936	9789790	86478500	3.15E+08
TNF	RELA	10	14	5632534	5323250	30761432	38445882	1.02E+08	14	40959715	73849752	8460825	57533750	2.67E+08

				Adj. Total Band Vol.				
				BIRC2				
Treatment	siRNA	Concentration (nM)	Lane	N = 1	N = 2	N = 3	N = 4	N = 5
NS	-	-	1	0.877761878	0.702928	0.090557	0.361254	1.74E+08
NS	CTL	0.1	2	1.344280125	0.626764	2.142602	0.326288	2.15E+08
NS	CTL	1	3	1.672323376	0.714542	2.807994	0.288425	2.02E+08
NS	CTL	10	4	1.867344341	0.631803	2.58563	0.245979	1.72E+08
NS	RELA	0.1	5	1.547327223	0.493557	0.504281	0.118055	1.66E+08
NS	RELA	1	6	1.404771917	0.582662	0.13525	0.044258	1.71E+08
NS	RELA	10	7	1.099479411	0.463415	0.078263	0.074588	1.59E+08
IL1B	-	-	8	1.606932605	0.716126	0.631233	0.520594	1.33E+08
IL1B	CTL	0.1	9	1.268478201	0.690393	0.885371	0.386151	1.78E+08
IL1B	CTL	1	10	1.923148548	0.778446	1.19617	0.349484	2.63E+08
IL1B	CTL	10	11	2.018427002	0.96069	2.276608	0.481913	3.85E+08
IL1B	RELA	0.1	12	0.464655326	0.316337	3.321444	0.429847	1.15E+08
IL1B	RELA	1	13	0.20340091	0.134065	3.982218	0.381812	2.71E+08
IL1B	RELA	10	14	0.137513994	0.072082	3.635749	0.668232	3.18E+08

Supplemental Fig. S7A - A549: mRNA

		BIRC3				
Organon34517	Log [Bud]	N = 1	N = 2	N = 3	N = 4	N = 5
-	-9	1.088361	2.210685	1.720569	1.412871	1.948941
-	-8.5	1.815578	3.852348	4.53179	2.152442	3.405899
-	-8	2.945777	7.97044	3.278682	3.080133	4.429646
-	-7.5	2.734232	7.760392	3.930206	4.162534	4.344096
-	-7	1.995772	5.349508	6.1442	3.435236	3.90269
-	-6.5	2.049991	6.246037	6.238001	3.723643	2.997736
-	-6	2.089805	8.278636	7.117584	4.690848	3.495154
+	-9	0.802985	0.737865	0.777844	0.900866	0.757711
+	-8.5	1.1326	1.183532	0.824005	0.675765	0.954413
+	-8	1.279953	1.325656	0.986476	1.502296	0.873806
+	-7.5	1.537025	1.409835	1.11149	0.97635	0.807884
+	-7	1.68963	1.614207	0.959857	1.285077	1.489709
+	-6.5	4.845114	3.986602	4.080792	2.007863	2.41273
+	-6	4.704263	6.825679	6.837136	4.255223	4.600476

Supplemental Fig. S7B
# - A549 cells: protein

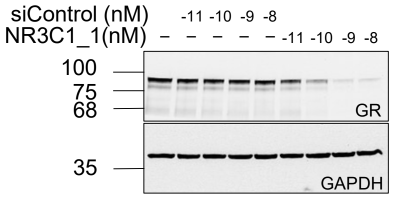

Chemiluminescence

				Adj. Total Band Vol.						Adj. Total Band Vol.				
				GR						GAPDH				
Treatment	siRNA	Concentration (nM)	Lane	N = 1	N = 2	N = 3	N = 4	N = 5	Lane	N = 1	N = 2	N = 3	N = 4	N = 5
NS	-	-	1	26562010	2.15E+08	2.29E+08	45592368	129198049	1	1.73E+08	2.25E+08	4.12E+08	16630725	206528548
NS	CTL	0.01	2	61078110	1.28E+08	1.76E+08	46993457	102984238	2	2.59E+08	1.62E+08	3.39E+08	27472235	196953706
NS	CTL	0.1	3	31763690	1.63E+08	1.52E+08	44394564	97728927	3	1.36E+08	1.76E+08	4.01E+08	27658075	185037469
NS	CTL	1	4	22448690	2.12E+08	1.53E+08	22391152	102561147	4	2.81E+08	2.52E+08	4.1E+08	11228370	238454350
NS	CTL	10	5	23360870	20584810	1.51E+08	43904490	59664912	5	1.7E+08	1.08E+08	3.8E+08	26617440	170920685
NS	NR3C1_1	0.01	6	7210040	45764276	98126947	19588485	42672437	6	1.37E+08	96485952	3.94E+08	16154165	160814079
NS	NR3C1_1	0.1	7	2154870	36921212	47660663	25696545	28108322	7	1.82E+08	1.86E+08	4.39E+08	24806420	208107346
NS	NR3C1_1	1	8	1617820	21615768	12280059	6509685	10505833	8	1.05E+08	1.38E+08	3.87E+08	9724170	159723769
NS	NR3C1_1	10	9	1394490	20736179	11417269	4740435	9572093	9	48317404	1.66E+08	4.45E+08	6501295	166325069

				Fold Values				
				GR				
Treatment	siRNA	Concentration (nM)	Lane	N = 1	N = 2	N = 3	N = 4	N = 5
NS	-	-	1	1	1	1	1	1
NS	CTL	0.01	2	1.529588	0.824547	0.93288	0.623968	0.835855
NS	CTL	0.1	3	1.520872	0.969058	0.680307	0.5855	0.844282
NS	CTL	1	4	0.519577	0.881486	0.67	0.727409	0.687546
NS	CTL	10	5	0.894466	0.199776	0.713347	0.601675	0.558018
NS	NR3C1_1	0.01	6	0.34277	0.495762	0.447327	0.442319	0.424177
NS	NR3C1_1	0.1	7	0.076735	0.207279	0.19498	0.377859	0.215909
NS	NR3C1_1	1	8	0.100334	0.164274	0.057003	0.244189	0.105144
NS	NR3C1_1	10	9	0.187558	0.130853	0.046089	0.265973	0.091997

Supplemental Fig. S7B
# - A549 cells: protein

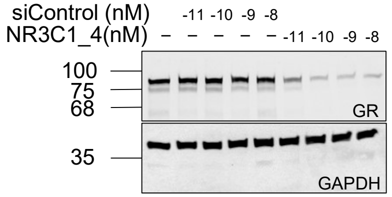

Chemiluminescence

				Adj. Total Band Vol.						Adj. Total Band Vol.				
				GR						GAPDH				
Treatment	siRNA	Concentration (nM)	Lane	N = 1	N = 2	N = 3	N = 4	N = 5	Lane	N = 1	N = 2	N = 3	N = 4	N = 5
NS	-	-	1	26562010	2.15E+08	2.29E+08	45592368	129198049	1	1.73E+08	2.25E+08	4.12E+08	16630725	206528548
NS	CTL	0.01	2	61078110	1.28E+08	1.76E+08	46993457	102984238	2	2.59E+08	1.62E+08	3.39E+08	27472235	196953706
NS	CTL	0.1	3	31763690	1.63E+08	1.52E+08	44394564	97728927	3	1.36E+08	1.76E+08	4.01E+08	27658075	185037469
NS	CTL	1	4	22448690	2.12E+08	1.53E+08	22391152	102561147	4	2.81E+08	2.52E+08	4.1E+08	11228370	238454350
NS	CTL	10	5	23360870	20584810	1.51E+08	43904490	59664912	5	1.7E+08	1.08E+08	3.8E+08	26617440	170920685
NS	NR3C1_1	0.01	6	7210040	45764276	98126947	19588485	42672437	6	1.37E+08	96485952	3.94E+08	16154165	160814079
NS	NR3C1_1	0.1	7	2154870	36921212	47660663	25696545	28108322	7	1.82E+08	1.86E+08	4.39E+08	24806420	208107346
NS	NR3C1_1	1	8	1617820	21615768	12280059	6509685	10505833	8	1.05E+08	1.38E+08	3.87E+08	9724170	159723769
NS	NR3C1_1	10	9	1394490	20736179	11417269	4740435	9572093	9	48317404	1.66E+08	4.45E+08	6501295	166325069

				Fold Values				
				GR				
Treatment	siRNA	Concentration (nM)	Lane	N = 1	N = 2	N = 3	N = 4	N = 5
NS	-	-	1	1	1	1	1	1
NS	CTL	0.01	2	1.529588	0.824547	0.93288	0.623968	0.835855
NS	CTL	0.1	3	1.520872	0.969058	0.680307	0.5855	0.844282
NS	CTL	1	4	0.519577	0.881486	0.67	0.727409	0.687546
NS	CTL	10	5	0.894466	0.199776	0.713347	0.601675	0.558018
NS	NR3C1_1	0.01	6	0.34277	0.495762	0.447327	0.442319	0.424177
NS	NR3C1_1	0.1	7	0.076735	0.207279	0.19498	0.377859	0.215909
NS	NR3C1_1	1	8	0.100334	0.164274	0.057003	0.244189	0.105144
NS	NR3C1_1	10	9	0.187558	0.130853	0.046089	0.265973	0.091997

Supplemental Fig. S7B
# - A549 cells: protein

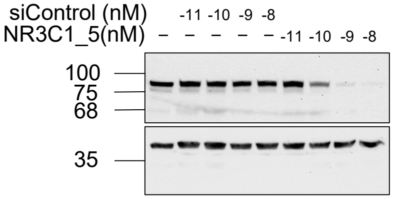

Chemiluminescence

				Adj. Total Band Vol.						Adj. Total Band Vol.				
				GR						GAPDH				
Treatment	siRNA	Concentration (nM)	Lane	N = 1	N = 2	N = 3	N = 4	N = 5	Lane	N = 1	N = 2	N = 3	N = 4	N = 5
NS	-	-	1	26562010	2.15E+08	2.29E+08	45592368	129198049	1	1.73E+08	2.25E+08	4.12E+08	16630725	206528548
NS	CTL	0.01	2	61078110	1.28E+08	1.76E+08	46993457	102984238	2	2.59E+08	1.62E+08	3.39E+08	27472235	196953706
NS	CTL	0.1	3	31763690	1.63E+08	1.52E+08	44394564	97728927	3	1.36E+08	1.76E+08	4.01E+08	27658075	185037469
NS	CTL	1	4	22448690	2.12E+08	1.53E+08	22391152	102561147	4	2.81E+08	2.52E+08	4.1E+08	11228370	238454350
NS	CTL	10	5	23360870	20584810	1.51E+08	43904490	59664912	5	1.7E+08	1.08E+08	3.8E+08	26617440	170920685
NS	NR3C1_1	0.01	6	7210040	45764276	98126947	19588485	42672437	6	1.37E+08	96485952	3.94E+08	16154165	160814079
NS	NR3C1_1	0.1	7	2154870	36921212	47660663	25696545	28108322	7	1.82E+08	1.86E+08	4.39E+08	24806420	208107346
NS	NR3C1_1	1	8	1617820	21615768	12280059	6509685	10505833	8	1.05E+08	1.38E+08	3.87E+08	9724170	159723769
NS	NR3C1_1	10	9	1394490	20736179	11417269	4740435	9572093	9	48317404	1.66E+08	4.45E+08	6501295	166325069

				Fold Values				
				GR				
Treatment	siRNA	Concentration (nM)	Lane	N = 1	N = 2	N = 3	N = 4	N = 5
NS	-	-	1	1	1	1	1	1
NS	CTL	0.01	2	1.529588	0.824547	0.93288	0.623968	0.835855
NS	CTL	0.1	3	1.520872	0.969058	0.680307	0.5855	0.844282
NS	CTL	1	4	0.519577	0.881486	0.67	0.727409	0.687546
NS	CTL	10	5	0.894466	0.199776	0.713347	0.601675	0.558018
NS	NR3C1_1	0.01	6	0.34277	0.495762	0.447327	0.442319	0.424177
NS	NR3C1_1	0.1	7	0.076735	0.207279	0.19498	0.377859	0.215909
NS	NR3C1_1	1	8	0.100334	0.164274	0.057003	0.244189	0.105144
NS	NR3C1_1	10	9	0.187558	0.130853	0.046089	0.265973	0.091997

Supplemental Fig. S7B
# - A549 cells: protein

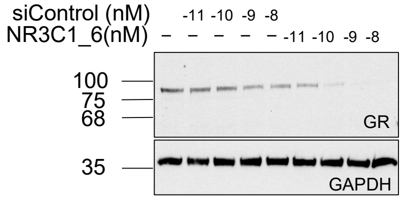

Chemiluminescence

				Adj. Total Band Vol.						Adj. Total Band Vol.				
				GR						GAPDH				
Treatment	siRNA	Concentration (nM)	Lane	N = 1	N = 2	N = 3	N = 4	N = 5	Lane	N = 1	N = 2	N = 3	N = 4	N = 5
NS	-	-	1	26562010	2.15E+08	2.29E+08	45592368	129198049	1	1.73E+08	2.25E+08	4.12E+08	16630725	206528548
NS	CTL	0.01	2	61078110	1.28E+08	1.76E+08	46993457	102984238	2	2.59E+08	1.62E+08	3.39E+08	27472235	196953706
NS	CTL	0.1	3	31763690	1.63E+08	1.52E+08	44394564	97728927	3	1.36E+08	1.76E+08	4.01E+08	27658075	185037469
NS	CTL	1	4	22448690	2.12E+08	1.53E+08	22391152	102561147	4	2.81E+08	2.52E+08	4.1E+08	11228370	238454350
NS	CTL	10	5	23360870	20584810	1.51E+08	43904490	59664912	5	1.7E+08	1.08E+08	3.8E+08	26617440	170920685
NS	NR3C1_1	0.01	6	7210040	45764276	98126947	19588485	42672437	6	1.37E+08	96485952	3.94E+08	16154165	160814079
NS	NR3C1_1	0.1	7	2154870	36921212	47660663	25696545	28108322	7	1.82E+08	1.86E+08	4.39E+08	24806420	208107346
NS	NR3C1_1	1	8	1617820	21615768	12280059	6509685	10505833	8	1.05E+08	1.38E+08	3.87E+08	9724170	159723769
NS	NR3C1_1	10	9	1394490	20736179	11417269	4740435	9572093	9	48317404	1.66E+08	4.45E+08	6501295	166325069

				Fold Values				
				GR				
Treatment	siRNA	Concentration (nM)	Lane	N = 1	N = 2	N = 3	N = 4	N = 5
NS	-	-	1	1	1	1	1	1
NS	CTL	0.01	2	1.529588	0.824547	0.93288	0.623968	0.835855
NS	CTL	0.1	3	1.520872	0.969058	0.680307	0.5855	0.844282
NS	CTL	1	4	0.519577	0.881486	0.67	0.727409	0.687546
NS	CTL	10	5	0.894466	0.199776	0.713347	0.601675	0.558018
NS	NR3C1_1	0.01	6	0.34277	0.495762	0.447327	0.442319	0.424177
NS	NR3C1_1	0.1	7	0.076735	0.207279	0.19498	0.377859	0.215909
NS	NR3C1_1	1	8	0.100334	0.164274	0.057003	0.244189	0.105144
NS	NR3C1_1	10	9	0.187558	0.130853	0.046089	0.265973	0.091997

Supplemental Fig. S7B - NF-κB activity

			Fold/NS				
Treatment	siRNA	Concentration (nM)	N = 1	N = 2	N = 3	N = 4	N = 5
NS	-	-	1	1	1	1	1
Dex	-	-	17.02583	14.1022	9.610609	13.60643	11.73792
Dex	CTL	0.01	18.85029	17.1568	8.699015	13.93155	12.25042
Dex	CTL	0.1	19.13052	17.07701	9.523978	12.51374	13.7636
Dex	CTL	1	15.5734	11.42001	6.901502	12.13887	11.90642
Dex	CTL	10	14.11947	7.111341	6.431616	9.472648	10.80323
Dex	NR3C1_1	0.01	10.7264	7.307002	5.3026	6.88425	5.019205
Dex	NR3C1_1	0.1	5.046221	1.369467	1.270467	1.80444	1.408322
Dex	NR3C1_1	1	1.621961	1.16713	1.111093	1.473044	1.173449
Dex	NR3C1_1	10	1.821557	1.326234	0.990635	1.51361	0.969212
Dex	NR3C1_4	0.01	11.86986	10.45093	4.791539	7.254228	4.150129
Dex	NR3C1_4	0.1	3.945255	1.178892	1.41999	1.659884	1.245389
Dex	NR3C1_4	1	2.394267	0.927283	0.893105	1.234276	0.704618
Dex	NR3C1_4	10	1.530088	0.66121	0.707412	1.390592	0.67505
Dex	NR3C1_5	0.01	15.92536	10.7588	9.806232	12.21948	9.942539
Dex	NR3C1_5	0.1	8.467386	3.99094	2.546262	6.558007	4.010517
Dex	NR3C1_5	1	3.693124	0.616387	0.70289	1.948335	0.740131
Dex	NR3C1_5	10	1.450085	0.522928	0.503149	0.8088	0.36153
Dex	NR3C1_6	0.01	16.94677	11.7619	6.582593	12.26044	3.4984
Dex	NR3C1_6	0.1	4.285534	1.320035	1.646536	2.76731	0.651425
Dex	NR3C1_6	1	1.97449	0.964635	0.711933	1.226084	0.373876
Dex	NR3C1_6	10	2.00783	0.862751	0.817375	1.301004	0.236397

Supplemental Fig. S8 -
A549 cells: protein

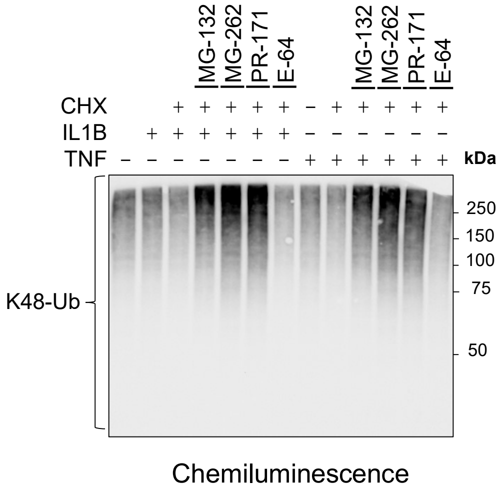

Supplemental Fig. S8 - A549 cells: protein

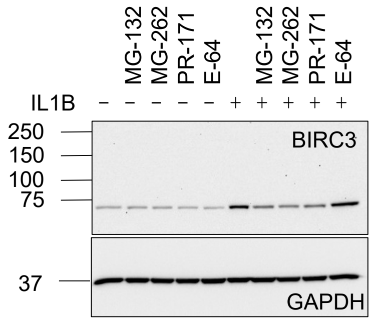

Chemiluminescence

			Adj. Total Band Vol.				Adj. Total Band Vol.		
			BIRC3				GAPDH		
Treatment	Inhibitor	Lane	N = 1	N = 2	N = 3	Lane	N = 1	N = 2	N = 3
NS	-	1	262038	52350454	39233310	1	8720418	91443800	2.06E+08
NS	MG-132	2	278103	40309724	27651876	2	6136832	77353185	1.65E+08
NS	MG-262	3	387532	37942316	30415099	3	6230560	83598270	1.36E+08
NS	PR-171	4	302826	30180160	25661790	4	5622016	77440545	1.19E+08
NS	E-64	5	197907	31024796	27770358	5	6278016	83301192	1.19E+08
IL1B	-	6	499913	1.44E+08	44963037	6	6150752	84273420	1.12E+08
IL1B	MG-132	7	483923	69084328	37059763	7	5908992	93221940	1.36E+08
IL1B	MG-262	8	657353	54896604	47288734	8	6321888	82666710	1.37E+08
IL1B	PR-171	9	374002	60822228	43698900	9	6923680	76664784	1.37E+08
IL1B	E-64	10	796039	1.39E+08	47687913	10	8822257	90090560	1.44E+08

			Normalized Values		
			BIRC3		
Treatment	Inhibitor	Lane	N = 1	N = 2	N = 3
NS	-	1	0.030049	0.572488	0.190901
NS	MG-132	2	0.045317	0.521113	0.167727
NS	MG-262	3	0.062199	0.453865	0.22314
NS	PR-171	4	0.053864	0.38972	0.214855
NS	E-64	5	0.031524	0.372441	0.233824
IL1B	-	6	0.081277	1.710817	0.40065
IL1B	MG-132	7	0.081896	0.741074	0.272884
IL1B	MG-262	8	0.10398	0.664071	0.346339
IL1B	PR-171	9	0.054018	0.793353	0.320109
IL1B	E-64	10	0.090231	1.541269	0.33184

Supplemental Fig. S8 - A549 cells: protein

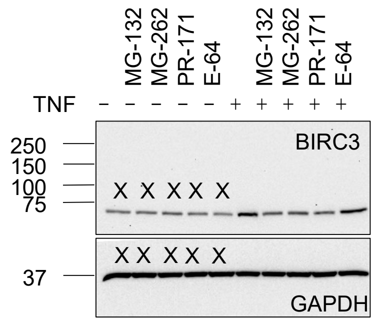

Chemiluminescence

			Adj. Total Band Vol.						Adj. Total Band Vol.				
			BIRC3						GAPDH				
Treatment	Inhibitor	Lane	N = 1	N = 2	N = 3	N = 4	N = 5	Lane	N = 1	N = 2	N = 3	N = 4	N = 5
NS	-	1	7655130	36413682	95010336	13102615	9936648	1	87539004	2.65E+08	1.16E+08	1.41E+08	1.19E+08
NS	MG-132	2	6863019	33534936	80599981	10372445	13571012	2	68212584	1.94E+08	95934720	1.26E+08	84857952
NS	MG-262	3	5783322	30945935	55221229	12886080	12299756	3	59739552	1.53E+08	1.12E+08	1.17E+08	91285272
NS	PR-171	4	5118634	23145365	53778186	7096675	4686227	4	60902820	1.31E+08	1.18E+08	1.17E+08	78218136
NS	E-64	5	6121428	34806474	76029674	9019915	12522419	5	72675468	2.08E+08	1.26E+08	1.3E+08	1.31E+08
TNF	-	6	5542008	27639836	49329273	35359995	68841507	6	56490696	1.85E+08	96578605	1.28E+08	1.03E+08
TNF	MG-132	7	2456319	11141405	36527197	16355445	25810071	7	67857372	1.91E+08	99885555	1.24E+08	77470904
TNF	MG-262	8	4829166	22834578	54773649	15293075	29584394	8	79080948	2.3E+08	1.07E+08	1.51E+08	1.17E+08
TNF	PR-171	9	2544009	14149576	40237603	13679060	22652861	9	75461275	1.79E+08	1.05E+08	1.38E+08	1.19E+08
TNF	E-64	10	10744020	44747800	1.27E+08	31941855	62449476	10	58446547	1.88E+08	1.07E+08	1.32E+08	1.08E+08

			Normalized Values				
			BIRC3				
Treatment	Inhibitor	Lane	N = 1	N = 2	N = 3	N = 4	N = 5
NS	-	1	0.087448	0.137195	0.818499	0.093128	0.083472
NS	MG-132	2	0.100612	0.17284	0.840154	0.082005	0.159926
NS	MG-262	3	0.096809	0.202287	0.495187	0.11041	0.13474
NS	PR-171	4	0.084046	0.176671	0.455673	0.060803	0.059912
NS	E-64	5	0.08423	0.167589	0.601604	0.069609	0.095382
TNF	-	6	0.098105	0.149454	0.510768	0.27571	0.670976
TNF	MG-132	7	0.036198	0.058302	0.36569	0.131387	0.333158
TNF	MG-262	8	0.061066	0.099395	0.512802	0.101053	0.253683
TNF	PR-171	9	0.033713	0.078956	0.382079	0.098933	0.190098
TNF	E-64	10	0.183826	0.237685	1.18502	0.241504	0.576941

Supplemental Fig. S8 - A549 cells: protein

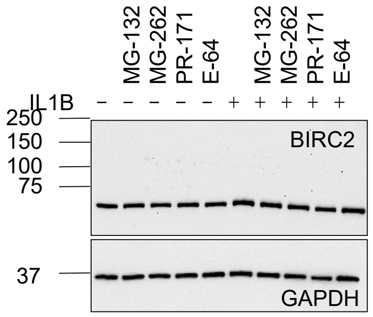

Chemiluminescence

			Adj. Total Band Vol.				Adj. Total Band Vol.		
			BIRC2				GAPDH		
Treatment	Inhibitor	Lane	N = 1	N = 2	N = 3	Lane	N = 1	N = 2	N = 3
NS	-	1	3332910	1.6E+08	1.6E+08	1	4127292	1.58E+08	1.91E+08
NS	MG-132	2	1736720	1.36E+08	1.36E+08	2	3575635	1.94E+08	1.84E+08
NS	MG-262	3	2223770	1.12E+08	1.12E+08	3	3601455	96834255	1.61E+08
NS	PR-171	4	3324520	1.4E+08	1.4E+08	4	3481533	89999644	1.74E+08
NS	E-64	5	4327146	1.45E+08	1.45E+08	5	2995278	77487690	1.98E+08
IL1B	-	6	4195022	1.89E+08	1.89E+08	6	2790124	58798810	2.09E+08
IL1B	MG-132	7	3838362	1.56E+08	1.56E+08	7	2706393	1.13E+08	1.75E+08
IL1B	MG-262	8	2622945	1.43E+08	1.43E+08	8	3469520	1.07E+08	1.44E+08
IL1B	PR-171	9	2613678	1.26E+08	1.26E+08	9	3239903	1.16E+08	1.06E+08
IL1B	E-64	10	1153614	1.89E+08	1.89E+08	10	3036331	1.63E+08	2.32E+08

			Normalized Values		
			BIRC2		
Treatment	Inhibitor	Lane	N = 1	N = 2	N = 3
NS	-	1	0.807529	1.013108	0.842343
NS	MG-132	2	0.48571	0.701792	0.739591
NS	MG-262	3	0.617464	1.156062	0.69347
NS	PR-171	4	0.954901	1.560201	0.808278
NS	E-64	5	1.444656	1.865153	0.729615
IL1B	-	6	1.503525	3.213871	0.903816
IL1B	MG-132	7	1.418257	1.387055	0.894447
IL1B	MG-262	8	0.755997	1.335672	0.987928
IL1B	PR-171	9	0.806715	1.090369	1.188772
IL1B	E-64	10	0.379937	1.157263	0.812073

Supplemental Fig. S8 - A549 cells: protein

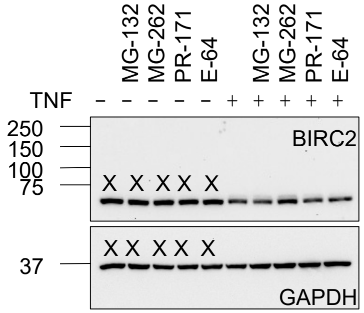

Chemiluminescence

			Adj. Total Band Vol.						Adj. Total Band Vol.				
			BIRC2						GAPDH				
Treatment	Inhibitor	Lane	N = 1	N = 2	N = 3	N = 4	N = 5	Lane	N = 1	N = 2	N = 3	N = 4	N = 5
NS	-	1	93478266	1.3E+08	1.05E+08	89474232	67089528	1	2.05E+08	2.14E+08	1.57E+08	91111536	1.01E+08
NS	MG-132	2	81280719	1.26E+08	74574032	72729190	60905816	2	1.19E+08	1.72E+08	1.31E+08	86074228	70802155
NS	MG-262	3	65067873	1.13E+08	56144107	73147999	61403811	3	87963753	1.23E+08	86727996	86180532	77079184
NS	PR-171	4	68790267	99311730	45578941	53276697	48092754	4	86812752	1.3E+08	59198496	74878786	57178796
NS	E-64	5	1.02E+08	1.18E+08	51642668	77870716	77130130	5	1.44E+08	1.09E+08	47352496	89953569	89656455
TNF	-	6	39959169	45991910	25691747	36822312	53003930	6	1.15E+08	1.06E+08	45039733	95647628	91527022
TNF	MG-132	7	34771680	46134820	22133309	32899043	49442341	7	1.22E+08	1.49E+08	44867625	85297786	74712961
TNF	MG-262	8	56178811	71941700	27708250	47227462	56719644	8	1.48E+08	1.87E+08	59309988	1.09E+08	1.06E+08
TNF	PR-171	9	27701297	45087950	20122286	44483763	56998713	9	1.11E+08	1.67E+08	49754595	1.03E+08	1.17E+08
TNF	E-64	10	43978197	50351440	41507780	40173328	44412138	10	1.56E+08	2.32E+08	91991424	1.1E+08	1.12E+08

			Normalized Values				
			BIRC2				
Treatment	Inhibitor	Lane	N = 1	N = 2	N = 3	N = 4	N = 5
NS	-	1	0.457015	0.607644	0.672446	0.98203	0.665658
NS	MG-132	2	0.68339	0.730209	0.569285	0.844959	0.860225
NS	MG-262	3	0.739712	0.919933	0.647359	0.848776	0.796633
NS	PR-171	4	0.792398	0.764214	0.769934	0.711506	0.841094
NS	E-64	5	0.710724	1.083381	1.090601	0.865677	0.860285
TNF	-	6	0.346863	0.434322	0.570424	0.384979	0.579107
TNF	MG-132	7	0.284392	0.310162	0.493302	0.385696	0.661764
TNF	MG-262	8	0.37951	0.384888	0.467177	0.432693	0.537564
TNF	PR-171	9	0.248877	0.270413	0.404431	0.432735	0.488845
TNF	E-64	10	0.281735	0.217071	0.451214	0.366167	0.396719

Supplemental Fig. S8C – NF-κB Activity

			Fold / NS					
Treatment	Inhibitor	log [inhibitor (µM)]	N = 1	N = 2	N = 3	N = 4	N = 5	N = 6
NS	-	-	1	1	1	1	1	1
IL1B	-	-	4.708723	6.47442	6.867011	6.046266	6.977768	5.024111
IL1B	MG-132	-1	4.941871	5.72405	7.151497	6.374072	6.79588	4.848115
IL1B	MG-132	-0.5	4.804415	5.884325	5.970082	6.371407	6.649887	4.624853
IL1B	MG-132	0	4.474289	6.309595	7.027902	6.257987	7.303838	4.440434
IL1B	MG-132	0.5	3.831677	5.545224	7.135584	6.038661	6.997043	3.42875
IL1B	MG-132	1	2.489992	3.257518	5.606612	3.801628	5.1403	1.870742
IL1B	MG-132	1.5	1.797047	2.408389	4.147745	2.496156	3.507439	1.392308
IL1B	MG-132	2	0.920281	1.207016	1.716671	1.317238	1.982098	0.799466
IL1B	MG-262	-4.5	6.869949	6.296153	7.001707	4.653059		
IL1B	MG-262	-4	7.087909	6.256181	7.151922	4.902817		
IL1B	MG-262	-3.5	6.94068	6.51312	6.413859	4.732443		
IL1B	MG-262	-3	7.343587	5.424629	6.507675	4.496348		
IL1B	MG-262	-2.5	7.14948	3.125579	5.610969	5.107373		
IL1B	MG-262	-2	6.998046	1.747707	2.242859	4.297589		
IL1B	MG-262	-1.5	5.097741	1.38939	1.607088	2.758632		
IL1B	MG-262	-1	2.253068	0.903515	0.963976	1.751136		
IL1B	MG-262	-0.5	1.520078	0.778956	0.922716	1.354615		
IL1B	MG-262	0	1.084908	0.694368	0.857368	0.932696		
IL1B	PR-171	-2.5	3.97181	6.914701	6.320937	5.655459	4.774503	
IL1B	PR-171	-2	6.032681	6.870121	5.579885		5.069258	
IL1B	PR-171	-1.5	4.980415	6.417788	6.759807		4.824391	
IL1B	PR-171	-1	2.693509	5.614334	5.997437	6.771335	3.848524	
IL1B	PR-171	-0.5	3.15207	4.721641	5.484405	6.089956	2.830877	
IL1B	PR-171	0	2.290081	2.811379	4.135539	5.885153	1.547181	
IL1B	PR-171	0.5	1.612771	1.675014	2.79774	3.445572	1.076115	
IL1B	PR-171	1	0.93786	0.918656	1.255876	1.689608	0.917961	
IL1B	PR-171	1.5	0.90485	0.815631	0.905466	0.970199	0.755542	
IL1B	PR-171	2	0.699175	0.677843	0.643562	0.534441	0.698452	
IL1B	E-64	-1	4.941331	6.525192	7.565315	6.311078	7.123171	5.05013
IL1B	E-64	-0.5	5.017464	6.240261	7.534052	6.216574	7.086716	5.426273
IL1B	E-64	0	4.867452	6.211694	1.046753	6.520255	7.449154	5.512756
IL1B	E-64	0.5	5.07916	6.578892	1.056522	6.145863	6.983826	5.138082
IL1B	E-64	1	4.898356	6.003245	7.343884	6.036549	6.744254	5.192196
IL1B	E-64	1.5	4.579053	6.356711	8.167659	6.380799	6.360689	5.32291
IL1B	E-64	2	4.801267	6.933534	8.45796	6.801832	6.570833	5.14084

			Fold / NS					
Treatment	Inhibitor	log [inhibitor (µM)]	N = 1	N = 2	N = 3	N = 4	N = 5	N = 6
NS	-	-	1	1	1	1	1	1
TNF	-	-	3.261174	5.987113	7.507963	6.561426	8.203646	5.529692
TNF	MG-132	-1	2.810251	6.113109	7.142419	6.034705	7.508446	5.680562
TNF	MG-132	-0.5	3.46361	6.062745	6.865876	5.767234	7.118897	5.752371
TNF	MG-132	0	3.057458	5.963133	7.521768	6.246317	6.973552	5.183092
TNF	MG-132	0.5	3.008781	4.978349	7.23299	6.002549	6.332902	4.51648
TNF	MG-132	1	2.440297	3.444375	6.323409	4.537873	5.684983	3.36271
TNF	MG-132	1.5	1.952848	3.009571	5.410391	3.059004	4.726286	2.273678
TNF	MG-132	2	1.270482	1.803703	2.752391	2.195651	2.443067	1.670129
TNF	MG-262	-4.5	6.913568	6.319945	7.01213	5.870467		
TNF	MG-262	-4	7.309477	6.092769	6.886588	5.95401		
TNF	MG-262	-3.5	6.903685	6.361253	7.198509	5.874016		
TNF	MG-262	-3	7.302909	6.177712	6.394949	5.74392		
TNF	MG-262	-2.5	7.257569	3.953364	6.106212	6.068683		
TNF	MG-262	-2	7.703171	2.460021	3.308119	5.103169		
TNF	MG-262	-1.5	6.653478	1.862543	2.675023	3.48906		
TNF	MG-262	-1	2.808622	1.206464	1.667957	2.379223		
TNF	MG-262	-0.5	2.507852	1.065528	1.43664	1.891868		
TNF	MG-262	0	1.8398	0.938785	1.281739	1.648775		
TNF	PR-171	-2.5	5.522731	6.890058	6.538951	6.670194	5.747131	
TNF	PR-171	-2	6.122192	7.23438	6.064728	6.681534	5.845014	
TNF	PR-171	-1.5	3.065835	7.238948	6.221689	6.850903	5.405057	
TNF	PR-171	-1	4.745023	6.578152	6.130888	6.60315	4.931421	
TNF	PR-171	-0.5	3.95412	5.965064	5.691847	5.715266	3.464846	
TNF	PR-171	0	2.852185	5.531178	4.457745	3.817474	2.249324	
TNF	PR-171	0.5	2.061119	2.684275	3.411372	2.657071	1.697948	
TNF	PR-171	1	1.333705	1.79246	1.708355	1.613126	0.865053	
TNF	PR-171	1.5	1.067647	1.321866	1.299174	1.270147	0.751113	
TNF	PR-171	2	0.617952	0.851624	0.962562	0.868535	0.98336	
TNF	E-64	-1	3.596446	6.078496	7.655495	6.250058	6.569316	6.124885
TNF	E-64	-0.5	3.480344	6.074802	7.867878	6.776785	7.27723	5.858663
TNF	E-64	0	3.248478	6.009083	7.445724	6.572552	7.027003	5.701549
TNF	E-64	0.5	3.550988	5.739773	7.696954	6.791618	6.798513	5.709339
TNF	E-64	1	3.605613	5.387809	7.603006	6.439741	6.981022	5.613964
TNF	E-64	1.5	3.439196	5.814621	7.260075	6.340487	6.445843	5.213729
TNF	E-64	2	3.193758	5.687202	6.357062	5.53991	6.01674	4.282754
