# Supplementary material for: Differential regulation of BIRC2 and BIRC3 expression by inflammatory cytokines and glucocorticoids in pulmonary epithelial cells
Source: PLoS One. 2023 Jun 8;18(6):e0286783. doi: 10.1371/journal.pone.0286783 (PMC10249814; doi:10.1371/journal.pone.0286783)
Supplement: Attachment — Submitted filename: Response to Reviewers.docx [file pone.0286783.s004.docx]

[△EXTERNAL]

PONE-D-22-30303

Differential regulation of BIRC2 and BIRC3 expression by inflammatory cytokines and glucocorticoids in pulmonary epithelial cells

PLOS ONE

Dear Dr. Newton,

Thank you for submitting your manuscript to PLOS ONE. After careful consideration, we feel that it has merit but does not fully meet PLOS ONE’s publication criteria as it currently stands. Therefore, we invite you to submit a revised version of the manuscript that addresses the points raised during the review process.

I have assessed your submission, and I agree with the reviewer's comments about the text fluency of the manuscript. I, therefore, request that, besides addressing all the points raised by the reviewers, you revise the text to improve the overall readability of the text, providing fluency and avoiding repetition.

Author response: In response to reviewer 1, and reiterated above, we have carefully reviewed the whole manuscript to try and improve the clarity. As per the request of the reviewer, we have introduced introductory sentences to explain more fully the rationale underlying experiments. We have also included sentences to sum up key findings at the end of each part of the results section. We have also sought to address each of the comments raised by both reviewers. As may have been expected, this has resulted in a longer manuscript. In revising the manuscript, we have also made additional changes to improve the readability and, where possible, to reduce length. Additionally, we noticed some minor errors in the figures. Figure 2C has had the cell type indication on the y-axis relabelled. Figure 4B has had the number of experiments correctly stated as *N* = 9 instead of *N* = 10. As is noted below, the lane indicators for Figure 5D were wrong and have now been corrected.

We hope that the revised version meets your approval. We are very happy to make further changes that might be deemed necessary.

Please submit your revised manuscript by Mar 24 2023 11:59PM. If you will need more time than this to complete your revisions, please reply to this message or contact the journal office at [plosone@plos.org](mailto:plosone@plos.org). When you're ready to submit your revision, log on to <https://www.editorialmanager.com/pone/> and select the 'Submissions Needing Revision' folder to locate your manuscript file.

Please include the following items when submitting your revised manuscript:

- A rebuttal letter that responds to each point raised by the academic editor and reviewer(s). You should upload this letter as a separate file labeled 'Response to Reviewers'.
- A marked-up copy of your manuscript that highlights changes made to the original version. You should upload this as a separate file labeled 'Revised Manuscript with Track Changes'.
- An unmarked version of your revised paper without tracked changes. You should upload this as a separate file labeled 'Manuscript'.

If you would like to make changes to your financial disclosure, please include your updated statement in your cover letter. Guidelines for resubmitting your figure files are available below the reviewer comments at the end of this letter.

If applicable, we recommend that you deposit your laboratory protocols in protocols.io to enhance the reproducibility of your results. Protocols.io assigns your protocol its own identifier (DOI) so that it can be cited independently in the future. For instructions see: [https://journals.plos.org/plosone/s/submission-guidelines#loc-laboratory-protocols](https://can01.safelinks.protection.outlook.com/?url=https%3A%2F%2Fjournals.plos.org%2Fplosone%2Fs%2Fsubmission-guidelines%23loc-laboratory-protocols&data=05%7C01%7Crnewton%40ucalgary.ca%7C1295965c64b54bb5923b08db09271d5c%7Cc609a0eca5e346319686192280bd9151%7C1%7C0%7C638113835954299683%7CUnknown%7CTWFpbGZsb3d8eyJWIjoiMC4wLjAwMDAiLCJQIjoiV2luMzIiLCJBTiI6Ik1haWwiLCJXVCI6Mn0%3D%7C3000%7C%7C%7C&sdata=jNYLnT%2FTdgSSRQjVtweMGtWQ9uVF0SUllKIiEsmXlyM%3D&reserved=0). Additionally, PLOS ONE offers an option for publishing peer-reviewed Lab Protocol articles, which describe protocols hosted on protocols.io. Read more information on sharing protocols at [https://plos.org/protocols?utm_medium=editorial-email&utm_source=authorletters&utm_campaign=protocols](https://can01.safelinks.protection.outlook.com/?url=https%3A%2F%2Fplos.org%2Fprotocols%3Futm_medium%3Deditorial-email%26utm_source%3Dauthorletters%26utm_campaign%3Dprotocols&data=05%7C01%7Crnewton%40ucalgary.ca%7C1295965c64b54bb5923b08db09271d5c%7Cc609a0eca5e346319686192280bd9151%7C1%7C0%7C638113835954299683%7CUnknown%7CTWFpbGZsb3d8eyJWIjoiMC4wLjAwMDAiLCJQIjoiV2luMzIiLCJBTiI6Ik1haWwiLCJXVCI6Mn0%3D%7C3000%7C%7C%7C&sdata=PTqL%2F14JXYy3mUs2jE6gNFKs6Wx8aInNt8wz1oGTMtQ%3D&reserved=0).

We look forward to receiving your revised manuscript.

Kind regards,

Aristóbolo M Silva
Academic Editor
PLOS ONE

Journal Requirements:

When submitting your revision, we need you to address these additional requirements.

1. Please ensure that your manuscript meets PLOS ONE's style requirements, including those for file naming. The PLOS ONE style templates can be found at

<https://journals.plos.org/plosone/s/file?id=wjVg/PLOSOne_formatting_sample_main_body.pdf> and

[https://journals.plos.org/plosone/s/file?id=ba62/PLOSOne_formatting_sample_title_authors_affiliations.pdf](https://can01.safelinks.protection.outlook.com/?url=https%3A%2F%2Fjournals.plos.org%2Fplosone%2Fs%2Ffile%3Fid%3Dba62%2FPLOSOne_formatting_sample_title_authors_affiliations.pdf&data=05%7C01%7Crnewton%40ucalgary.ca%7C1295965c64b54bb5923b08db09271d5c%7Cc609a0eca5e346319686192280bd9151%7C1%7C0%7C638113835954299683%7CUnknown%7CTWFpbGZsb3d8eyJWIjoiMC4wLjAwMDAiLCJQIjoiV2luMzIiLCJBTiI6Ik1haWwiLCJXVCI6Mn0%3D%7C3000%7C%7C%7C&sdata=XcIely1DL1oVm8d8MQwbRyVKFOmwZalO2dUEtyR6Jgs%3D&reserved=0)

2. PLOS ONE now requires that authors provide the original uncropped and unadjusted images underlying all blot or gel results reported in a submission’s figures or Supporting Information files. This policy and the journal’s other requirements for blot/gel reporting and figure preparation are described in detail at <https://journals.plos.org/plosone/s/figures#loc-blot-and-gel-reporting-requirements> and <https://journals.plos.org/plosone/s/figures#loc-preparing-figures-from-image-files>. When you submit your revised manuscript, please ensure that your figures adhere fully to these guidelines and provide the original underlying images for all blot or gel data reported in your submission. See the following link for instructions on providing the original image data: <https://journals.plos.org/plosone/s/figures#loc-original-images-for-blots-and-gels>.

Author response: With the revised manuscript, we have now included an PDF file of Supporting Information that contains all the raw data that is presented in the manuscript. This includes tables with all the underlying data for all experiments that are presented in the manuscript. In addition, to tabulation of all the data, we have provided the original, uncropped, images for all representative western blot gel images that are shown in the manuscript. All these data are to be found as Supporting Information file:

S9 Fig. Raw data and uncropped western blot images

In your cover letter, please note whether your blot/gel image data are in Supporting Information or posted at a public data repository, provide the repository URL if relevant, and provide specific details as to which raw blot/gel images, if any, are not available. Email us at [plosone@plos.org](mailto:plosone@plos.org) if you have any questions.

3. Please include captions for your Supporting Information files at the end of your manuscript, and update any in-text citations to match accordingly. Please see our Supporting Information guidelines for more information: [http://journals.plos.org/plosone/s/supporting-information](https://can01.safelinks.protection.outlook.com/?url=http%3A%2F%2Fjournals.plos.org%2Fplosone%2Fs%2Fsupporting-information&data=05%7C01%7Crnewton%40ucalgary.ca%7C1295965c64b54bb5923b08db09271d5c%7Cc609a0eca5e346319686192280bd9151%7C1%7C0%7C638113835954299683%7CUnknown%7CTWFpbGZsb3d8eyJWIjoiMC4wLjAwMDAiLCJQIjoiV2luMzIiLCJBTiI6Ik1haWwiLCJXVCI6Mn0%3D%7C3000%7C%7C%7C&sdata=FrOAKTIykZg5GPUJcXNiFxmvULuAEeNarkcdAfae2BU%3D&reserved=0).

Author response: Captions for all Supporting Information files are now included at the end of the manuscript.

4. Please review your reference list to ensure that it is complete and correct. If you have cited papers that have been retracted, please include the rationale for doing so in the manuscript text, or remove these references and replace them with relevant current references. Any changes to the reference list should be mentioned in the rebuttal letter that accompanies your revised manuscript. If you need to cite a retracted article, indicate the article’s retracted status in the References list and also include a citation and full reference for the retraction notice.

Author response: The reference list has been reviewed for accuracy and completeness. In making changes to the manuscript to address the reviewer comments, we have added additional references. These are now included in the revised version of the manuscript. There are no references to retracted articles.

Reviewers' comments:

Reviewer's Responses to Questions

**Comments to the Author**

**Reviewer 1**

1. Is the manuscript technically sound, and do the data support the conclusions?

The manuscript must describe a technically sound piece of scientific research with data that supports the conclusions. Experiments must have been conducted rigorously, with appropriate controls, replication, and sample sizes. The conclusions must be drawn appropriately based on the data presented.

Reviewer #1: Yes

Reviewer #2: Yes

2. Has the statistical analysis been performed appropriately and rigorously?

Reviewer #1: Yes

Reviewer #2: Yes

3. Have the authors made all data underlying the findings in their manuscript fully available?

The [PLOS Data policy](https://can01.safelinks.protection.outlook.com/?url=http%3A%2F%2Fwww.plosone.org%2Fstatic%2Fpolicies.action%23sharing&data=05%7C01%7Crnewton%40ucalgary.ca%7C1295965c64b54bb5923b08db09271d5c%7Cc609a0eca5e346319686192280bd9151%7C1%7C0%7C638113835954299683%7CUnknown%7CTWFpbGZsb3d8eyJWIjoiMC4wLjAwMDAiLCJQIjoiV2luMzIiLCJBTiI6Ik1haWwiLCJXVCI6Mn0%3D%7C3000%7C%7C%7C&sdata=457kadYAL1xo%2FtfVlhGDfyEMojENElC6tn1bWpmWo60%3D&reserved=0) requires authors to make all data underlying the findings described in their manuscript fully available without restriction, with rare exception (please refer to the Data Availability Statement in the manuscript PDF file). The data should be provided as part of the manuscript or its supporting information, or deposited to a public repository. For example, in addition to summary statistics, the data points behind means, medians and variance measures should be available. If there are restrictions on publicly sharing data—e.g. participant privacy or use of data from a third party—those must be specified.

Reviewer #1: No

Reviewer #2: Yes

Author response: As requested, we now also provide all the underlying values for all data in all figures in a new Supplemental information file (S9 Fig. Raw data and uncropped western blot images). We trust that this meets with your approval. We are very happy to take onboard further suggestions and are very happy for all our underlying data to be freely available.

4. Is the manuscript presented in an intelligible fashion and written in standard English?

PLOS ONE does not copyedit accepted manuscripts, so the language in submitted articles must be clear, correct, and unambiguous. Any typographical or grammatical errors should be corrected at revision, so please note any specific errors here.

Reviewer #1: No

Reviewer #2: Yes

Author response: We note the request by reviewer 1 (and restated by the Editor) to provide enhanced explanations, or rationales, for experiments within the results sections. The revised manuscript has been extensively revised to adopt this style of presentation. Please feel free to make further suggestions. We have separately reviewed the whole manuscript with a view to improving clarity and language.

5. Review Comments to the Author

Please use the space provided to explain your answers to the questions above. You may also include additional comments for the author, including concerns about dual publication, research ethics, or publication ethics. (Please upload your review as an attachment if it exceeds 20,000 characters)

Reviewer #1: This article aims to study the regulation of the expression of BIRC2 and BIRC3 in bronchial epithelial cell lines cultured in the presence of pro-inflammatory cytokines (IL-1β or TNFα), in the presence or the absence of the anti-inflammatory compounds dexamethasone or budesonide.

The results shown are interesting but the article is very difficult to read and follow. This could be improved by an introduction in result paragraphs specifying the objectives of the experiments carried out. Moreover, concluding sentence at the end of the result paragraphs could help in the understanding of the results.

Author response: Introductory statements and concluding remarks have been added to each component of the results. We hope that this satisfies the concerns of the reviewer.

I did not understand why the authors analyzed the influence of INFγ (Fig S2D). Since INFγ is no longer used in later experiments and these results are not used for discussion, I believe they are not necessary (line 209-214).

Author response: With these BIRC genes possibly being important in cell fate decisions, for example in response to viral infections, we hypothesized that IFNγ, which is often induced with viral infections, may up-regulate BIRC expression. We therefore examined IFNγ as a potential inducer of each gene. This rationale is now explained more clearly in the revised manuscript (See lines 224-226). We also reasoned that IL1B and IFNγ, in combination, may further modulate BIRC gene expression and these possibilities were therefore tested. However, we did not see any major changes in BIRC2 or BIRC3 expression and therefore roles for IFNγ were not examined further in this manuscript. We believe that it is important to provide this information in the event others were to replicate similar lines of thought. The availability of these, albeit largely negative, data is necessary to prevent someone repeating such experiments. Additional comment and discussion of these data has been added to the revised manuscript (lines 454-459).

Figure legends: could author indicate the number of experiments (instead of “N”)

Author response: The number of independent experiments (“*N*”) is indicated within every graph that we have presented in this manuscript. Given the number of different graphs in many of the figures, we felt that this represented the clearest and most efficient way of showing this information is respect of each individual graph. We hope that the reviewer agrees.

Why the authors analyzed the regulation of BIRC2 and BIRC3 expression in pHBEC grown as both submersion culture or as highly differentiated cells? Did they expect different mechanisms of regulation in function of the differentiation state? It seems to me that the results are quite similar, whatever the cell line: A549, pHBEC-SC or pHBEC-ALI and also BEAS-2B and Calu-3. It should be possible to simplify the description of the results in order to avoid repetition and provide fluency (results paragraph 1 and 2).

Author response: Different models of pulmonary epithelial cells are routinely used in the literature and these often show different responses and/or differential gene expression. A number of commonly used models were therefore examined as these may have captured differential patterns of BIRC regulation. However, as the data showed, and the reviewer clearly agrees, the general patterns of BIRC2/3 expression were quite similar across these different cell lines and primary cell models. These are important findings as this validates the use of these cell lines to model the gene expression and/or function that occurs in primary cells. Further, the finding that the relatively undifferentiated primary HBEs in submersion culture closely mimics the outcome in the more highly differentiated air-liquid interface (ALI) cells is reassuring. The cells grown at an ALI take almost 2 months to grow and are only available in very limiting qualities. Knowing that gene expression changes can be largely recapitulated in the simpler models is therefore helpful. As the key aspects of BIRC expression were maintained across all these models, we used A549 cells for the mechanistic aspects of this study. The above points are highlighted in the revised manuscript (lines 305-311). Similarly, the above comparisons do reveal some more subtle differences between the different model systems. We note the reviewer’s point about avoiding repetition and we have revised this section to more concisely state similarities, while highlighting differences between models.

Figure 3D showed that the IL-1β and TNFα-mediated overexpression of BIRC2 mRNA is prevented by IκBαΔN expression whereas it is not affected by PS-1145 (Figure 4A) that blocks NF-κB. Could authors explain these results. Moreover, PS-1145, as well as ML-120B reduced BIRC2 protein expression in TNF-α-treated cells while PS-1145 did not modify the level of expression of the mRNA. These results have to be explained.

Author response: As noted, by the reviewer, Fig 3D shows that IL1B-induced expression of both BIRC3 and BIRC2 mRNAs were prevented by over-expression of IκBαΔN. This dominant inhibitor will inhibit all NF-κB irrespective of the different signaling pathways (e.g. canonical/non-canonical, etc) that may have been involved in the induction of NF-κB activity. By contrast, Fig. 4A, shows BIRC3 and BIRC2 mRNAs to both be induced by IL1B, but only expression of BIRC3 was attenuated by the selective IKK2 inhibitor. These data may be explained by the concept that in addition to canonical activation, via IKK2, there is also activation of NF-κB that may occur via non-canonical, i.e. IKK2-independent, pathways. This possibility is further discussed in the results and in the discussion (See lines 355-361 & 471-473). The apparent discrepancy between the lack of inhibition of PS-1145 on TNF-induced BIRC2 mRNA at 6 h and the loss of BIRC2 protein in the presence of TNF at 24 may be explained by the time difference combined with the possibility of indirect effects. For example, the later loss of BIRC2 maybe a consequence of cell death signaling that can be observed in the context of IKK2/NF-κB inhibition in the presence of TNF. As requested, by the reviewer, this is further discussed (See, lines 471-476).

A concluding sentence concerning the role of NF-κB in BIRC3 and BIRC2 expression at the end of the result paragraph 4 could greatly improve the understanding of the results.

Author response: This has been added (See lines 355-361).

Figure 5D. I think that there are errors in the indications of the presence or not of siGR and siCTL.

Author response: Thank you for bringing this to our attention, this error has been corrected in the revised manuscript.

In the result paragraph 6, the authors analyzed the expression of BIRC3 and BIRC2 proteins in IL-1β or TNFα-treated cells in the presence of absence of cycloheximide. The author should introduce this paragraph and indicate the objectives of these experiments. What is the question addressed? Why did the authors add CHX 1 hours after the cytokine?

Author response: As requested above, introductory statements describing the intent for these experiments have been added (See lines 371 - 382). Briefly, the cells were stimulated with each cytokine for 1 h prior to addition of cycloheximide to allow activation of NF-κB and stimulation of other pathways to occur prior to the addition of CHX and analysis of effects on BIRC expression.

CHX treatment prevented the IL-1β or TNFα-mediated increase of BIRC3 expression and in a lower extend BIRC2 expression and even decreased BIRC2 protein expression in TNFα-stimulated cells. Again, the authors should give the conclusion of the experiments to help in the understanding of the article.

Author response: We agree, the addition of CHX confirmed a need for continuous protein translation for induced BIRC2/3 expression. However, a marked decrease in BIRC protein stability was observed in cells treated with TNF – this revealed a significant reduction in the presence of protein for both BIRCs to a level that was lower than baseline in untreated cells. These data suggest that in TNF-treated cells there is an active loss of BIRC2 and BIRC3 proteins. We have added additional explanation in the revised manuscript (See lines 397-399).

Then, the authors added CHX 6 hours after the cytokine. I did not understand why they did this experiment? What is the question addressed? These points could be precised in the manuscript.

Author response: The first experiment using CHX addressed the stability of the BIRC2 and BIRC3 that is present just 1 h following IL1B or TNF treatment. However, BIRC3 protein expression was also highly induced following both IL1B and TNF treatments. To assess stability of this newly synthesized BIRC3 protein, we selected 6 h for further analysis of protein stability. However, upon CHX treatment, no loss of protein was seen for either IL1B- or TNF-induced BIRC3 expression. Thus, at 6 h, the IL1B- and TNF-induced BIRC3 protein is not subjected to degradation with these stimuli. As requested, we have added in further details to explain our rationale and to discuss these data (See 406-408).

In the last result paragraph, the author analyzed the mechanisms of BIRC2 and BIRC3 degradation? Since the degradation was only observed in the condition of TNFα + CHX treatment (Figure 6B), why did they analyze the condition of IL1β + CHX treatment and why did they analyze the role of proteasome inhibitor on IL1β- or TNFα-induced expression of BIRC3 and BIRC2 ?

Author response: We agree that Fig 6 showed loss of BIRC protein only in the presence of TNF + CHX. However, whereas Fig 6, represented a time kinetic, Fig 8 represents analyses performed at 6 h post CHX addition. We were therefore gratified to find that the data in Fig 6 were fully substantiated in Fig 8. We believe that showing such replication adds robustness to our work. Furthermore, the contrast between the loss of BIRC proteins in the presence of TNF that was blocked by the proteasome inhibitors, with the lack of effect in the presence IL1B provides further robustness to the data. This said, we do agree that the strictly speaking these IL1B data, do not add materially to the story. While we feel that the data work well as one figure, if the reviewer would prefer, we would be very happy to add this part of data to the supplement.

It is well known that NF-κB activation required proteasome-mediated degradation of IκBα. Since IL1β- or TNFα-induced expression of BIRC3 and BIRC2 is dependent on NF-κB, it seems logical that proteasome inhibitors block the induction of BIRC3 and BIRC2 expression.

Author response: We agree with the reviewer, indeed we had specifically noted this point in the manuscript. Nevertheless, what is predicted, and therefore expected, and what actually happens is not always the same thing. Hence, we felt it important confirm the effects of the proteasome inhibitors on the expression of BIRC3 and 2. As shown in Fig. 8B, the proteasome inhibitors prevented the increase in BIRC3 expression when induced by IL1B or TNF. This supports the concept that the proteasome inhibitors block IκB degradation to reduce NF-κB activity (as is shown in Supplemental Fig S8). Thus, BIRC3 expression was reduced. BIRC2 expression was not materially affected by IL1B and TNF (Fig. 8B) and hence the proteasome inhibitors did not show any marked effects on BIRC2 expression. These data are more clearly explained in the revised manuscript (See lines 438-441).

The results show that BIRC2 and BIRC3 proteins is degraded 6-7 hours after stimulation of cells with TNFα but not after IL-1β stimulation. This could argue in a favor of a role of BIRC2 and BIRC3 in TNFR signaling pathway but not in IL-1βR signaling pathway. Since BIRC2 is an important cell signaling intermediate in TNFR signaling pathway, we may hypotheze that the degradation of BIRC2 is a feed-back regulatory mechanism in order to regulate TNFR signaling pathway in case of sustain stimulation of receptor. BIRC2 in an E3-ubiquitine ligase able to self-ubiquitinate and stimulate its own degradation and also able to promote the ubiquitinatation and degradation of BIRC3 (in case of Smac mimetic exposure). This point could be discussed.

Author response: We agree with this as a possible line of thought. Indeed, this concept was presented in the prior version of the manuscript. Based on the reviewer’s comments, we have further highlighted and further discussed this possibility (See lines 491-498).

**Reviewer 2:**

Reviewer #2: In this excellent study, Thorne et al., from Newton’s group sought to dissociate the redundant roles of BIRC2 and BIRC3 in the regulation of inflammatory signaling in the presence or absence of glucocorticoids (GC) in physiologically relevant cells, airway epithelial cells. The authors used both pulmonary epithelial cell lines (A549, BEAS2B, Calu-3 cells) and primary human bronchial epithelial cells (pHBECs cultured either in submersion culture or ALI). To this end, responses to cytokines (TNF, IL1, IFNg) and GC (Dexamethasone, Budesonide) have been examined in detail. The role of NF-kB in BIRCs expression induced by cytokines has been demonstrated using various strategies such as siRNA, DN, and IKK inhibitors. The involvement of the glucocorticoid receptor in BRCs expression induced by GC has been also demonstrated using GR inhibitor or siRNA GR. The involvement of protein degradation in TNF effects on BRCs was explored and demonstrated using CHX and MG compound. The authors clearly demonstrated the differential regulation of BIRC3 and BIRC2 in epithelial cells by inflammatory cytokines and GC where the constitutive expression of BIRC2 is consistent with acute early events and the inducible expression of BIRC3 is more consistent with later onset roles. The authors also demonstrated that BIRC3 expression is induced by GC and suggested that BIRC3 plays key protective roles in inflammatory signaling. Overall, the study is well-designed, comprehensive and the Ms is well-written. Some minor issues are presented below.

Author response: We thank the reviewer for these remarks.

Minor comments:
- While the authors suggested a protective role of BIRC3, some studies showed a correlation between BRC3 and asthma severity (PMID: 27304223). Further discussion is needed.

Author response: This reference was mentioned in the introduction to the prior version of the manuscript. In addition, we have now added further comments in the discussion to indicate the need to further explore this relationship with asthma. The suggested reference can be found in the revised manuscript at lines 87-91 & 511 - 513).

- While most of the supra-additive effects of IL1b and GC were seen at 24 hr (Fig. 2A), most of the western blot studies (Figs 3C, 5B and FC) were conducted at 6 hr where not clear supra-additive effects were observed. Some justification is needed.

Author response: The reviewer is correct, this represents a limitation of the current work. At the outset, we sought to primarily understand direct mechanisms by which NF-κB and GR control BIRC gene expression. At 6 h, we saw robust and often maximum expression for BIRC3 induced by each cytokine alone. It is these effects that we have mostly sought to interrogate. We agree that by 24 h there were additional interactions occurring, but we predict that multiple downstream, potentially indirect, mechanisms may be at play. These may contribute to the observed supra-additivity, but are not primarily addressed in the current manuscript. We have added additional words to explicitly note this as a limitation to the current study. See lines 250-260.

- Figures: Fig 5D, western blot: Experimental conditions under bands, 3, 6, and 9 are not clear. Are those triplicates? Conditions with IL1B only were not presented while stated in the Fig 5D legend.

Author response: Many thanks for pointing out this error. The labelling errors are rectified in the revised manuscript. Hopefully this is now clear!

- Introduction section: Second paragraph, the opposite/contradicting role of BIRCs on the regulation of NF-kB in canonical (line 54-64) versus non-canonical pathways (line 64-67 where the term BRC2/3 inhibition is not clear) is a little bit confusing.

Author response: We note and agree with the reviewers point. Our aim was to highlight that there exists much contradictory evidence as to the roles of BIRC2 and BIRC3, including their possible roles in the different NF-κB-activation pathways. In the revised manuscript, we have tried to clarify this section (See lines 69-72).

- Due the high number of data and variables in this study, the addition of a summary table highlighting all the findings in one place will enhance the clarity of this study.

Author response: We thank the reviewer for this suggestion – in the revised manuscript we have incorporated a table that summarises the various finding of the study. Please see: Supporting information file ‘S1. Table’.

- Similarly, a graph depicting the conclusion of the authors should help reinforcing this critical concept and findings generated by this study.

Author response: Again, we thank the reviewer for this suggestion. However, after various attempts to make a concise figure, we felt that this failed to enhance the clarity. To a large extent, we found that the rather imprecise literature as to the cellular roles of BIRC2 and 3 resulted in a lack of clarity in the resultant figure. Conversely, we felt reluctant to generate a figure that might reenforce an incorrect functional role for each BIRC (see comments above). Therefore, we prefer to stick with the addition of the summary table and not introduce a schematic figure. If the reviewer has further suggestions on this, we would very happily consider these.

6. PLOS authors have the option to publish the peer review history of their article ([what does this mean?](https://can01.safelinks.protection.outlook.com/?url=https%3A%2F%2Fjournals.plos.org%2Fplosone%2Fs%2Feditorial-and-peer-review-process%23loc-peer-review-history&data=05%7C01%7Crnewton%40ucalgary.ca%7C1295965c64b54bb5923b08db09271d5c%7Cc609a0eca5e346319686192280bd9151%7C1%7C0%7C638113835954299683%7CUnknown%7CTWFpbGZsb3d8eyJWIjoiMC4wLjAwMDAiLCJQIjoiV2luMzIiLCJBTiI6Ik1haWwiLCJXVCI6Mn0%3D%7C3000%7C%7C%7C&sdata=AtP6o0TxHYuQiJyEcLMW7kAvMzgawlDW5dawZXTigIE%3D&reserved=0)). If published, this will include your full peer review and any attached files.


If you choose “no”, your identity will remain anonymous but your review may still be made public.


**Do you want your identity to be public for this peer review?** For information about this choice, including consent withdrawal, please see our [Privacy Policy](https://can01.safelinks.protection.outlook.com/?url=https%3A%2F%2Fwww.plos.org%2Fprivacy-policy&data=05%7C01%7Crnewton%40ucalgary.ca%7C1295965c64b54bb5923b08db09271d5c%7Cc609a0eca5e346319686192280bd9151%7C1%7C0%7C638113835954299683%7CUnknown%7CTWFpbGZsb3d8eyJWIjoiMC4wLjAwMDAiLCJQIjoiV2luMzIiLCJBTiI6Ik1haWwiLCJXVCI6Mn0%3D%7C3000%7C%7C%7C&sdata=cc%2FXE0ek8fM1XHMB7CSplgtcx075tv90BYpxmaZBU0w%3D&reserved=0).

Reviewer #1: No

Reviewer #2: No

[NOTE: If reviewer comments were submitted as an attachment file, they will be attached to this email and accessible via the submission site. Please log into your account, locate the manuscript record, and check for the action link "View Attachments". If this link does not appear, there are no attachment files.]

While revising your submission, please upload your figure files to the Preflight Analysis and Conversion Engine (PACE) digital diagnostic tool, [https://pacev2.apexcovantage.com/](https://can01.safelinks.protection.outlook.com/?url=https%3A%2F%2Fpacev2.apexcovantage.com%2F&data=05%7C01%7Crnewton%40ucalgary.ca%7C1295965c64b54bb5923b08db09271d5c%7Cc609a0eca5e346319686192280bd9151%7C1%7C0%7C638113835954299683%7CUnknown%7CTWFpbGZsb3d8eyJWIjoiMC4wLjAwMDAiLCJQIjoiV2luMzIiLCJBTiI6Ik1haWwiLCJXVCI6Mn0%3D%7C3000%7C%7C%7C&sdata=NpR2JWJu6CAULmZUr1AzTo1iC%2FTijUmI5Z2VJfIj8WQ%3D&reserved=0). PACE helps ensure that figures meet PLOS requirements. To use PACE, you must first register as a user. Registration is free. Then, login and navigate to the UPLOAD tab, where you will find detailed instructions on how to use the tool. If you encounter any issues or have any questions when using PACE, please email PLOS at [figures@plos.org](mailto:figures@plos.org). Please note that Supporting Information files do not need this step.

*In compliance with data protection regulations, you may request that we remove your personal registration details at any time.* [*(Remove my information/details)*](https://can01.safelinks.protection.outlook.com/?url=https%3A%2F%2Fwww.editorialmanager.com%2Fpone%2Flogin.asp%3Fa%3Dr&data=05%7C01%7Crnewton%40ucalgary.ca%7C1295965c64b54bb5923b08db09271d5c%7Cc609a0eca5e346319686192280bd9151%7C1%7C0%7C638113835954299683%7CUnknown%7CTWFpbGZsb3d8eyJWIjoiMC4wLjAwMDAiLCJQIjoiV2luMzIiLCJBTiI6Ik1haWwiLCJXVCI6Mn0%3D%7C3000%7C%7C%7C&sdata=uOImaCaKIUvtxstjyX8JB9k4nTNZKUwD8OwKCQBm2sw%3D&reserved=0)*. Please contact the publication office if you have any questions.*
