# Supplementary material for: Differential regulation of BIRC2 and BIRC3 expression by inflammatory cytokines and glucocorticoids in pulmonary epithelial cells
Source: PLoS One. 2023 Jun 8;18(6):e0286783. doi: 10.1371/journal.pone.0286783 (PMC10249814; doi:10.1371/journal.pone.0286783)
Supplement: Attachment — Submitted filename: Response to Reviewers.docx [file pone.0286783.s005.docx]

**Response to reviewer comments**

Reviewer #1: The authors answered to my concerns. However, the article is still difficult to follow. I agree that replication of the results adds robustness but it also brings heaviness and makes the article difficult to read.

Author response: Thanks for these remarks. In addition to specifically addressing the concerns raised below, we have also made various minor modifications to the text of the manuscript with the aim of further improving readability.

1. Figure S1 and Figure 3B both demonstrated that IL-1β and TNFα both induced the activation of NF-κB in the concentrations used in the study. I think that the supplementary Figure 1 can be removed.

Author Response: While both Fig 3B and Fig S1 do indeed show activation of the NF-κB reporter, the data shown in Fig S1 is necessary to first establish the maximal effective concentrations of IL1B and TNF that are then used in all subsequent experiments. This point was not clearly made in the prior version of the manuscript. Sorry! We have now reworked this part to make the importance of these data more readily apparent to the reader. Thus, Fig S1 represents a necessary piece of initial data and provides the necessary rationale for using 1 ng/ml of IL1B and 10 ng/ml of TNF. We therefore prefer to retain this figure as Supplemental Fig S1.

Then, the authors analyzed the contribution of NF-κB in IL-1β and IL-1β + DEX-induced BIR2 and BIRC3 mRNA expression by using RELA-directed siRNA or IκBα super-repressor. They showed that IL-1β and IL-1β + DEX-induced increased of BIRC2 and BIRC3 mRNA are, at least partly, NF-κB dependent. Then, they confirmed that the increase in BIRC3 protein expression also depended on NF-κB (Figure 3 and S6). The same results were observed in Figure 3C and S6. Could authors simplify the text by describing the two Figure in the same sentence. Since IL1B and IL1B + DEX did not enhanced BIRC2 protein expression (as demonstrated in Figure 2), I think that the authors could simplify the Figure 3 and remove the analysis of BIRC2 protein expression.

Author Response: We have condensed this part of the text as requested by the reviewer. We have also removed Figure 3C bottom panel (analysis of BIRC2 protein expression) and have also condensed the relevant parts of the text relating to these data.

2. In Figure 4A, the authors confirmed that NF-κB is involved in the induction of BIRC3 mRNA and protein expression in IL1B, TNFα, IL1B + DEX and TNF + DEX-treated cells by using the IKK2 inhibitor PS-1145. By contrast, PS-1145 did not decrease the induction of BIRC2 mRNA in response to the stimuli. The analysis of BIRC3 protein expression confirmed the involvement of NF-κB. Again, since IL1B and IL1B + DEX did not enhanced BIRC2 protein expression (as demonstrated in Figure 2), I think that the authors could simplify the figure and remove the analysis of BIRC2 protein expression.

Author Response: As requested, we have deleted the protein expression analysis for BIRC2 when cells were treated with PS-1145. The relevant parts of the text have also been amended to reflect this change.

Since the conclusions of the result paragraph 3 and 4 (Figure 3 and 4) are the same, e.i. the induction of BIRC3 mRNA and protein expression in IL1B, TNF, IL1B + DEX and TNF + DEX treated cells depended on NF-kB, the two paragraphs could be combined.

Author Response: As requested, we have condensed the text to include the effect of NF-κB on BIRC expression as a single section of results.

Line 342: “Dexamethasone-induced BIRC3 mRNA expression was not changed by PS-1145”. I do not agree with this sentence. Although statistically non-significant, the modest induction of BIRC3 in response to dexamethasone (in the left and right panels) in decreased by PS-1145 (Figure 4).

Author Response: We thank the reviewer for this keen observation. We agree that there may be a genuine effect where cells treated with Dex + PS-1145 appear to result in a lower level of BIRC3 mRNA. However, we note that this effect was not significant. Nevertheless, inhibition of NF-κB may well reduce the basal expression of BIRC3 as supporting evidence can be seen in Figs 3C, 3D and 4A where there are also modest reductions in BIRC3 expression following NF-κB is inhibition in the unstimulated groups. We have revised the sentence that was flagged by the reviewer and have commented on the possible role of basal NF-κB activity, i.e. even in unstimulated cells.

3. The authors analyzed the stability of BIRC2 and BIRC3 proteins in TNFα and IL-1β treated cells (Figure 6). Cells were first treated with the agents for 1 hours, then incubated in the presence or absence of CHX. As expected, IL1b or TNF both enhanced BIRC3 protein expression. While 6 hrs of treatment with IL1B or TNFα did not modify the BIRC2 protein expression in Figure 2A and B, it seems increased BIRC2 protein expression in Figure 6B (quantitative analysis, lower panel)? Is that significant? Could authors explain these results?

Author Response: The modest effect on BIRC2 expression shown in Figure 6 that is mentioned by the referee was not significant. As noted by the reviewer, there is some variability when documenting BIRC2 expression throughout the manuscript. We believe that this is primarily due to the inherent variability in western blotting combined with small biological effects and relatively small samples sizes. It is worth noting that any changes in BIRC2 protein expression were less than 2-fold. Western blotting, as semi-quantitative, or comparative, technique (& depending on operator skill) is not really robust enough to clearly document such small expression changes. While some blots appear to support a slight increase in BIRC2, others don’t. Overall, any changes were difficult to consistently capture and this resulted in non-significant outcomes.

4. Figure 8C, left panel reproduced the results of Figure 6B. The authors could simplify and remove this panel. Since the authors demonstrated that IL-1β did not destabilize BIRC2 and BIRC3 protein in Figure 6B, the analysis the importance of proteasome inhibitor on destabilization of BIRC proteins in IL-1β treated cells is not relevant and this can be removed.

Author Response: With the aim of creating a concise manuscript, we have removed Figure 8C left- and middle-panel. As a consequence, several lines of text have been removed from this section.

Reviewer #2: The authors have adequately addressed all the comments. No further comments.
The authors have adequately addressed all the comments. No further comments.

Author Response: We thank the reviewer for this.
